# Spatiotemporal dynamics of traffic bottlenecks yields an early signal of heavy congestions

**DOI:** 10.1038/s41467-023-43591-7

**Published:** 2023-12-04

**Authors:** Jinxiao Duan, Guanwen Zeng, Nimrod Serok, Daqing Li, Efrat Blumenfeld Lieberthal, Hai-Jun Huang, Shlomo Havlin

**Affiliations:** 1 School of Economics and Management, Beihang University, Beijing, 100191 China; 2 Department of Physics, Bar-Ilan University, Ramat Gan, 52900 Israel; 3 School of Reliability and Systems Engineering, Beihang University, Beijing, 100191 China; 4 Azrieli School of Architecture, Tel Aviv University, Tel Aviv, 6997801 Israel

**Keywords:** Complex networks, Statistical physics

## Abstract

Heavy traffic jams are difficult to predict due to the complexity of traffic dynamics. Understanding the network dynamics of traffic bottlenecks can help avoid critical large traffic jams and improve overall traffic conditions. Here, we develop a method to forecast heavy congestions based on their early propagation stage. Our framework follows the network propagation and dissipation of the traffic jams originated from a bottleneck emergence, growth, and its recovery and disappearance. Based on large-scale urban traffic-speed data, we find that dissipation duration of jams follows approximately power-law distributions, and typically, traffic jams dissolve nearly twice slower than their growth. Importantly, we find that the growth speed, even at the first 15 minutes of a jam, is highly correlated with the maximal size of the jam. Our methodology can be applied in urban traffic control systems to forecast heavy traffic bottlenecks and prevent them before they propagate to large network congestions.

## Abstract (web summary)

Heavy traffic jams are difficult to predict due to the complexity of traffic dynamics. The authors propose a framework to unveil identifiable early signals and predict the eventual outcome of traffic bottlenecks, which may be useful for designing effective methods preventing traffic jams.

## Introduction

Urban traffic congestion^1,2^ is an everyday troubling phenomenon where many large traffic jams are triggered by uncertain congestion sources, also known as traffic bottlenecks^3^. The challenges of understanding complex congestion propagation have stimulated extensive traffic flow approaches to modeling and understanding urban traffic dynamics. Some of them investigated the spontaneous occurrences of congestion, including the kinematic wave theory^4,5^, the cellular automaton models^6,7^, and the three-phase traffic theory^8–10^. Attention is also paid to understanding the jam formation for the known causes, including the queue model^11^, the lane-changing model^12–14^, and the cell transmission model^15,16^ (see Supplementary Note 1). In recent years, emerging theories from other natural systems have been borrowed and applied to traffic systems for network’s propagation of traffic congestions, such as the cascading failure models^17–20^, epidemic models^21–23^, and congestion tree method^24,25^. Studies of traffic condition prediction^26–34^ and travel demand control^35–43^ also considered congestion propagation and suggested that understanding such spatiotemporal dynamics, especially their bottlenecks, could be effective for forecasting them and thus have the potential to prevent congestions from spreading^44^.

A recent study of ref. ^21^ formulated and validated the macroscopic propagation and dissipation of overall traffic congestions by a susceptible-infected-recovered model. Since macroscopic traffic congestions in the overall network are comprised of numerous fragmented congestion components^45^ that could be created by specific bottlenecks, overall traffic conditions could be improved by identifying the spatiotemporal dynamics of bottlenecks. Identifying traffic bottlenecks will not only help avoid themselves but also alleviate other associated congested roads due to network propagation. However, existing bottleneck methods^46–50^ characterizing the queues and delays associated with a specific traffic bottleneck are mostly based on the single-car particles in a simplified corridor^51^. Such methods may not be suitable for describing network dynamics of urban traffic bottlenecks. This is because urban congestions often exhibit strong spatial dependencies in which multiple-dimensional road segments adjacent to a bottleneck have a greater risk of becoming congested^1,20,21,45,52^. In the present study, we introduce a simple dynamic framework and demonstrate empirically that it can quantitatively capture the network’s propagation and dissipation of the congested components connected to specific traffic bottlenecks.

Many studies have provided important foundations to define or empirically identify urban traffic bottlenecks. The primary methods are based on the congestion level of a road segment itself. For example, a road with an average velocity lower than 20 km/h is regarded as the traffic bottleneck^3^. Network and percolation approaches^24,25,45,52–55^ have been recently developed to identify bottlenecks of traffic congestions by considering spatiotemporal traffic dynamics. For instance, a recent method has combined congestion propagation into a “tree” structure^24,25^ to identify the origins of congestions and traffic jams that are likely to stem from them in real-time traffic. Such methods referred to the earliest congested street as the “trunk” (or bottleneck), and the neighboring congested streets that developed in the following times represent the “branches” of the trunk. They found that bottlenecks associated with heavy congestion usually do not reappear on different days and hours. Luan et al.^26^ used Bayesian methods to infer the congestion propagation from a given congestion bottleneck. They found that a change in the location of congestion source can lead to distinctly different congestions. Thus, a suitable forecasting method is highly necessary to identify bottlenecks that will yield large and costly traffic jams. However, methodologies that address the identification of the most critical bottlenecks in order to stop them from growing to significant congestion components, are still missing.

In this paper, we quantitatively investigate the congestion propagation and dissipation which originate from specific traffic bottlenecks in traffic networks, and provide an early signal for forecasting the oncoming significant congestions. To this end, we follow the entire spatiotemporal process of each congestion component from its emergence as a bottleneck until its disappearance. We distinguish the evolution of traffic bottlenecks for each congestion component between the growth stage and recovery stage. We follow congestion dynamics that emerged from all bottlenecks in 1 month of traffic networks in two large cities in China, and find that the recovery duration of jams associated with each bottleneck follows a power-law distribution with very similar exponents in different days. Interestingly, we observed that the recovery duration of congestion components is typically nearly twice compared to its growth duration. Our study suggests an effective early predictive method of large jams. This method is based on our finding that the sizes of congestion components are highly correlated with their growth speed. In particular, we find that the growth speed of jams in their very early propagation stage, is also highly correlated with the maximal size of the jams, and therefore, this early speed can be used to predict major bottlenecks with high accuracy.

## Results

### Dynamics of traffic bottlenecks

We analyze real-time traffic datasets that include the time-dependent velocity of each road segment for one month in two large cities in China, Beijing and Shenzhen. The velocity matched to each road is aggregated from numerous trajectory records recorded by GPS devices in floating cars, with a resolution of 1 min^54^. The studied time periods are 00:00–24:00 for 30 days during October 2015 (for more details of the dataset, see Methods, Supplementary Note 2 and Supplementary Figs. 1 and 2). We identify the traffic bottlenecks based on the jam tree structure^24^ that assigned the earliest congested road downstream of the congestion component as the traffic bottleneck. The new congestions in neighboring upstream roads are associated with the bottleneck if they become congested no longer than a pre-defined time *θ* after the bottleneck or an existing downstream neighboring congestion in the component (see Methods).

**Fig. 1:**
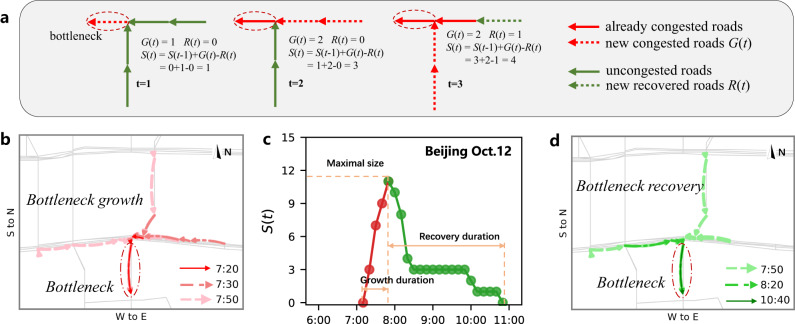
Demonstration of jam propagation and dissipation from a typical bottleneck in traffic network. **a** Demonstration of congestion size *S* associated with a bottleneck in an illustrated network. **b** Several snapshots of the congested road segments during the growth stage at 7:20, 7:30, and 7:50 for a specific but typical bottleneck on Monday, October 12, 2015, in Beijing. The congestions originated from the bottleneck (circled red solid link), and gradually developed to more congestion (light red dashed links) in the upstream neighboring streets. The arrows of the links are the directions of traffic flow. **c** The size *S* changes over time. This congestion component lasted for more than 3 h after its bottleneck emerged. Its maximal size *S*_*P*_ reached 11 road segments at 7:50, and then the size *S* began to decrease until it completely vanished at 10:50. Note that the recovery duration is much longer than the growth, which is a common feature of the bottleneck dynamics (see Fig. 2). **d** Several snapshots of the congested road segments during the recovery stage at 7:50, 8:20, and 10:40. The congestion component gradually dissolves after its maximal size (light green dashed links) as the road segments become no longer congested.

To trace the spatiotemporal propagation and dissipation of traffic bottlenecks in empirical traffic data, we follow a bottleneck jam from its emergence to disappearance and calculate the number of existing congested roads $S(t)$ associated with this specific bottleneck over its evolution (see Fig. 1a and Methods). This dynamic network process of congestions from a bottleneck can distinguish the time evolution of size *S* between two stages: growth and recovery stages. The peak time ${t}_{P}$ when size $S(t)$ of congestions connected to a bottleneck reaches maximum ${S}_{P}$ is regarded as the end of growth stage and the beginning of the recovery stage, see Fig. 1. We define the entire lifespan *T* of a bottleneck dynamics as the sum of the duration ${T}_{G}$ of the growth stage and the duration ${T}_{R}$ of the recovery stage, i.e.,

1 $$T={T}_{G}+{T}_{R}$$

here, the growth duration ${T}_{G}$ is the time interval between the earliest time when a bottleneck emerges and the time it reaches its maximal size, ${S}_{P}$, and the recovery duration ${T}_{R}$ is the time interval between the peak time and the time when the bottleneck jam has been completely dissolved and the traffic flows are uncongested again.

Figure 1 demonstrates the real-time spatiotemporal dynamics of a typical bottleneck on Monday, October 12, 2015, in Beijing, where three snapshots of the congested roads connected to the bottleneck during growth stage are displayed in Fig. 1b and three snapshots of the congested roads connected to the bottleneck during recovery stage are displayed in Fig. 1d. During the growth stage, increasing of the newly congested road segments associated with the bottleneck is faster than the recovered road segments, and this jam component propagates to its maximal size ${S}_{P}$ = 11 at 7:50 (Fig. 1b, c). After the peak time, the congestions began to dissolve and the size of the jam component started to decrease, reaching size *S* = 4 at 8:20, and size *S* = 1 at 10:40. By 10:50, the jam component dissolved completely and resulted in uncongested traffic. Once the traffic congestion created by a specific bottleneck has been dissolved, a new bottleneck may emerge from the same place to initiate another congestion component (more patterns of bottleneck dynamics are given in Supplementary Figs. 3 and 4).

Additionally, we find that the network’s propagation and dissipation of traffic congestions originated from a specific bottleneck, can be quantitatively described by a simple dynamical equation

2 $$\frac{dS(t)}{dt}=G(t)-R(t)$$

which is an extension of the classical bottleneck model^37,47,56^ that characterizes the queues from a bottleneck as the accumulations of single-car particles in a simplified corridor (see Supplementary Note 3 and Supplementary Fig. 5). Here, $G(t)$ and $R(t)$ are respectively the number of newly developed congested roads and the number of newly recovered roads associated with the bottleneck at time window *t* (see Supplementary Note 4 and Supplementary Figs. 6 and 7). Therefore, the existing size $S(t)$ in the congestion component at time window *t* is the integration of the difference between $G(t)$ and $R(t)$ from the time ${t}_{B}$ when a bottleneck *B* occurred up to current time window *t*, given by

3 $$S(t)={\int }_{{t}_{B}}^{t}(G(u)-R(u))du$$

### Distributions of growth and recovery durations

**Fig. 2:**
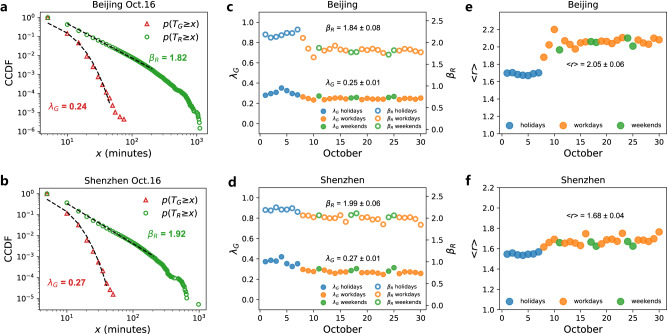
Distributions of growth duration and recovery duration. The CCDF of growth duration ${T}_{G}$ follows approximately an exponential distribution, and the CCDF of recovery duration ${T}_{R}$ follows approximately a power-law distribution. The data are taken for Friday, October 16, 2015, for (**a**) Beijing and (**b**) Shenzhen. We accumulate the data for all days in 1 month together and find CCDF of the growth duration above 40 min could be described by exponential distributions with smaller exponent ${\lambda }_{G}$ (Supplementary Fig. 24). The exponents ${\lambda }_{G}$ and ${\beta }_{R}$ for each day in the entire month for (**c**) Beijing and (**d**) Shenzhen. The values of the mean ratio $< r >$ for each day in the entire month for (**e**) Beijing and (**f**) Shenzhen. The days between October 1 and October 7 are the seven holidays of China’s National Day. October 11, 17, 18, 24, and 25 are the regular weekends, and the other 18 days are workdays. The mean values and standard deviations in subfigures (**c**–**f**) are calculated for 18 workdays. As the model selection analyses (Supplementary Note 8 and Supplementary Table 1) suggest, the *p* value and the well-fitted curves support that the exponential distribution describes well for the main part of ${T}_{G}$.

To explore the evolution durations of growth and recovery stages, we follow the dynamics (or lifespan) of each traffic bottleneck in the entire road networks of Beijing and Shenzhen during 1 month. To this end, we compute the vectors of growth duration ${T}_{G}=\{{T}_{G}^{1},\cdots,{T}_{G}^{N}\}$ and recovery duration ${T}_{R}=\{{T}_{R}^{1},\cdots,{T}_{R}^{N}\}$. Here, ${T}_{G}^{i}$ and ${T}_{R}^{i}$ are respectively the growth and recovery durations of bottleneck $i$, and *N* is the total number of bottlenecks, which is over 670,000 during a typical workday in Beijing and over 180,000 in Shenzhen. The distributions of durations ${T}_{G}$ and ${T}_{R}$ on Friday, October 16, 2015, in both cities are presented in Fig. 2a, b. The results show that the distribution of ${T}_{R}$ is significantly broader than that of ${T}_{G}$. Bottlenecks spend at most 100 min to reach the maximal size ${S}_{P}$, while they might spend up to 1000 min to recover to an uncongested state. The complementary cumulative distribution function (CCDF) of growth duration ${T}_{G}$ is well approximated by the exponential distribution

4 $$p({T}_{G}\ge x)\sim {e}^{-{\lambda }_{G}x}$$

with exponents ${\lambda }_{G}$ of 0.24 for Beijing and 0.27 for Shenzhen on Oct. 16. The CCDF of recovery duration ${T}_{R}$ is well approximated by the power law distribution

5 $$p({T}_{R}\ge x)\sim {x}^{-{\beta }_{R}}$$

with ${\beta }_{R}$ of 1.82 for Beijing and 1.92 for Shenzhen. We fit both the CCDF (Supplementary Figs. 8–11) and probability distribution (Supplementary Figs. 12 and 13) to describe the main part of ${T}_{G}$ and ${T}_{R}$, and find that they have similar distribution patterns in different days. In addition, CCDF of the ratio $r={T}_{R}/{T}_{G}$ of each congestion component follows also a power-law distribution $p(r\ge x)\sim {x}^{-{\beta }_{r}}$ with exponents ${\beta }_{r}$ around 2.1 ± 0.09 on workdays in Beijing and 2.16 ± 0.05 on workdays in Shenzhen (see Supplementary Figs. 14 and 15 for CCDF of *r*, and Supplementary Figs. 16, 17 and 18 for distribution of *r*). For sensitivity analysis, we absorb very short roads into the junctions or consider congestion length in the definition of congestion size *S*(*t*), and find that the CCDFs of the ${T}_{G}$ and ${T}_{R}$ are robust (Supplementary Note 5 and Supplementary Figs. 19, 20 and 21). We also study the bottleneck dynamics by smoothing the traffic conditions in different time intervals, and find CCDFs of ${T}_{G}$ and ${T}_{R}$ follow similar distributions (Supplementary Fig. 22).

The average ratio $< r >$ for all the congestion components is evaluated by

6 $$ < r > =\frac{1}{N}\mathop{\sum}\limits_{1\le i\le N}{r}_{i}=\frac{1}{N}\mathop{\sum}\limits_{1\le i\le N}\frac{{T}_{R}^{i}}{{T}_{G}^{i}}$$

where the values of $< r >$ are 2.06 for Beijing and 1.74 for Shenzhen on Oct. 16. This shows that, on average the recovery duration of a congestion component is 2.06 times of its growth duration in Beijing, and 1.74 times in Shenzhen. This demonstrates the strong asymmetry between network’s propagation and dissipation of bottlenecks, which could be explained by the theory of phase transition in traffic network flows. The propagation of urban traffic congestion has been regarded as the percolation in the system^54,57^, and the traffic systems have exhibited phase transitions as the traffic congestion components are close to the critical point in the self-organized process^7,45^. It has been found that the recovery of the systems against the perturbations can be slowing down, when the system is close to such critical point, also called “critical slowing down”^58–61^. As the results in our manuscript, the power-law distribution of the congestion dissipation indicates that the traffic systems might be near the critical points of the self-organized process. Thus, the slower recovery of the system could be the result of the “critical slowing down” mechanisms^62^.

Comparing growth and recovery stages on different days, we find that the exponential exponents ${\lambda }_{G}$ of growth duration and power law exponents ${\beta }_{R}$ of recovery duration are stable for the same type of days (Fig. 2c, d). This may indicate the stable self-adaptability of the traffic system in the dynamics of congestion evolutions^45^ (See Supplementary Note 6 for further discussions of the exponents). In Fig. 2e, f, we compare the average ratio $< r >$ between the recovery duration and growth duration on 30 days during October 2015 in both Beijing and Shenzhen. The results show that $< r >$ has a stable value for a specific type of day. In both cities, values of $< r >$ on workdays are larger than those on holidays. This means that on workdays, the recovery stage of the congestion components takes much longer time than their growth stage compared to holidays. The values of <*r*> could be influenced by the heavy tails of the recovery duration (Supplementary Note 7 and Supplementary Fig. 23). The “critical slowing down” effect^58^ becomes more significant for those bottlenecks with large recovery duration. This could be related to the fact that nonlinear mechanisms exist between the congestion propagation and dissipation processes given the complex urban traffic network topologies and the travelers’ real-time self-adaptive route choice behaviors^58,63,64^.

### Correlation between spatial and temporal dynamics

We further explore the relationship between temporal and spatial dynamics of traffic bottlenecks, by examining the correlation between the maximal size ${S}_{P}$ and the growth speed of the congestion components. For that, we define and calculate the average growth speed of congestion component associated with each bottleneck. The average growth speed ${V}_{A}$ is defined as the maximal size ${S}_{P}$ divided by its growth duration ${T}_{G}$, i.e.,

7 $${V}_{A}=\frac{{S}_{P}}{{T}_{G}}$$

**Fig. 3:**
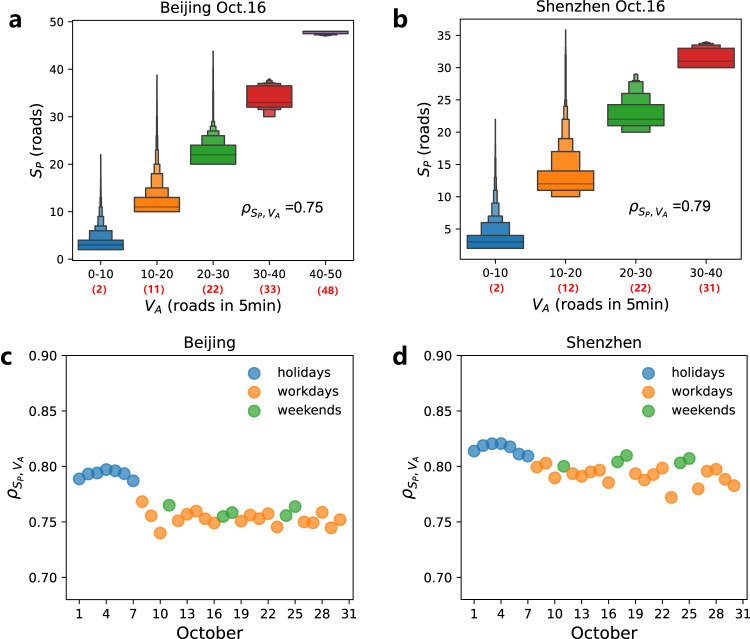
Correlation between the spatial maximal size *S*_*P*_ and the temporal growth speed of congestion components. Box plots of size ${S}_{P}$ grouped by average growth speed ${V}_{A}$ on Friday, October 16, 2015, in (**a**) Beijing and (**b**) Shenzhen. Here, ${V}_{A}$ means the average number of increased congested road segments every 5 min during the entire growth stage, the black numbers on the *x*-axis are the range of average growth speed ${V}_{A}$, and the red numbers in the bracket are the median value of each group. The black lines at the bottom, middle and top of the largest box are respectively 25%, 50%, and 75% percentiles of ${S}_{P}$. The tails of the distributions could be more clearly observed by more quantiles in 0–25% and 75–100% of ${S}_{P}$. The outliers are not displayed in the box plots. The growth duration ${T}_{G}$ increases with ${S}_{P}$ on average showing very large fluctuations, as shown in Supplementary Fig. 30. Pearson correlation ${\rho }_{{S}_{P},{V}_{A}}$ for the 30 days during October 2015 in (**c**) Beijing and (**d**) Shenzhen. Holidays include the 7 days in China’s National Day, and the workdays include 18 days. Bottlenecks with ${S}_{P}\ge 2$ are counted here. For sensitivity analysis, we absorb very short roads or consider congestion length in the definition of congestion size $S(t)$, and find that the growth speed of congestion components is still strongly and positively correlated to their maximal congestion size (Supplementary Note 5 and Supplementary Fig. 31).

A larger ${V}_{A}$ means that a bottleneck induces more associated congestions per unit time. Here, ${V}_{A}$ is calculated by the average number of the increased congested road segments in every 5 min. Figure 3a, b shows the relationship between ${S}_{P}$ and ${V}_{A}$ on October 16, 2015, in Beijing and Shenzhen (for similar results on other days, see Supplementary Figs. 25 and 26). These box plots of ${S}_{P}$ are classified into different groups by the values of ${V}_{A}$. It is seen that congestion components having a larger speed ${V}_{A}$ are more likely to reach a larger congestion size (In Supplementary Fig. 27, the positive relationship of ${V}_{A}$ and ${S}_{P}$ is also observed by plotting the density surfaces). The linear correlation between the maximal size ${S}_{P}=\{{S}_{P}^{1},\cdots,{S}_{P}^{N}\}$ and the average growth speed ${V}_{A}=\{{V}_{A}^{1},\ldots,{V}_{A}^{N}\}$ of these congestion components is also characterized by the high values of Pearson correlation coefficients ${\rho }_{{S}_{P},{V}_{A}}$. When omitting isolated bottlenecks with ${S}_{P}=1$, the value of ${\rho }_{{S}_{P},{V}_{A}}$ on Oct. 16, 2015 is 0.75 for Beijing and 0.79 for Shenzhen, indicating that ${S}_{P}$ and ${V}_{A}$ have a strong positive linear correlation.

Figure 3c, d shows that in both cities, the values of the correlation ${\rho }_{{S}_{P},{V}_{A}}$ are stable for the same type of days. The correlation coefficients are larger on holidays than on workdays, and also larger in Shenzhen than in Beijing. Note also that in the middle of holidays (days Oct. 3–5) the correlations are higher due to less traffic demand. We also find that the bottlenecks which create large congestions are less probable to sustain for a very long duration (Supplementary Figs. 28 and 29). This could be because drivers are strongly averse to very large congestions in the self-organization process of their game behavior, and would like to make adaptive choices to deviate from the long queues^65,66^. Moreover, well-developed traffic control technologies could have also focused on alleviating these heavy congestions^67^. Even so, as can be seen in the figures (Supplementary Figs. 28 and 29), very large congestions can still be sustained for hours.

### An early signal for forecasting heavy congestions

The average growth speed has been found in previous subsection to be correlated with the jam size. However, this correlation can only be determined at the end of the growth stage and thus it is not suitable for predicting heavy bottlenecks before their full growth.

In the present section, we develop an effective early signal for forecasting heavy traffic congestions based on the initial growth stage of jams. For that, we explore if the maximal size ${S}_{P}$ is correlated to the earliest attributes of the congestion propagation, i.e., the initial growth speed ${V}_{{T}_{I}}$, defined as the value of the early size ${S}_{{T}_{I}}$ divided by its initial growth time ${T}_{I}$ at an early growth stage. Thus, ${V}_{{T}_{I}}$ is expressed by

8 $${V}_{{T}_{I}}=\frac{{S}_{{T}_{I}}}{{T}_{I}}$$

where ${T}_{I}$ is the time interval between the emergence of a bottleneck and a selected early time before the congestion component reaches its maximal size, and ${S}_{{T}_{I}}$ is the number of congested roads connected to the bottleneck at the chosen early growth duration ${T}_{I}$.

**Fig. 4:**
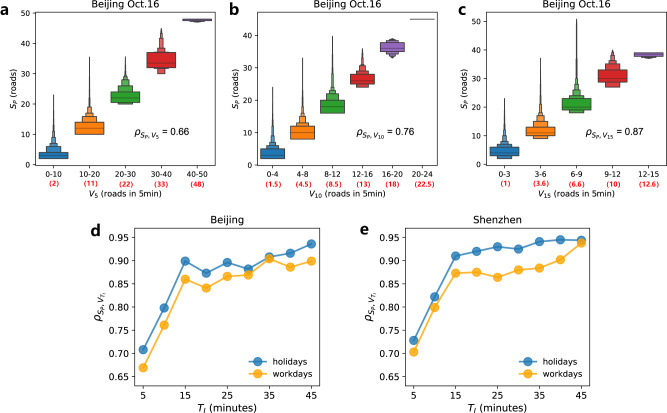
Correlation between the maximal size *S*_*P*_ and the initial growth speed ${V}_{{T}_{I}}$ at the earliest stage. Box plots of size ${S}_{P}$ grouped by initial growth speed ${V}_{{T}_{I}}$ at the earliest (**a**) 5, (**b**) 10 and (**c**) 15 min, on October 16, 2015, in Beijing. Here, ${V}_{{T}_{I}}$ means the number of increased congested road segments every 5 minutes during the early growth stage, the black numbers on the *x*-axis are the range of initial growth speed ${V}_{{T}_{I}}$, and the red numbers in the bracket are the median value of each group. The outliers are not displayed in the box plots. Pearson correlation ${\rho }_{{S}_{P},{V}_{{T}_{I}}}$ between maximal size *S*_*P*_ and the initial growth speed ${V}_{{T}_{I}}$ increases with increasing the initial growth duration ${T}_{I}$, for (**d**) Beijing and (**e**) Shenzhen. The correlations between ${S}_{P}$ and ${V}_{{T}_{I}}$ are calculated for the congestion components with growth duration longer than or equal to ${T}_{I}$ (see Methods). Bottlenecks with ${S}_{P}\ge 2$ are counted here. Note that above 15 min the correlations almost do not increase. Holidays include the 7 days in China’s National Day, and the workdays include 18 days.

As seen in Fig. 4a–c, the box plots of ${S}_{P}$ grouped by ${V}_{{T}_{I}}$ (${T}_{I}$ ranges from 5 to 15 minutes) show that on October 16, 2015, Beijing, the maximal size ${S}_{P}$ increases with ${V}_{{T}_{I}}$ at the first 5, 10, or 15 min of the bottleneck dynamics (box plots for Shenzhen show similar patterns, see Supplementary Fig. 32). This indicates that congestion components with a larger initial growth speed even at the earliest stage are also more likely to reach a larger congestion size. The correlation coefficients ${\rho }_{{S}_{P},{V}_{{T}_{I}}}$ between the maximal size ${S}_{P}=\{{S}_{P}^{1},\cdots,{S}_{P}^{N}\}$ and the initial growth speed ${V}_{{T}_{I}}=\{{V}_{{T}_{I}}^{1},\cdots,{V}_{{T}_{I}}^{N}\}$ are respectively 0.66, 0.76, and 0.87 for ${T}_{I}=5$, ${T}_{I}=10$ and ${T}_{I}=15$, which also supports our claim that the initial growth speed has a strong positive correlation with the maximal size ${S}_{P}$ (Fig. 4a–c). In addition, we calculate and compare the linear correlation ${\rho }_{{S}_{P},{V}_{{T}_{I}}}$ between the maximal size ${S}_{P}$ and the initial growth speed ${V}_{{T}_{I}}$ for different ${T}_{I}$ in workdays and holidays. As seen in Fig. 4d, e, the values of ${\rho }_{{S}_{P},{V}_{{T}_{I}}}$ for the two cities are around 0.65 to 0.75 for ${T}_{I}$ = 5 min, and increase to a higher plateau, around 0.85 to 0.95, when ${V}_{{T}_{I}}$ is obtained for 15 min and above. Note that correlations ${\rho }_{{S}_{P},{V}_{{T}_{I}}}$ in holidays are higher due to less traffic demand, which may lead to better prediction results. This suggests that the initial growth speed in the earliest 15 min, could be an effective predictor for the maximal size of congestions originated from a bottleneck, since the change in correlations is very little when ${T}_{I}$ is above 15 min.

### Forecasting performance for major bottlenecks

Before examining the performance of predicting major bottlenecks based on initial growth speed ${V}_{{T}_{I}}$, we evaluate the recurrence of the bottlenecks on two different days. This is done based on the Jaccard index $J$ that assesses the overlap (see Supplementary Fig. 33) of bottlenecks or their associated congestion components between 2 workdays. As shown in Supplementary Figs. 34, 35, and 36, the overlap of associated congestions is higher than the overlap of bottlenecks. This could be because the congestion propagation mechanism is fundamental^68^. Thus, the overall congestion regions could include similar propagation pathways due to the congestion propagation mechanism (Supplementary Note 9). Importantly, both Jaccard indexes ${J}_{B}$ for bottlenecks and ${J}_{C}$ for their associated congestions decrease rapidly with increasing jam size. ${J}_{B}$ decreases to around 0.05 and ${J}_{C}$ decreases to around 0.2 when ${S}_{P}$ is greater than or equal to 20, meaning that only around 5% of all the heavy bottlenecks and around 20% of their associated congestions (i.e., ${S}_{P}\ge 20$) that appear in both days are the same. Note that, similarities of large congestions are still very low even when we relax the overlap definition (Supplementary Note 10 and Supplementary Figs. 33 and 37). This could be because the traffic flows are the result of the dynamic self-organization of the game behaviors of numerous travelers^69–71^. In the day-by-day game process, due to risk aversion, travelers have been found to make adaptive choices and deviate from the congestions in previous days^72–75^. Thus, large congestion events could be less recurrent than expected, and heavy bottlenecks or their associated congestions in one day cannot represent much of those in the other days.

**Fig. 5:**
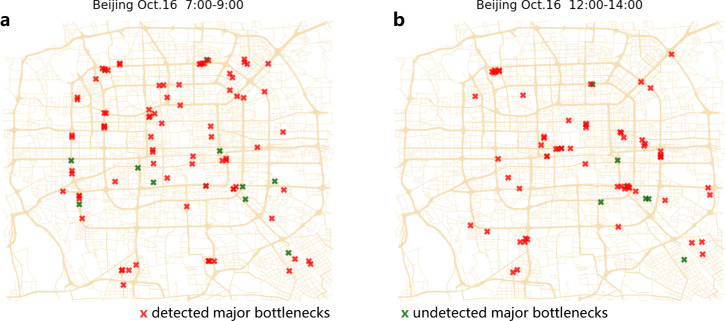
The detected major bottlenecks (true positive) and the undetected major bottlenecks (false negative) when the FPR is set as 5%. Major bottlenecks (${S}_{L}=20$) during (**a**) rush hours (7:00 a.m.–9:00 a.m.) and (**b**) non-rush hours (12:00 p.m.–14:00 p.m.) on October 16, 2015, in Beijing. The red stars are the true positive bottlenecks that are predicted as major bottlenecks and actually grew to be a major size. The green stars are the false negative bottlenecks that are predicted to be minor bottlenecks but actually developed to be major. The CCDF of maximal jam size ${S}_{P}$ is seen in Supplementary Note 11 and Supplementary Fig. 39. The CCDF of jam duration $T$ for the large congestion components is seen in Supplementary Note 11 and Supplementary Fig. 40.

We define a major bottleneck (i.e., $y=1$) as the bottleneck that developed into a congestion component with size ${S}_{P}$ greater than or equal to a pre-defined threshold ${S}_{L}$, and regard it as a minor bottleneck (i.e., $y=0$) otherwise. To test the power of predicting major bottlenecks by ${V}_{15}$, we trained the binary Probit model based on dynamics of all the bottlenecks on Monday, October 12, 2015, in Beijing (see Methods for details of the prediction method). We applied the trained model to predict whether bottlenecks on another workday, i.e., October 16, 2015, could yield congestion components greater than or equal to ${S}_{L}=20$. We find that when the false positive rate (FPR) is set to 5%, the accuracy of detecting major bottlenecks is around 88%. The detected and the undetected major bottlenecks displayed in Fig. 5 indicate that most of the major bottlenecks in rush and non-rush hours are different, and an appropriate prediction framework at the early propagation stage is thus highly needed. Additionally, examples of false positive bottlenecks are also located in significantly different road segments in rush and non-rush hours (see Supplementary Fig. 38).

**Fig. 6:**
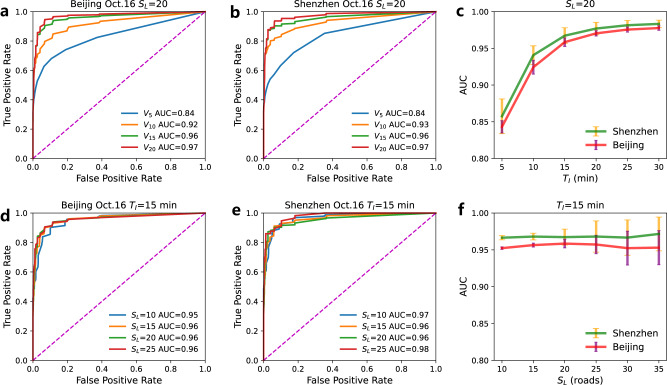
Performance of predicting major bottlenecks based on initial growth speed. ROC curves of predicting whether a bottleneck would grow up into a major congestion size of 20 road segments or above, based on ${V}_{{T}_{I}}$ within the earliest 5, 10, 15, and 20 min of their propagation. The prediction results shown are for October 16, 2015, in (**a**) Beijing and (**b**) Shenzhen. **c** The AUC value increases when the predictor ${V}_{{T}_{I}}$ is calculated based on a longer initial duration ${T}_{I}$. The error bars are calculated based on the prediction results of 17 workdays. The small standard deviations show that the values are stable. Note the almost plateau seen for ${T}_{I}\ge 15$ minutes.The ROC curves of using ${V}_{{T}_{I}}$ within 15 min to predict whether a bottleneck develops into at least 10, 15, 20, and 25 congested roads. The curves are based on the prediction results for October 16, 2015, in (**d**) Beijing and (**e**) Shenzhen. **f** The values of AUC for predicting major bottlenecks by ${V}_{{T}_{I}}$ within 15 min with different size thresholds ${S}_{L}$. The error bars are calculated based on the prediction results in 17 workdays. The small standard deviations show that the prediction performances are stable. The prediction results are also found stable when training the model on different days. For sensitivity analysis, we absorb very short roads or consider congestion length in the definition of congestion size *S*(*t*), and find robust prediction performance (Supplementary Note 5 and Supplementary Fig. 44).

Performances of predicting the major bottlenecks (${S}_{L}=20$) are assessed next for different values of FPR, for ${V}_{5}$, ${V}_{10}$, ${V}_{15}$, and ${V}_{20}$. In Fig. 6a, b, the receiver operating characteristic (ROC) curves^76^ are obtained and plotted based on the prediction results for Friday, October 16, 2015, in both Beijing and Shenzhen. The ROC curves indicate that the predictions based on initial growth speed within 15 minutes of the congestion components are close to the classifier (0,1) for both cities (ROC curves of other days are similar and presented in Supplementary Figs. 41 and 42). We also computed the area under the curve (AUC) for 17 workdays in both cities, as presented in Fig. 6c. The values of AUC are stable for different days and increase when a longer duration ${T}_{I}$ is used to calculate the predictor, i.e., the initial growth speed. These results suggest that initial growth speed of the congestion components within its first 15 minutes is a very reliable predictor for forecasting major congestions. Beyond 15 min, the performance of the predictions does not improve significantly. Notably, as seen in Supplementary Note 12 and Supplementary Fig. 43, the prediction time has a trade-off with prediction accuracy. In practice, the predictors ${V}_{5}$, ${V}_{10}$, and ${V}_{15}$ could be comprehensively applied to early identify the heavy bottlenecks before maximal size or full jam duration.

To further assess the prediction performance of the growth speed in the earliest 15 min, we forecast whether the bottlenecks will develop to be greater than or equal to different threshold size ${S}_{L}$. As seen in Fig. 6d and e, the ROC curves of predicting major bottlenecks for different ${S}_{L}$ are similar and very close to the classifier (0,1) on October 16, 2015 in both cities. We also computed in Fig. 6f, the AUC values versus the predefined threshold size ${S}_{L}$ for 17 workdays in both cities. It is seen that the values of the AUC are stable and very high (around 0.95) for all values of ${S}_{L}$. These results strongly support that the initial growth speed is a reliable and excellent measure for detecting major bottlenecks at their early stage of propagation. Thus, our prediction framework helps to understand, forecast, and potentially prevent major bottlenecks by identifying them at their early propagation stages. Integrating this framework into existing traffic control systems, may improve traffic conditions and prevent bottlenecks from reaching their full potential.

## Discussion

In summary, we analyze the spatiotemporal dynamics of traffic bottlenecks during 1 month in two Chinese metropolises. The empirical results demonstrate that our framework of dynamics of traffic bottlenecks can successfully characterize both the network’s propagation and dissipation of urban traffic bottlenecks. We find that the propagation and dissipation processes of congestion components develop asymmetrically and the duration of recovery stage approximately follows a power-law distribution. In addition, the recovery duration of the bottlenecks is found to be typically nearly twice the time it takes to fully grow. Our findings can provide data explanations for the well-established models and help extend mesoscopic traffic models in urban networks. For example, the scaling patterns of the jam durations in real urban networks can well explain and expand the 1-dimensional scaling results modeled in existing studies^77,78^.

Moreover, the empirical results suggest that our method is efficient in forecasting heavy traffic bottlenecks, based on the finding that the spatial and temporal dynamics of traffic congestions are strongly correlated. Our empirical analysis shows that the growth speed of congestion components is highly correlated with the maximal size of their associated congestions. Most importantly, we find that the initial growth speed, even at the earliest 15 min of the jam’s growth, is useful in predicting whether a traffic bottleneck will develop into a major congestion component. Our predictive framework can provide an alerting indicator that could help urban planners improve real-time traffic control by addressing the sources of destructive traffic jams before they develop into their maximal size. The early real-time traffic control began with the implementation of the traffic signal control system SCOOT to calculate the size of the queue and time of clearance given the cyclic flow profiles^79^. With artificial intelligence technologies, and connected-automated vehicles techniques^80–82^, the model predictive control framework has been proposed to sense and control traffic conditions while agents interact with ones in the vicinity^67,83–86^. Meantime, the spatial congestion propagation could significantly influence the coordinated control performances among different signalized intersections^87–89^ (Supplementary Note 13). Based on our identified spatiotemporal propagation mechanisms, the real-time control systems could consider interrelations among different traffic regions and perform better coordinated control for the most congested areas before they propagate to a global gridlock jam.

To better alleviate traffic congestions, our framework and findings can also be applied in the well-developed real-time navigation systems^90,91^ or demand management strategies^63,64,69,71,73,75,92^. Urban traffic control systems should be connected to navigation apps to warn travelers, in near real-time, to avoid the roads that may lead to a heavy traffic bottleneck^63,64,69,93^. On one hand, warning travelers with alternative routes to be far away from the predicted heavy congestion regions^73,75^ could alleviate the traffic flows in predicted areas. On the other hand, rescheduling departure time of the potential travelers with no alternative routes by road pricing strategies^71^ could reduce traffic demand in the predicted area. These methods of demand management have also been found effective in alleviating congestion in earlier studies^63,64,93^. Moreover, coordinated management from the supply side could also help to alleviate the traffic on the predicted heavy congestions^69^. While such interventions may cause congestions in other locations, our real-time updated framework can alert the control system about new heavy bottlenecks before they emerge. Considering our propagation mechanism in the above well-established technologies, the real-time traffic management and organization would be better developed in the near future. This will provide not only accurate predictions but also effective mitigation of heavy congestions.

## Methods

### Dataset

The Beijing road network has over 52,000 road segments (links) and 27,000 intersections (nodes), and the Shenzhen road network has over 22,000 road segments (links) and 12,000 intersections (nodes). The empirical datasets contain time-dependent velocity records of both cities for 30 days during October 2015 with a resolution of 1 minute. For a road with an outlier of velocity record, we handle it as congested state if it connects its upstream and downstream roads that are congested in the predefined chronological order (Supplementary Note 2 and Supplementary Fig. 1). The distribution of road velocity suggests that the road velocity records are generally stable over 5-minute interval (Supplementary Note 2 and Supplementary Fig. 2). Large-scale network topology, huge traffic flow, serious traffic jams, diverse traffic bottlenecks, as well as the high-quality datasets make these two cities ideal for the analysis of bottleneck dynamics.

### Bottlenecks and their associated congestions

Since traffic congestion state at time *t* can be described by the average relative velocity during a short time interval^52^, we use the average relative velocity calculated in every 5 minutes to represent the congestion weight ${W}_{e}(t)$ of road $e$ at time window $t$, i.e.,

9 $${W}_{e}(t)=\frac{{U}_{e}(t)}{{U}_{e}^{95}}$$

Here, ${U}_{e}(t)$ is the velocity of road $e$ at time window *t*, and ${U}_{e}^{95}$ is 95% percentile of velocity records of road *e*, which approximates the standard maximal velocity on this road. In this paper, road $e$ is regarded as congested if ${W}_{e}(t) < 0.5$, and non-congested otherwise^26^.

After having the congestion weight ${W}_{e}(t)$, we identify bottlenecks using the jam tree structure defined by ref. ^24^. We identified the bottlenecks, and the newly congested or the recovered roads from them as the following steps: *i*) Obtaining the time-interval matrix of the continuous congestion states. ${c}_{e}(t)$ is the time interval during which road *e* has been continuously congested up to the time window $t$. *ii*) Identification of bottleneck. The bottleneck is the road segment that has been continuously congested for the longest interval ${c}_{e}(t)$ in downstream of a congestion component. *iii*) Identification of newly congested road segments. A neighboring upstream congested road is regarded as a new congestion associated with a bottleneck at time window *t* if it became congested no later than a predefined time interval $\theta$ (set as 10 min generally) after the bottleneck or another congested road connected to the bottleneck. *iv*) Identification of recovered road segments. A road is regarded as recovered from the bottleneck at time window *t*, if it (or another congested road that connected it to the bottleneck) returned to an uncongested state (see Supplementary Note 4 and Supplementary Fig. 6). The existing number of congested roads, i.e., size *S*(*t*), includes newly congested links and removes those recovered. In the recovery process, the existing size *S* is decreasing over time since the newly recovered congested roads occur more quickly than newly developed congestions (Supplementary Note 4 and Supplementary Fig. 7). In our algorithm, if the recovery of the bottleneck is earlier than other neighboring branches, the branches that belonged to the recovered bottleneck are not considered anymore to be associated with the original (recovered) bottleneck.

### Pearson correlation

The Pearson coefficient represents the degree of linear correlation between two variables. To observe the linear correlation between the maximal size ${S}_{P}$ and the average growth speed ${V}_{A}$ or the initial growth speed ${V}_{{T}_{I}}$, we calculated Pearson correlation between them. For two variables $X=\{{x}_{1},{x}_{2},\cdots,{x}_{n}\}$ and $Y=\{{y}_{1},{y}_{2},\cdots,{y}_{n}\}$, the Pearson correlation ${\rho }_{X,Y}$ is

10 $${\rho }_{X,Y}=\frac{{{{{{\mathrm{cov}}}}}}(X,Y)}{{\sigma }_{X}{\sigma }_{Y}}=\frac{E[(X-{\mu }_{X})(Y-{\mu }_{Y})]}{{\sigma }_{X}{\sigma }_{Y}}$$

where *n* is the number of samples, ${\mu }_{X}$ and *μ*_*Y*_ are the mean values of *X* and *Y*, and ${\sigma }_{X}$ and ${\sigma }_{Y}$ are the standard deviations of *X* and *Y*. ${\rho }_{X,Y}$ is in the range of $-1$ to 1. ${\rho }_{X,Y}=\pm 1$ means variables *X* and *Y* have the highest linear correlation. When calculating ${\rho }_{{S}_{P},{V}_{{T}_{I}}}$ for congestion components with growth duration longer than or equal to ${T}_{I}$, the samples are downsized in a short ${T}_{I}$ and upsized in a long ${T}_{I}$ by observations obtained from different resolutions.

### Jaccard index

The Jaccard index is used for measuring repetition between the bottlenecks or their associated congestions on different days. For two sample sets *X* and *Y*, the Jaccard index is defined as the ratio between the size of the overlap and the size of the union of the observed sample sets, i.e.,

11 $$J=\frac{|X\cap Y|}{|X\cup Y|}=\frac{|X\cap Y|}{|X|+|Y|-|X\cap Y|}$$

where $J$ is in the range of 0 to 1. $J=1$ means sets *X* and *Y* are wholly overlapped and $J=0$ means sets *X* and *Y* are wholly different.

### Prediction method

To conduct the prediction, we train the binary Probit model (a basic binary classifier) based on the evolutions of traffic bottlenecks on Monday, October 12, 2015, and use the initial growth speed ${V}_{{T}_{I}}$ till an early growth time ${T}_{I}$ to predict the probability ${P}_{i}$ that a bottleneck on another workday may develop into a major congestion component. The utility function (${y}_{i}^{\ast }$) of bottleneck *i* with the explanatory variable ${V}_{{T}_{I}}$ is expressed as

12 $${y}_{i}^{\ast }={a}_{1}+{a}_{2}{V}_{{T}_{I}}^{i}+{\varepsilon }_{i},\,{y}_{i}=1\,if\,{y}_{i}^{\ast } \, > \, 0$$

where ${a}_{1}$ is a constant, ${a}_{2}$ is a coefficient, and ${\varepsilon }_{i}$ is the error term assumed to follow a standard normal distribution. Based on the estimated ${\hat{a}}_{1}$ and ${\hat{a}}_{2}$, the probability for ${y}_{i}=1$ is expressed as

13 $${p}_{i}({y}_{i}=1|{V}_{{T}_{I}})={p}_{i}({y}_{i}^{\ast } > 0)={p}_{i}({\varepsilon }_{i} > -({\hat{a}}_{1}+{\hat{a}}_{2}{V}_{{T}_{I}}^{i}))=\varPhi ({\hat{a}}_{1}+{\hat{a}}_{2}{V}_{{T}_{I}}^{i})$$

where *Φ* is the cumulative density function (*cdf*) of the standard normal distribution. Prediction based on ${V}_{{T}_{I}}$ is conducted in the following way: the probability ${P}_{i}$ of whether a bottleneck would become major till the time ${T}_{I}$, is calculated as the highest possibility that a bottleneck may become major based on ${V}_{{T}_{I}}$ till time ${T}_{I}$, i.e., ${P}_{i}=\,\max \{{p}_{i}({y}_{i}=1|{V}_{t})\},\,t\le {T}_{I}$. If the probability ${P}_{i}$ reaches a threshold ${P}^{thre}$, a bottleneck is classified as major. The prediction result ${\gamma }_{i}$ of a bottleneck could be true positive (TP), false positive (FP), true negative (TN), or false negative (FN), i.e.,

14 $${\gamma }_{i}=\left\{\begin{array}{c}TP,\,{P}_{i}\ge {P}^{thre}\,{{{{{\rm{and}}}}}}\,{y}_{i}=1\\ FN,\,{P}_{i} < {P}^{thre}\,{{{{{\rm{and}}}}}}\,{y}_{i}=1\\ TN,\,{P}_{i} < {P}^{thre}\,{{{{{\rm{and}}}}}}\,{y}_{i}=0\\ FP,\,{P}_{i}\ge {P}^{thre}\,{{{{{\rm{and}}}}}}\,{y}_{i}=0\end{array}\right.$$

When obtaining the prediction results $\gamma$ for different threshold ${P}^{thre}$, the ROC curve can be plotted by the results of the true positive rate (TPR) versus the false positive rate (FPR). The *x*-axis is the FPR, representing the ratio between the number of minor bottlenecks wrongly categorized as positive and the total number of actual minor bottlenecks. The *y*-axis is the TPR, representing the ratio between the number of major bottlenecks correctly categorized as positive and the total number of actual major bottlenecks. i.e.,

15 $$FPR=\frac{{N}_{FP}}{{N}_{FP}+{N}_{TN}}$$

16 $$TPR=\frac{{N}_{TP}}{{N}_{TP}+{N}_{FN}}$$

where ${N}_{FP}$ is the number of FP observations, ${N}_{TN}$ is the number of TN observations, ${N}_{TP}$ is the number of TP observations, and ${N}_{FN}$ is the number of FN observations. The AUC represents the performance of separability that the classifier can distinguish between the major and minor bottlenecks. The curve through (0,1) and AUC = 1 represent that the classifier has the best performance to separate each bottleneck correctly.

### Supplementary information

Supplementary Information

Peer Review File

## Data Availability

The necessary data generated in this study have been deposited in the Github database https://github.com/JinxiaoDuan/DynamicsOfBottlencks to enable the reproducibility of the results in the paper. The raw velocity data are protected and unavailable due to data privacy laws.

## Code availability

We provide the source codes of our algorithms in GitHub https://github.com/JinxiaoDuan/DynamicsOfBottlencks to enable the reproducibility of our findings.
