# Supplementary material for: Spatiotemporal dynamics of traffic bottlenecks yields an early signal of heavy congestions
Source: Nat Commun. 2023 Dec 4;14:8002. doi: 10.1038/s41467-023-43591-7 (PMC10695996; doi:10.1038/s41467-023-43591-7)
Supplement: Supplementary file 1 — Supplementary Information [file 41467_2023_43591_MOESM1_ESM.pdf]

Supplementary information for

Spatiotemporal dynamics of traffic bottlenecks yields an early signal of heavy congestions

Jinxiao Duan [1, 2], Guanwen Zeng [2, 3], Nimrod Serok [4], Daqing Li [3], Efrat Blumenfeld Lieberthal [4, *], Hai-Jun Huang [1, *], Shlomo Havlin [2, *]

[1] *School of Economics and Management, Beihang University, Beijing 100191, China.*

[2] *Department of Physics, Bar-Ilan University, Ramat Gan 52900, Israel.*

[3] *School of Reliability and Systems Engineering, Beihang University, Beijing 100191, China.*

[4] *Azrieli School of Architecture, Tel Aviv University, Tel Aviv 6997801, Israel.*

[*] Correspondence should be addressed to:
efratbl@tauex.tau.ac.il; haijunhuang@buaa.edu.cn; havlins@gmail.com

The SI file includes:

Supplementary Notes 1 to 13,

Supplementary Figures 1 to 44,

Supplementary Table 1,
and Supplementary References 1 to 39.

Supplementary Note 1. Related works on traffic congestion occurrences and formations.

On one hand, traffic flow theories have been developed and investigated to understand the spontaneous occurrences of congestion. The kinematic wave theory[1, 2] explained the transition from free flow to congestion as the result of wave interactions. The cellular automaton (CA) models[3, 4] found that traffic jams can spontaneously form when the density is high enough. The three-phase traffic theory[5-7] showed that traffic phase transition can occur in free flow, synchronized flow, and congested phases. On the other hand, attention has been also paid to understanding the jam formation of known causes. The queue model[8] studied the congestion formation nearby a bottleneck when its capacity is reduced or demand is increased. The lane-changing model[9-11] described the decision-making behavior when some lanes are blocked or closed. A simplified theory of kinematic waves proposed by Newell[12] analyzed the congestion hysteresis phenomenon for a highway exit/entry. The cell transmission model (CTM)[13, 14] considered congestions as a series of jam cells against a temporary obstacle, such as vehicle collision.

Supplementary Note 2. Details for the road velocity records.

We regard the road velocity record as an outlier point if it is missing for a specific instant in our dataset, which could be due to few floating cars passing through the road at the specific time. The outliers of the velocity records in the main roads account for around 3-5% of their total velocity records at most time (6:00 am-24:00 pm and 0:00 am-2:00 am) and 10-20% at midnight (2:00 am-6:00 am). When handling outliers, we regarded a road with outlier velocity as congestion state, if it connects its upstream and downstream roads that have become congested in the predefined chronological order. Specifically, for an outlier road, if its downstream road has been congested earlier than its upstream road, with the difference of their congestion time smaller than a predefined time-interval, i.e., 2θ (θ set as 10 minutes generally) (Supplementary Fig. 1), it is regarded as congestion state. Otherwise, it is regarded as uncongested. In general, around 2% of the outliers have been assigned as congested states.

To plot the distribution of velocity over a 5-minute interval for all the roads, we first rescale the velocity records in the given 5-minute interval of a road to be the relative deviation of the velocity from its mean. The rescaled velocity $U_e^S(i)$ of road segment e at minute i ($i=1,2,3,4,5$) is

$$U_e^S(i)=\frac{U_e(i)-U_e^a}{U_e^a}, \tag{1}$$

where $U_e(i)$ is the actual velocity record of road e at minute i, and U_e^a is the mean value of

the actual velocity records of road e in the given 5-minute interval. In Supplementary Fig. 2, it is seen that the empirical distribution of U_e^S shows a somewhat fat-tail feature compared with the fitted Gaussian distributions during both typical periods. The p-values of the Kolmogorov-Smirnov KS statistic[15] are $p < 0.0001$ for all the estimated distributions, indicating that the rescaled velocity deviates from the Gaussian distribution. The tails of the actual data are somewhat broader than the estimated Gaussian distributions, meaning that a small fraction of velocity records may have a large deviation from its mean value over 5-minute interval. However, the standard deviations are relatively small, indicating that the road velocity records are generally stable over a 5-minute interval.

Supplementary Note 3. Traffic bottleneck model based on the single-car particles in a simplified corridor.

The classical traffic bottleneck models[16-18] assume that a fixed number of travelers (one per car) depart from home and arrive at workplace during morning rush hour on a single road corridor (Supplementary Fig. 5a). Traffic conditions are assumed to be unblocked except at the single bottleneck where at most C car particles can pass each unit time. In such model, C is named as the capacity or the service rate of the bottleneck. If the arrival demand rate D at the bottleneck exceeds capacity C, a queue will emerge. With the arrival demand rate $D(t)$ at the bottleneck, the number $Q(t)$ of car particles in the queue (Supplementary Fig. 5b) at time t given by the classical bottleneck model is

$$Q(t) = \int_{t_q}^{t} D(u)du - C(t - t_q), \tag{2}$$

where t_q is the beginning time of the congestion period. The dynamic process of the bottleneck in the classic bottleneck model was given by

$$\frac{dQ(t)}{dt} = D(t) - C, \qquad \text{for } t_q < t < t_q^{'}, \tag{3}$$

where $t_q^{'}$ is the end time of the congestion period.

Supplementary Note 4. Formulating network dynamics of traffic bottleneck.

Urban congestions often exhibit high spatial dependencies where multiple-dimensional road segments adjacent to a bottleneck are more likely to become congested. Additionally, traffic conditions can be evaluated by the number of congested links in the traffic network[19-22]. The classical bottleneck model (Supplementary Note 3) in a single-road corridor may not be suitable to describe the network's link-to-link dynamics of the congestions from a traffic bottleneck.

To quantitatively describe network's propagation (e.g., from $t_B = 0$ to $t_P = 3$ in Supplementary Fig. 6b) and dissipation (e.g., from $t_P = 3$ to $t_B^{'}$ in Supplementary Fig. 6b) of traffic congestions originated from a specific bottleneck, we extend the classical bottleneck model that quantifies the queues from a bottleneck as the accumulations of single-car particles (Supplementary Note 3) to an ordinary differential equation (ODE) that describes network dynamics of a traffic bottleneck (see demos in Supplementary Fig. 6a), i.e.,

$$\frac{dS(t)}{dt} = G(t) - R(t), \tag{4}$$

where $S(t)$ is the existing number of congested roads connected to a specific bottleneck at time t, $G(t)$ is the number of newly congested roads developed from bottleneck at time t, and $R(t)$ is the number of roads that are newly recovered from the congestion component at time t. $S(t)$ will increase with time t if $G(t) > R(t)$, and decrease otherwise. Therefore, $S(t)$ at time t is the integration of the increased congested roads from the time t_B when a bottleneck B occurred up to current time t, given by

$$S(t) = \int_{t_B}^{t} \left(G(u) - R(u)\right)du. \tag{5}$$

This dynamic network process of the ODE can distinguish the time evolution of size S as a function of time between two stages: growth and recovery stages (Supplementary Fig. 6b). The peak time t_P, when size $S(t)$ of congestions connected to a bottleneck reaches maximum S_P, is regarded as the end of growth stage and the beginning of the recovery stage, i.e.,

$$t_P = \arg\max\{S(t)\} = \arg\max\{\int_{t_B}^{t} \left(G(u) - R(u)\right)du\}. \tag{6}$$

Therefore, the growth duration T_G is the time interval between the emergence time t_B of the bottleneck and its peak time t_P, and the recovery duration T_R is the time interval between the peak time t_P and dissipation time $t_B^{'}$ of the bottleneck (Supplementary Fig. 6b). Note that in the recovery stage (Supplementary Fig. 7), the size $S(t)$, i.e., the number of existing congested roads, includes newly developed congested roads and removes those recovered. Thus, the existing size S is decreasing over time in the recovery process due to more new recoveries than new congestions.

Supplementary Note 5. Sensitivity analyses by different definitions of congestion size.

The analyses conducted in the main text are based on the jam size S including all roads. To make sensitivity analyses of the results, here, we also conduct analysis based on the definition of size S by absorbing the very short roads or considering the congestion length.

The results support robustness of our conclusions.

(1) When absorbing the very short roads into the junctions, we define the absorbed congestion size $S_a(t)$ of a jam component as the number of associated congested roads longer than or equal to 50 meters (Supplementary Figs. 19a and b). The new results indicate that the patterns of the bottleneck dynamics and the performances of the predictions are robust.

(*i*) The CCDFs of growth duration and recovery duration identified by the absorbed size $S_a(t)$ (Supplementary Figs. 20a and b) are very similar to those obtained by full size $S(t)$ in the main text (Supplementary Figs. 20e and f). The stable exponents λ_G^a of the CCDF of growth duration (blue circles in Supplementary Figs. 21a and b) and stable exponents β_R^a of the CCDF of the recovery duration (blue circles in Supplementary Figs. 21c and d), support our finding in the main text that distributions of the congestion duration are regular in different days (orange triangles in Supplementary Fig. 21).

(*ii*) The Pearson correlations $\rho_{S_P^a,V_A^a}$ between the maximal absorbed congestion size and the average growth speed in the new analysis (blue circles in Supplementary Fig. 31) are slightly lower than the ρ_{S_P,V_A} in the main text (orange triangles in Supplementary Fig. 31). This supports our finding that maximal jam size is highly and positively correlated to their growth speed.

(*iii*) The AUC values are around 0.95 for the predictions in the new analyses (blue circles in Supplementary Fig. 44). This indicates the prediction results of the initial growth speed obtained by absorbed size, exhibit stable and good performance as in the main text (orange triangles in Supplementary Fig. 44).

(2) When considering the sum of length as definition of size S (Supplementary Figs. 19b and c), the new results also indicate that the patterns of the bottleneck dynamics and the performances of the predictions are robust.

(*i*) The CCDFs of growth duration and recovery duration identified by the congestion length $S_l(t)$ (Supplementary Figs. 20c and d) are very similar to those obtained by full size $S(t)$ in the main text (Supplementary Figs. 20e and f). The stable exponents λ_G^l of the CCDF of growth duration (green triangles in Supplementary Figs. 21a and b) and stable exponents β_R^l of the CCDF of the recovery duration (green triangles in Supplementary Figs. 21c and d), support our finding in the main text that distributions of the congestion duration are regular in different days (orange triangles in Supplementary Fig. 21).

(*ii*) The Pearson correlations $\rho_{S_P^l,V_A^l}$ between the maximal congestion length and the average growth speed in the new analysis (green triangles in Supplementary Fig. 31) are slightly higher than the ρ_{S_P,V_A} in the main text (orange triangles in Supplementary Fig. 31).

This supports our finding that maximal jam size is highly and positively correlated to their growth speed.

(*iii*) The AUC values are around 0.95 for the predictions in the new analyses (green triangles in Supplementary Fig. 44). This indicates the prediction results of the initial growth speed obtained by congestion length, exhibit stable and good performance as in the main text (orange triangles in Supplementary Fig. 44).

Supplementary Note 6. Discussion of exponents β_R in different days.

The values of β_R on non-holidays are lower than the values on holidays, whereas the values are not significantly different between workdays and weekends, as shown in Fig. 2 in the main text. The differences in exponent β_R could be due to traffic flow patterns. In the megacities Beijing and Shenzhen, the workdays and weekends experienced higher traffic demands by local travelers, whereas the holidays experienced less travel demands by mainly the tourists. The workdays experienced high traffic demands due to the great number of commuting trips for local residents. Regular weekends experienced a great number of traffic demands by partially leisure trips for local residents. The higher demand possibly induces more high-flow roads and more long-duration congested roads, which could bring to large tails of the duration distributions and therefore smaller exponents. However, the representative national holiday, as one of the largest holidays, experienced significantly reduced travel demand and significantly different demand patterns by mainly the tourists.

Supplementary Note 7. Discussion of ratio *r* in different ranges of recovery duration T_R.

We have performed additional analysis and found that the average ratio <*r*> between recovery duration T_R and growth duration T_G could be influenced by the heavy tail in recovery duration distribution. It is shown in Supplementary Figs. 23a and b that, the upper bounds of the ratio *r* for bottlenecks with $T_R \geq 30$ are much larger than that for $T_R < 30$. In Supplementary Figs. 23c and d, it is shown that the average ratio <*r*> for bottlenecks with $T_R \geq 30$ are stable in different workdays and larger than the values for bottlenecks with $T_R < 30$. This indicates that the larger values of <*r*> could be influenced by the heavy tails of the recovery duration T_R. The "critical slowing down" effect[23] becomes more significant for those bottlenecks with large T_R. This could be due to the fact that nonlinear mechanisms exist between the congestion propagation and dissipation processes given the complex urban traffic network topologies and the travelers' real-time self-adaptive route choice behaviors[23-25].

Supplementary Note 8. Model selection analysis for the distribution of the growth duration.

We conduct the model selection analysis to compare the power-law and the exponential models for growth duration. As shown in Supplementary Table 1, the *p*-values of KS statistic[15] for the estimated models of the power law distribution $p_1(x) \sim x^{-\beta}$ and the exponential distribution $p_2(x) \sim \lambda e^{-\lambda x}$ support that the main part of the growth duration could be well approximated to exponential distributions.

Supplementary Note 9. Discussion of Jaccard index J_B and J_C.

The values of J_C higher than J_B, could be because the congestion sources are stochastic, while the congestion propagation mechanism is fundamental. We clarify that we calculated overlap J_C for congestions associated with all the bottlenecks rather than those bottlenecks with higher overlap J_B. The values of J_C are found higher than J_B, even when the bottlenecks are less similar (Supplementary Fig. 36). For example, for congestion components whose bottlenecks are less similar (e.g., $S_L = 10$, $J_B < 0.1$), similarity J_C of the associated congestions is still slightly larger than J_B of the bottlenecks. This may be because the spatial and temporal congestion propagation have a fundamental pattern[26]. Thus, the overall congestion regions could include similar propagation pathways due to the congestion propagation mechanism.

Supplementary Note 10. Relaxation of overlap of bottlenecks among different days.

We also relax the definition of overlap, and regard the bottlenecks on different days as overlap either if they emerge on the same road, or if one is on the nearest upstream or downstream road segment of another (see the diagrams in Supplementary Fig. 33b). The Jaccard indicator J_B is then defined as the ratio between the size of the "relaxed overlap" and the size of the union of the bottlenecks in two different days. It is shown in Supplementary Fig. 37 that the Jaccard values of the relaxed overlap are significantly larger than the strict overlap. Especially, the relaxed J_B is very close to 1 for the jam components with size threshold $S_L = 1$, meaning that almost all streets can be or close to a bottleneck of a jam with size $\geqslant 1$. Nevertheless, the relaxed Jaccard index J_B is interestingly only around 5% in both rush hours (Supplementary Fig. 37a) and non-rush hours (Supplementary Fig. 37b) for large congestion components with size $\geqslant 20$ roads. This indicates that the large congestions are less recurrent even in the relaxed analysis. The low similarity of large congestion events could be due to that the traffic flows are the result of the dynamic self-organization of the game behaviors of numerous travelers[27-29]. In the day-by-day game process, due to risk

aversion, travelers have been found to make adaptive choices and deviate from the congestions in previous days[30-33]. Thus, large congestion events could be less recurrent than expected.

Supplementary Note 11. Share of large congestion and their growth duration.

We have plotted the CCDF of jam size to calculate the share of large congestions, and plotted CCDF of jam duration for the large congestion with size $S_P \geq 20$. In Supplementary Fig. 39, the CCDF of size S_P on Oct. 16 shows that the number of bottlenecks with $S_P \geq 10$ and $S_P \geq 20$ are respectively over 11,000 and 600, which accounts for 1.8% and 0.09% of all the bottlenecks. The fraction of the large congestions is small yet the most dangerous, and should be the most important yet missing control targets due to their potential damages. In Supplementary Fig. 40, the CCDF of jam duration T shows that the large congestions ($S_P \geq 20$) with jam duration $T \geq 15$ minutes, $T \geq 30$ minutes and $T \geq 60$ minutes, respectively account for around 90%, 46% and 17% of all the large congestions.

Supplementary Note 12. Further attention in the prediction.

The V_{T_I} predictor is practically applicable in early identifying the potential large congestion. Firstly, the V_{T_I} predictor within 15 minutes is practically applicable for most of large congestions in the studied cities. As $ccdf_T$ shown in Supplementary Fig. 43, it is suggested that around 90% of heavy bottlenecks ($S_P \geqslant$25) experienced total jam duration $T \geq 15$ minutes. For such heavy bottlenecks, initial growth speed within 15 minutes could be applied to design response strategies that prevent them from developing into the maximal size or reaching the full jam duration.

Secondly, V_{T_I} within 15 minutes can be applied to identifying the most dangerous heavy jams, before they completely form a gridlock area. $ccdf_{T_G}$ in Supplementary Fig. 43 indicates that around 50% of heavy bottlenecks ($S_P \geqslant$ 25), experienced growth duration $T_G \geq 15$ minutes. For these large congestions, V_{T_I} within 15 minutes performs excellently (AUC around 0.96) in identifying them as heavy bottlenecks. Thus, it can help to design coordinated signal control strategy.

Thirdly, we can balance the prediction time and accuracy in practice. For bottlenecks with relatively short growth duration, their growth speed within 5 minutes (AUC around 0.85) or 10 minutes (AUC around 0.9) are also alternative good predictors. When pursuing early warning instead of accuracy, for example, one could use V_5. Furthermore, the predictors V_5, V_{10}, and V_{15} could be comprehensively applied to early identify the heavy bottlenecks before maximal size or full jam duration.

In future, we would also combine more advanced machine learning technologies with our current framework to improve the comprehensive prediction efficiency.

Supplementary Note 13. More discussion of traffic control devices and congestions.

We assume that some of the longer dissipation may be related to traffic control devices, enhanced by the network's propagation mechanisms. On one hand, studies have found that even though the application of traffic control devices can improve traffic operation, some roads may experience worse traffic and sustain longer congestion duration[34-36]. For example, traffic control devices, such as the "red light" traffic signal, may block vehicles and cause higher traffic flow on some roads. On the other hand, congestions may spread to other roads, due to the network's propagation mechanisms from the bottleneck. For example, previous studies have described the congestion propagation process as the percolation procedure[21, 37], and found such a process can accumulate fractional congestions into a global jam[38, 39], so that it may take a long time to dissipate due to the propagation mechanism. These two mechanisms may interact with each other to produce the long dissipation in some roads.

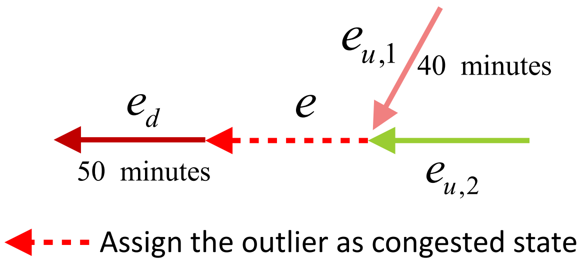

Supplementary Fig. 1. Demonstration of assigning the traffic state for a specific road with an outlier. The arrows of the links are the directions of traffic flow. The road e is assigned as a congested state since its downstream road e_d has been congested 10 minutes earlier than its upstream road $e_{u,1}$. The solid links are the roads with normal velocity records, the dashed links are the roads with an outlier of velocity record. The red links are the congested roads (the darker red means the congestion has lasted for a longer time). The green links are the uncongested roads. The number below the link is the time interval that the congested road has been continuously congested up to now.

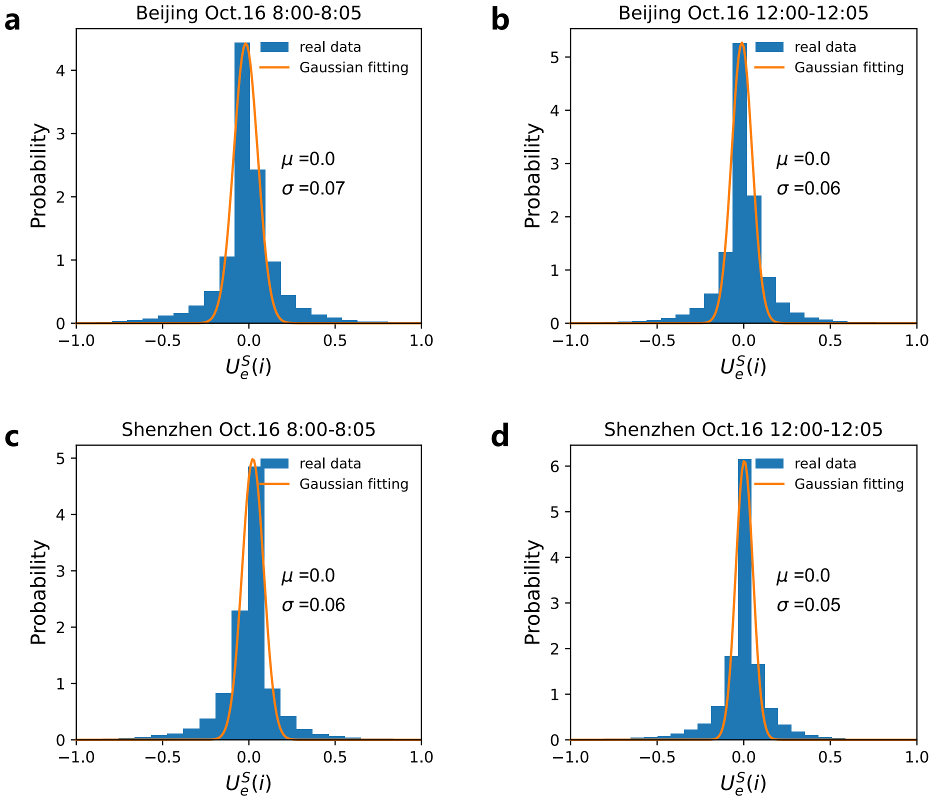

Supplementary Fig. 2. Distributions of rescaled velocity U_e^S over a 5-minute interval in Beijing and Shenzhen. a and **b**. Distributions of the empirical rescaled velocity and the fitted curves of Gaussian model, in (a) rush hours (8:00-8:05) and (b) non-rush hours (12:00-12:05) on Oct. 16, 2015, in Beijing. **c** and **d**. Distributions of the empirical rescaled velocity and the fitted curves of Gaussian distributions, in (c) rush hours (8:00-8:05) and (d) non-rush hours (12:00-12:05) on Oct. 16, 2015, in Shenzhen. In the given 5-minute interval, the distribution includes velocity records in all the roads.

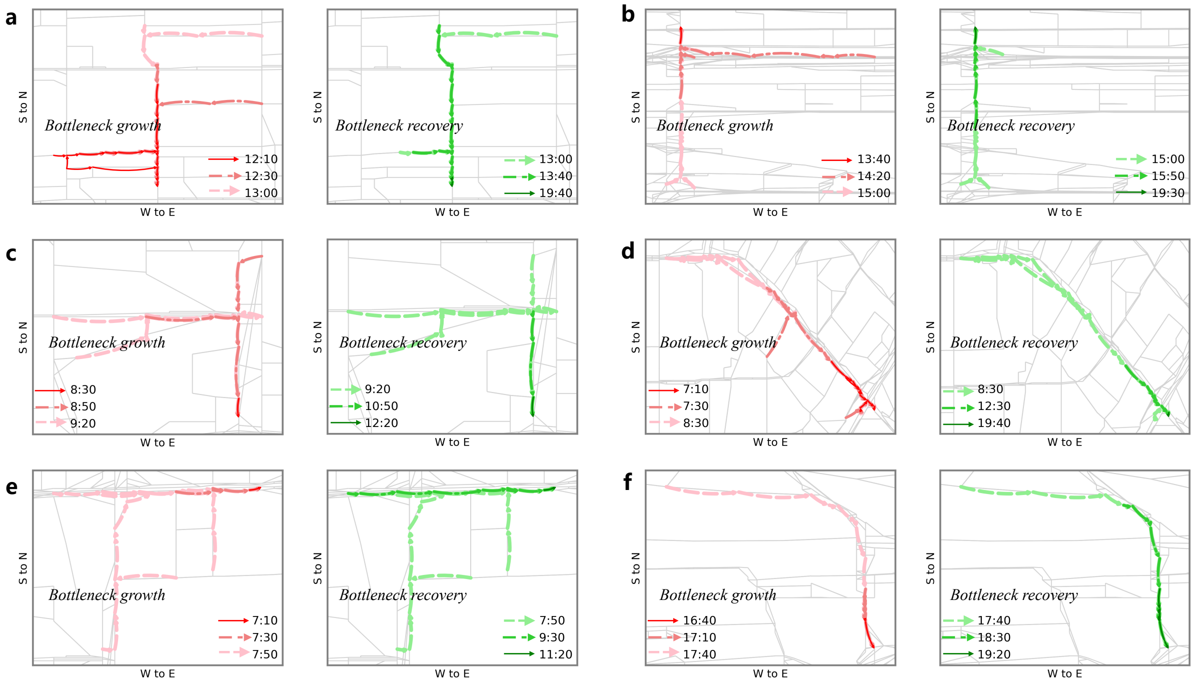

Supplementary Fig. 3. Propagation and dissipation of congestion components associated with six typical bottlenecks, on Monday, October 12, 2015, in Beijing. The arrows of the links are the directions of traffic flow. The congestions originated at the bottleneck (dark red links), developed into more congestions (light red links) in the upstream neighbouring streets during growth stage, and broke down from its maximal size (light green) as the road segments are no longer congested during recovery stage (resolution is 10 minutes here).

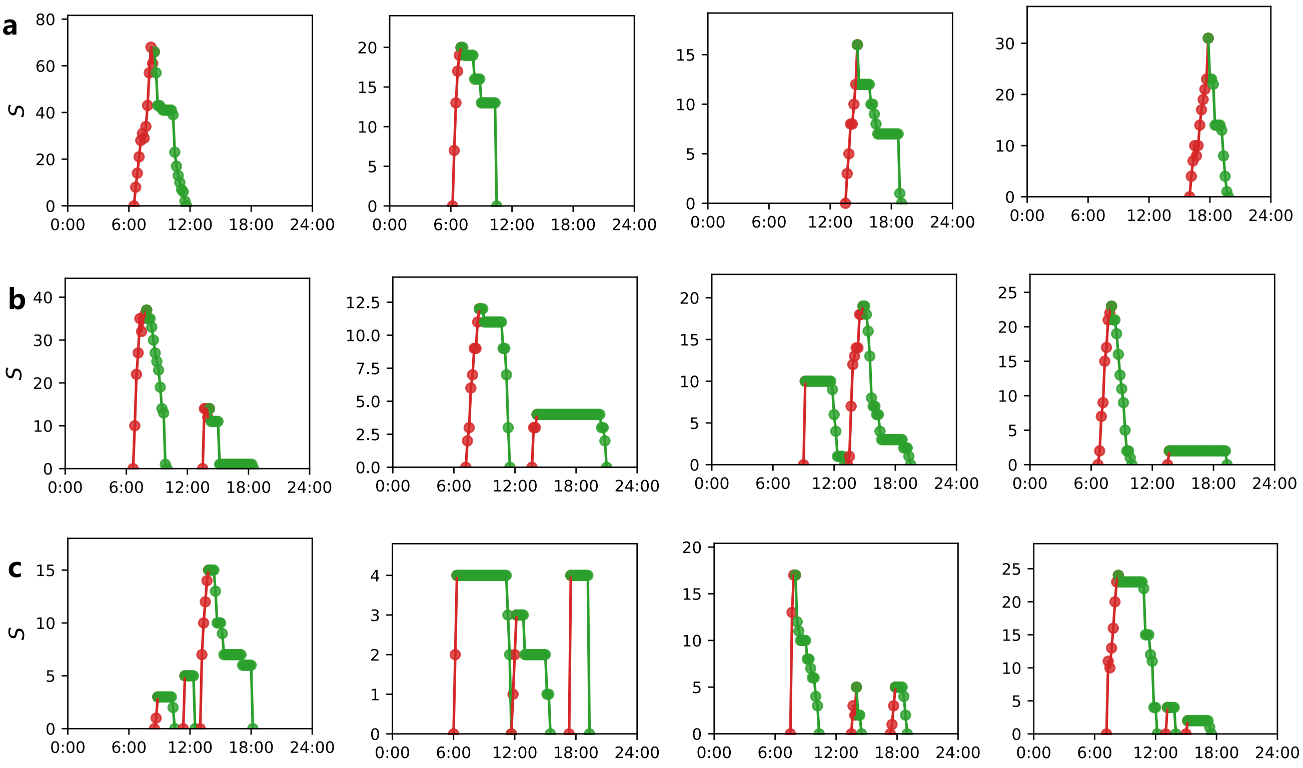

Supplementary Fig. 4. Different evolution patterns of the selected bottlenecks on Monday, October 12, 2015, in Beijing. The size $S(t)$ vs time in different road segments where (**a**) one, (**b**) two or (**c**) three bottlenecks occurred on the same location (resolution is 10 minutes here). The red scatters display the growth stage and the size S of the congestion components increases with time evolving. The green scatters display the recovery stage, and the size S decreases with time evolving.

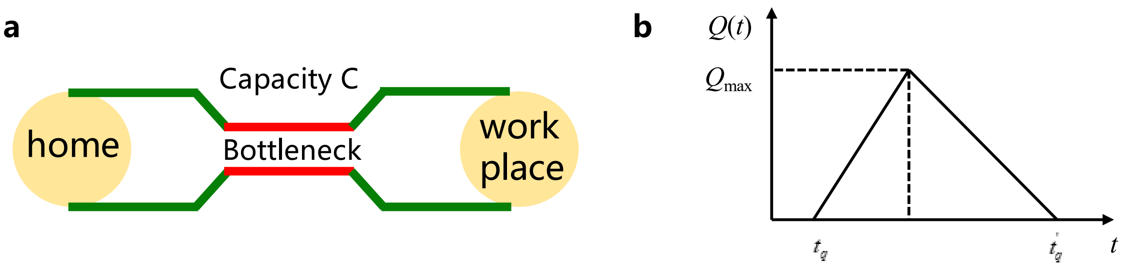

Supplementary Fig. 5. Demonstration of the classical bottleneck model. a. The simplified corridor of the classical bottleneck model. **b**. The number of cars in the queue with time evolving.

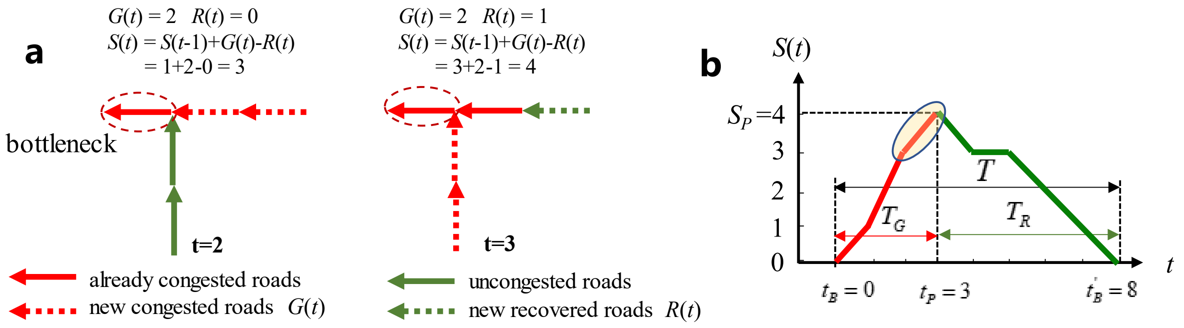

Supplementary Fig. 6. Demonstration of network dynamics of traffic bottleneck. a. Demonstration of congestion size S associated with a bottleneck. It is shown that the number of congested links becomes from $S(t=2)=3$ at time t=2, to $S(t=3)=4$ at time t=3 by integrating the difference between the number of newly developed links $G(t=3)=2$ and newly recovered links $R(t=3)=1$. **b**. Demonstration of the congestion size dynamics. The emergence time $t_B=0$, and the disappearing time $t_B^{'}=8$ are identified by $S(t)=0$. The growth stage ends and the recovery stage starts at time $t_P=3$, distinguished by the maximal size S_P=4. The duration of overall congestion is 8 time intervals.

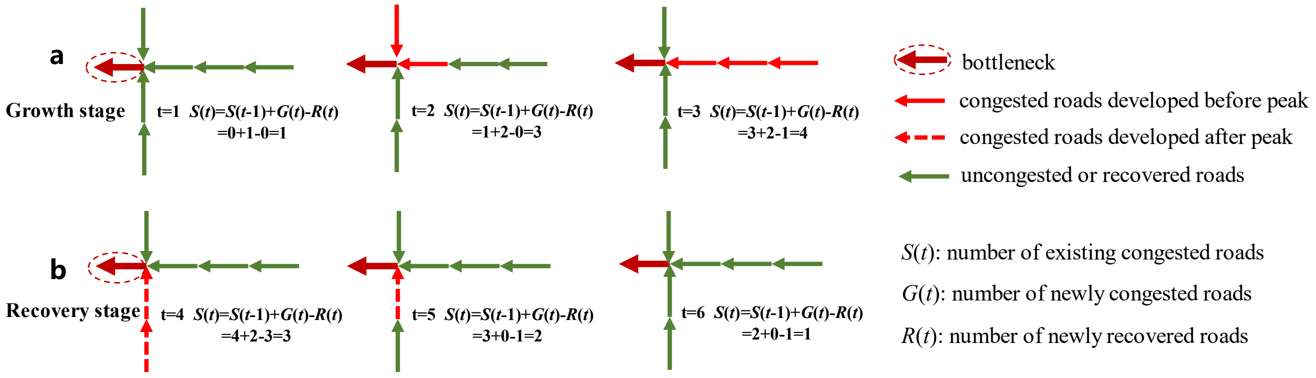

Supplementary Fig. 7. Demonstration of congestion size S associated with a bottleneck in (a) the growth stage and (b) the recovery stage.

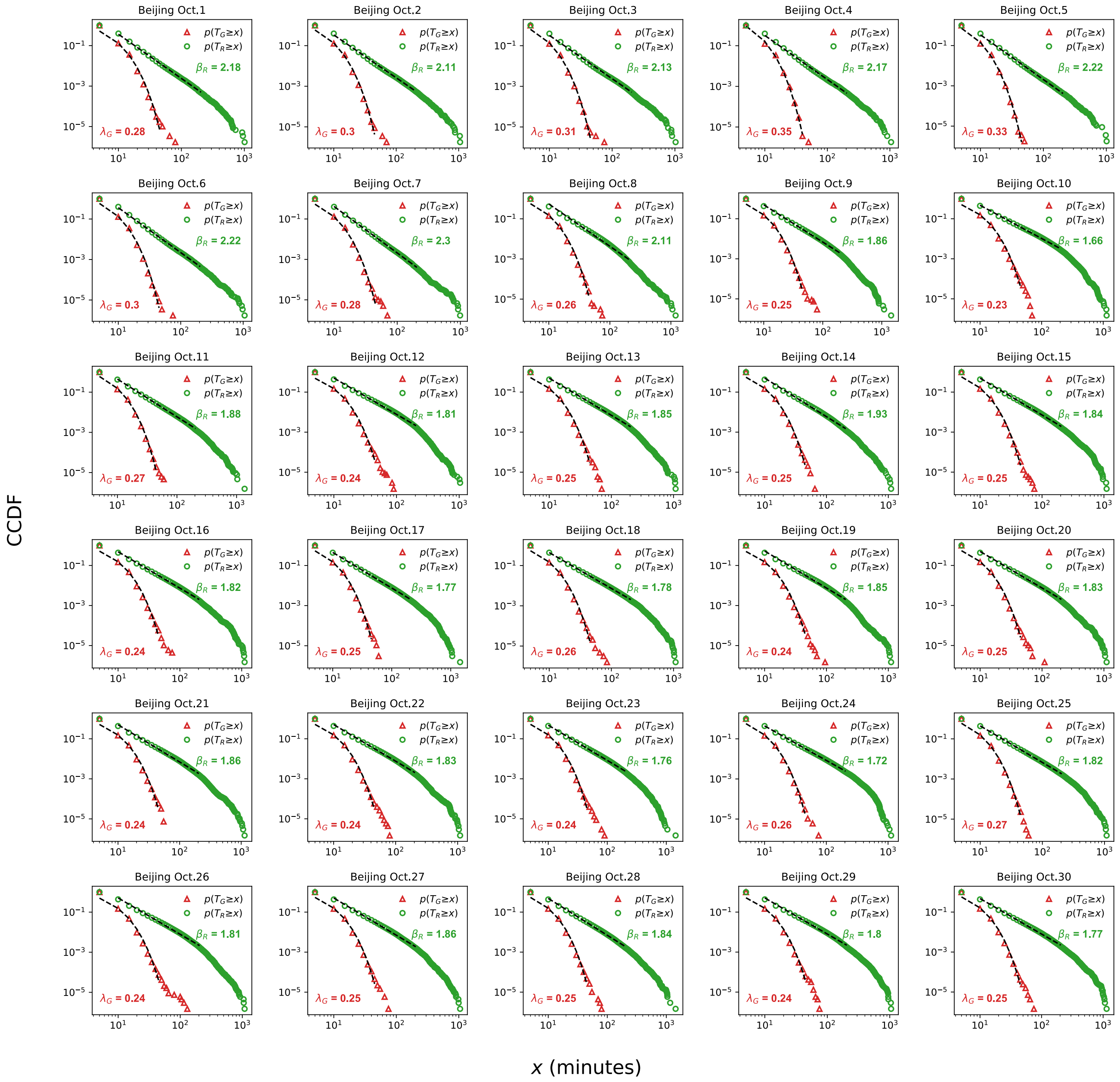

Supplementary Fig. 8. The CCDFs of the growth duration T_G and the recovery duration T_R on 30 days of October 2015 in Beijing. The CCDF of growth duration T_G is well approximated by an exponential distribution. The CCDF of recovery duration T_R is well approximated by a power law distribution. The distributions and exponents are similar for the same type of days. October 1 to October 7 are seven holidays of China's National Day, Oct. 11, 17, 18, 24, and 25 are the regular weekends, and the other 18 days are the workdays.

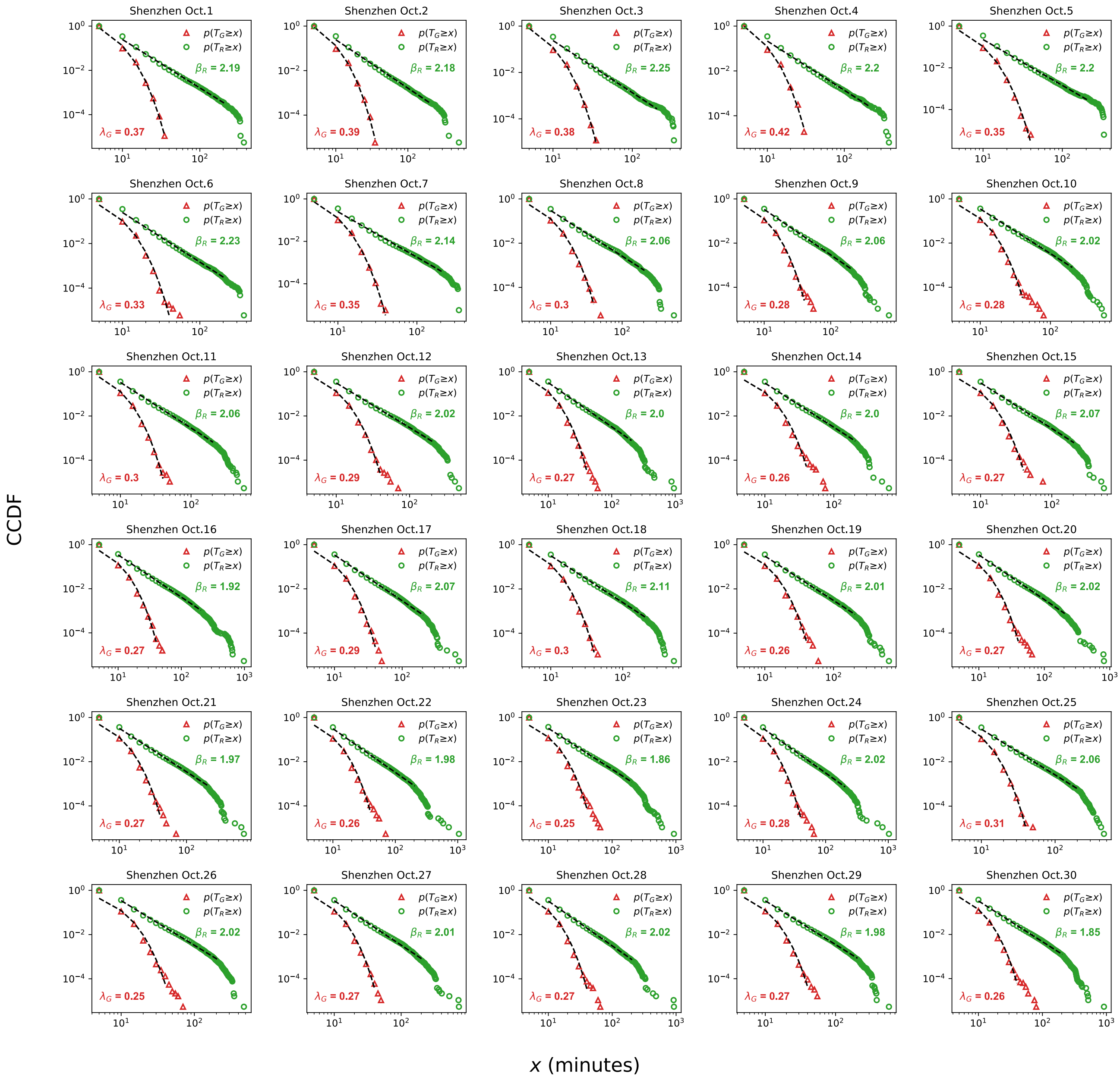

Supplementary Fig. 9. The CCDFs of the growth duration T_G and the recovery duration T_R on 30 days of October 2015 in Shenzhen. The CCDF of growth duration T_G is well approximated by an exponential distribution. The CCDF of the recovery duration T_R is well approximated by a power law distribution. The distributions and exponents are similar for the same type of days. October 1 to October 7 are seven holidays of China's National Day, Oct. 11, 17, 18, 24, and 25 are the regular weekends, and the other 18 days are the workdays.

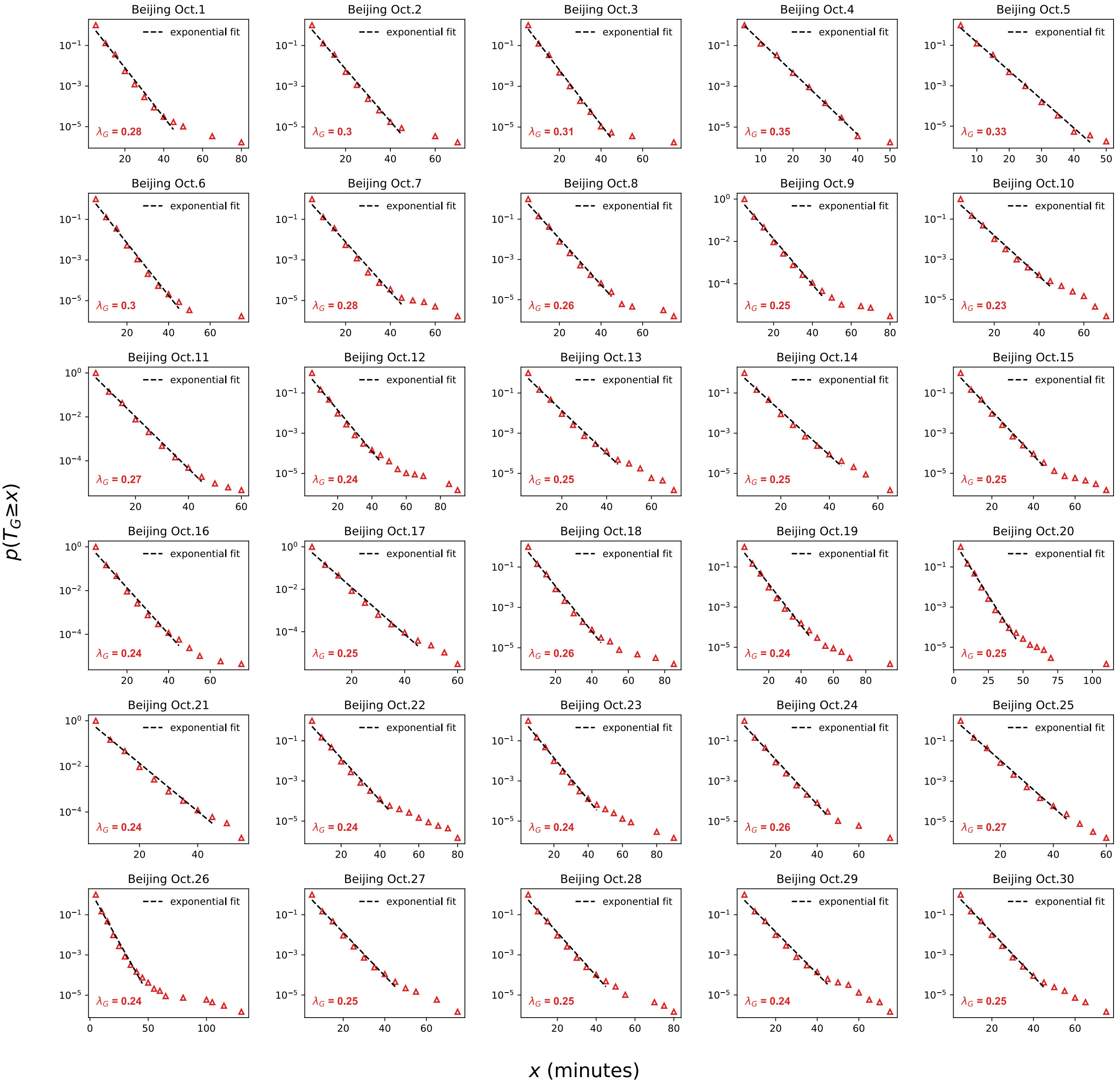

Supplementary Fig. 10. The CCDF of the growth duration T_G in log-linear plots for 30 days of October 2015 in Beijing. The main parts of the CCDF of growth duration are well fitted by exponential distribution. The tail of the CCDF seems to be a little fatter than the exponential distribution. The exponential distributions and their exponents λ are similar for the same type of days. October 1 to October 7 are seven holidays of China's National Day, Oct. 11, 17, 18, 24, and 25 are the regular weekends, and the other 18 days are the workdays.

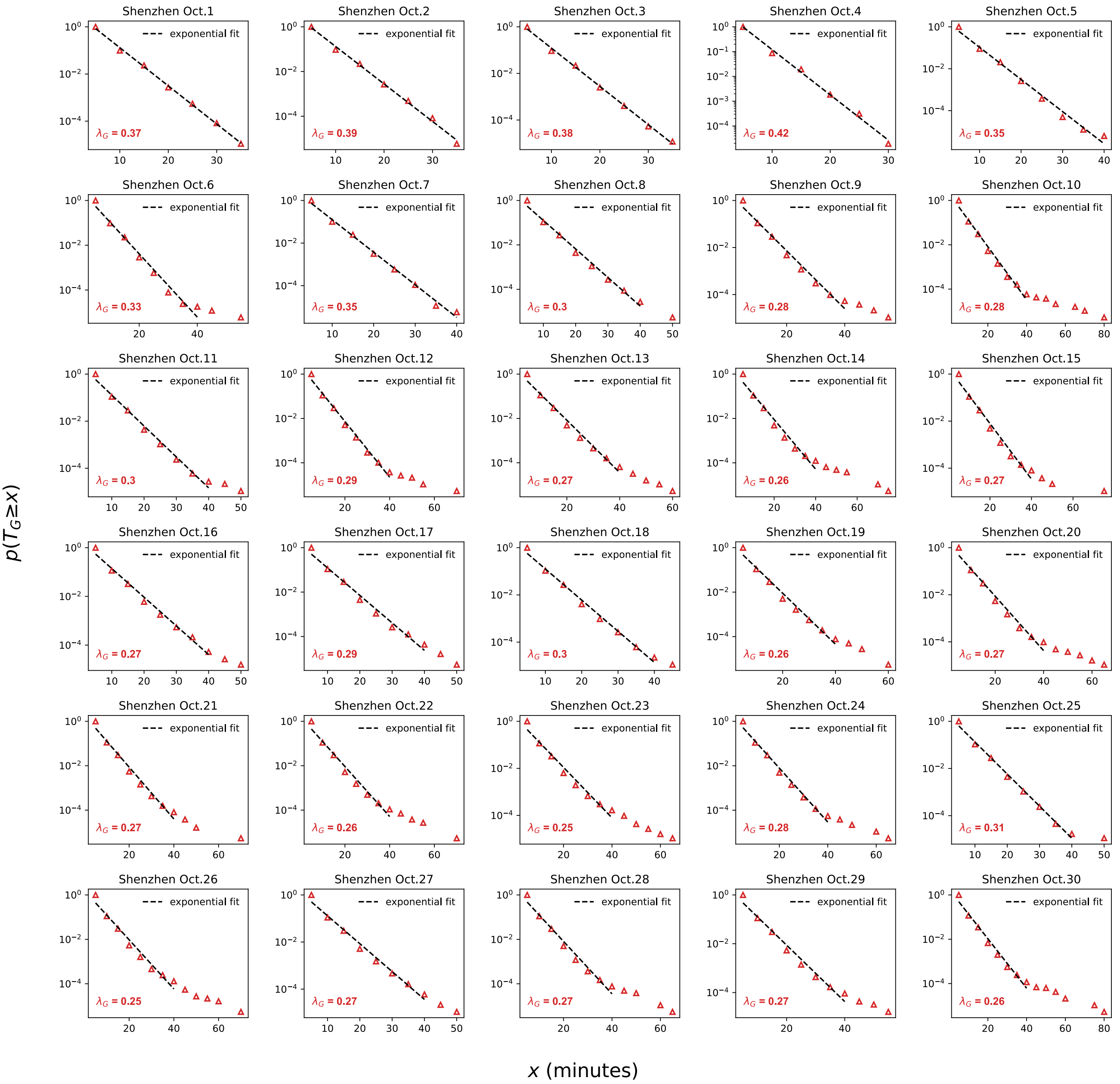

Supplementary Fig. 11. The CCDF of the growth duration T_G in log-linear plots for 30 days of October 2015 in Shenzhen. The main parts of the CCDF of growth duration are well fitted by exponential distribution. The tail of the CCDF seems to be a little fatter than the exponential distribution. The exponential distributions and their exponents λ are similar for the same type of days. October 1 to October 7 are seven holidays of China's National Day, Oct. 11, 17, 18, 24, and 25 are the regular weekends, and the other 18 days are the workdays.

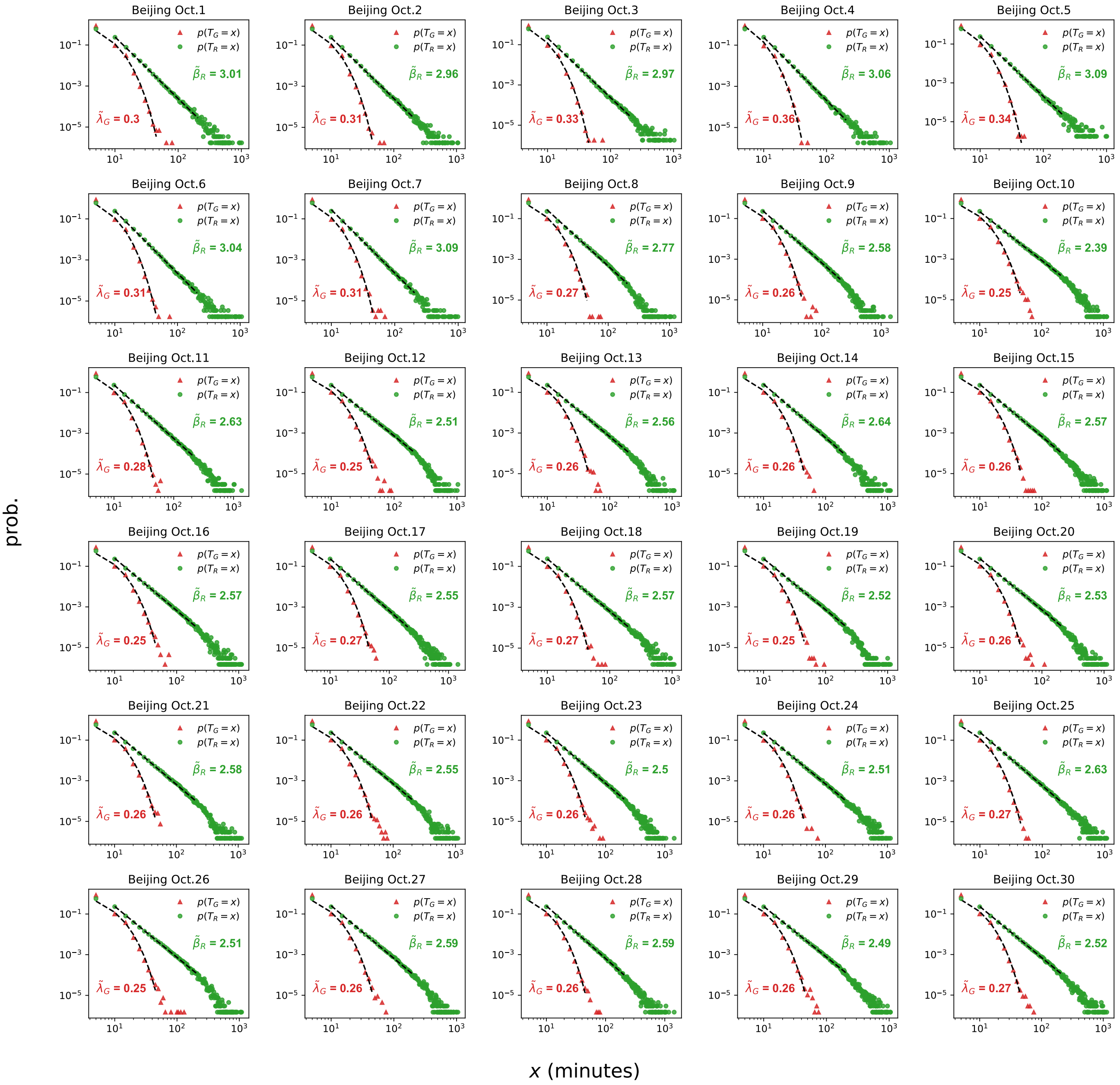

Supplementary Fig. 12. Distributions of growth duration T_G and recovery duration T_R on 30 days of October 2015 in Beijing. The y-axis is the probability of the growth duration (red) and the recovery duration (green). The distribution of growth duration T_G is approximated by an exponential distribution $p(T_G = x) \sim \tilde{\lambda}_G e^{-\tilde{\lambda}_G x}$. The distribution of recovery duration T_R is approximated by a power law distribution $p(T_R = x) \sim x^{-\tilde{\beta}_R}$. The distributions and exponents are similar for the same type of days. October 1 to October 7 are seven holidays of China's National Day, Oct. 11, 17, 18, 24, and 25 are the regular weekends, and the other 18 days are the workdays.

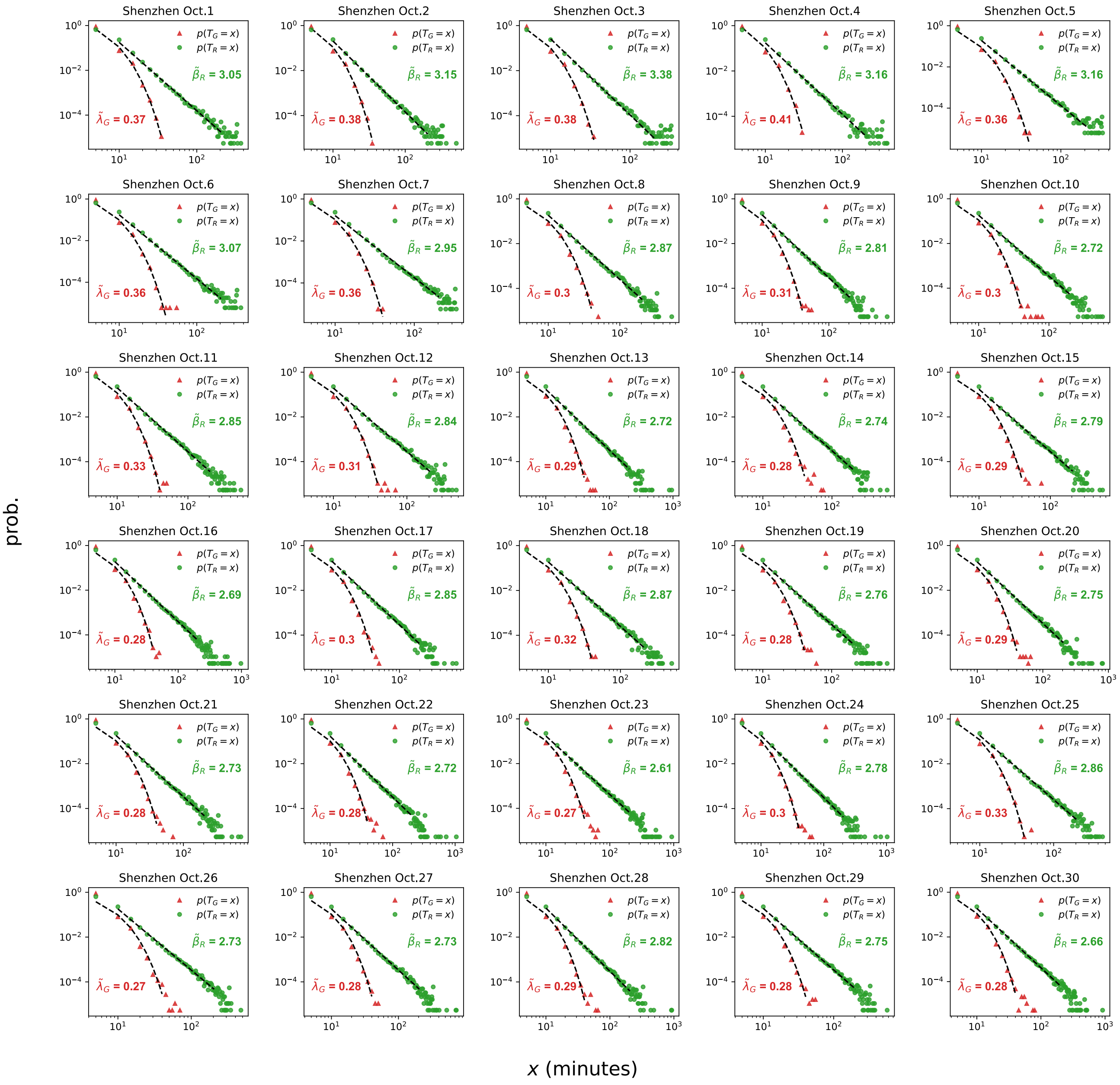

Supplementary Fig. 13. Distributions of growth duration T_G and recovery duration T_R on 30 days of October 2015 in Shenzhen. The y-axis is the probability of the growth duration (red) and the recovery duration (green). The distribution of growth duration T_G is approximated by an exponential distribution $p(T_G = x) \sim \tilde{\lambda}_G e^{-\tilde{\lambda}_G x}$. The distribution of recovery duration T_R is approximated by a power law distribution $p(T_R = x) \sim x^{-\tilde{\beta}_R}$. The distributions and exponents are similar for the same type of days. October 1 to October 7 are seven holidays of China's National Day, Oct. 11, 17, 18, 24, and 25 are the regular weekends, and the other 18 days are the workdays.

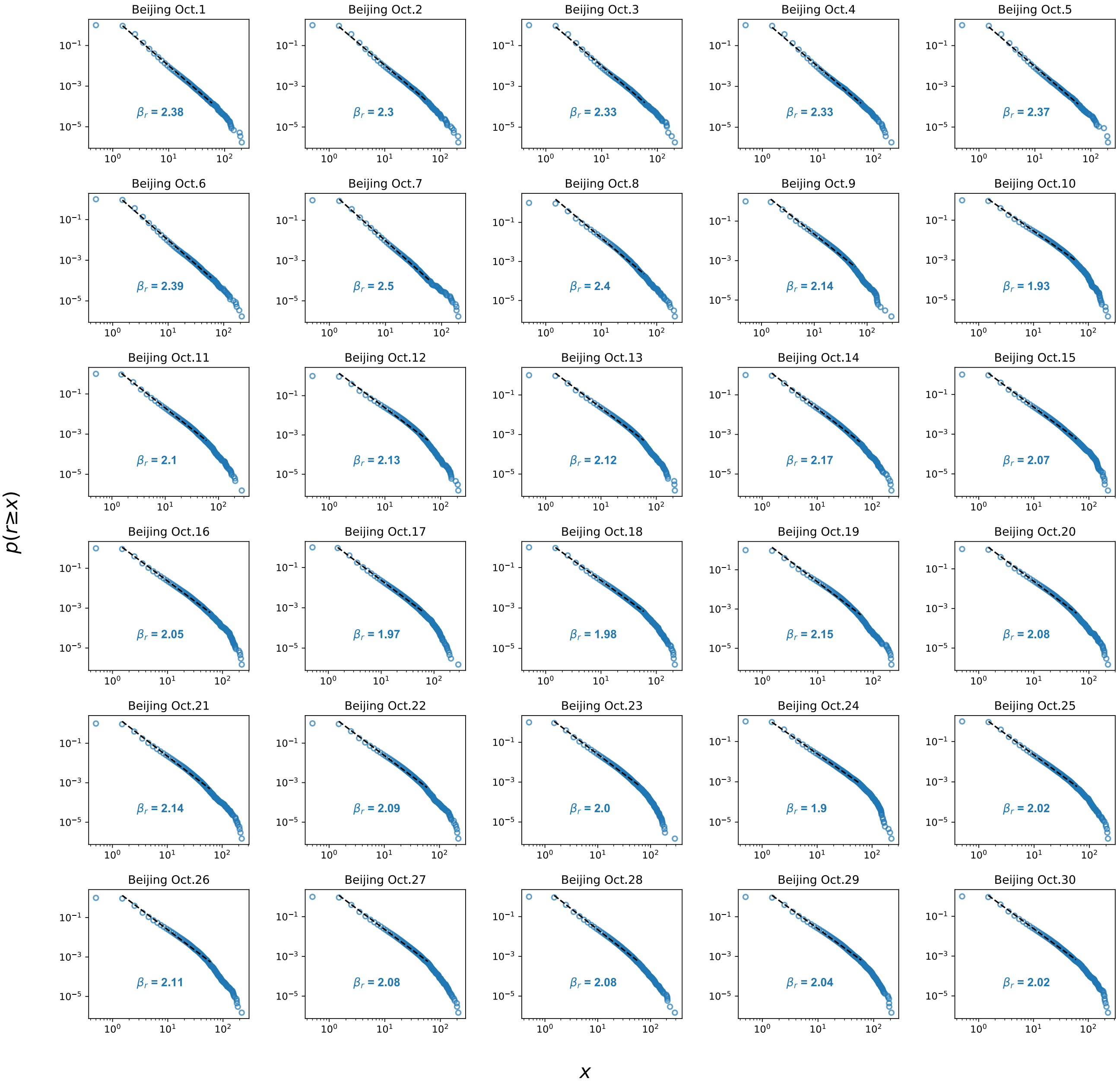

Supplementary Fig. 14. The CCDF of ratio r between recovery duration T_R and growth duration T_G on 30 days of October 2015 in Beijing. Ratio r between recovery duration and growth duration approximately follows similar power-law patterns $p(r \geq x) \sim x^{-\beta_r}$ in different days.

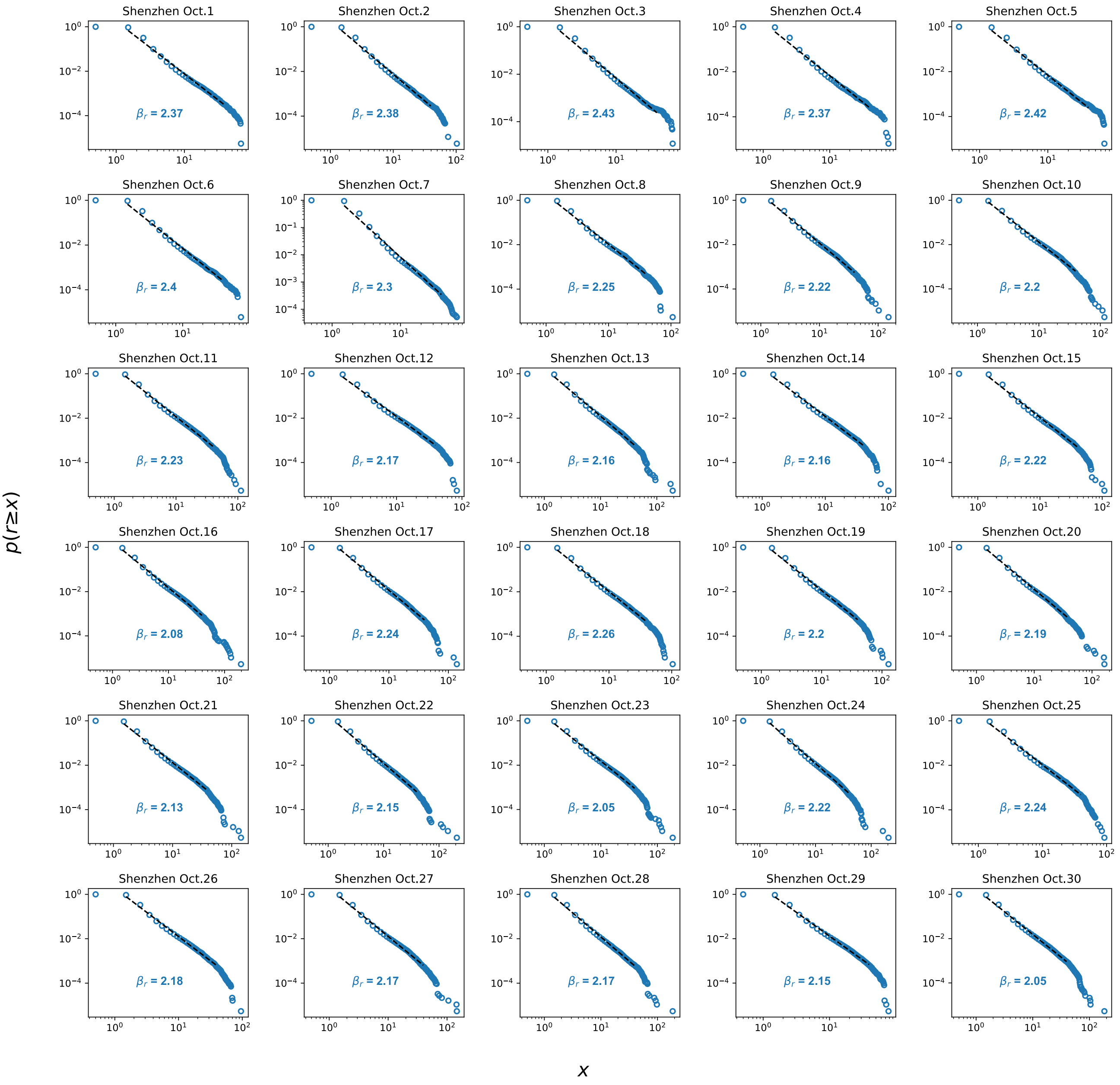

Supplementary Fig. 15. The CCDF of ratio r between recovery duration T_R and growth duration T_G on 30 days of October 2015 in Shenzhen. Ratio r between recovery duration and growth duration approximately follows similar power-law patterns $p(r \geq x) \sim x^{-\beta_r}$ in different days.

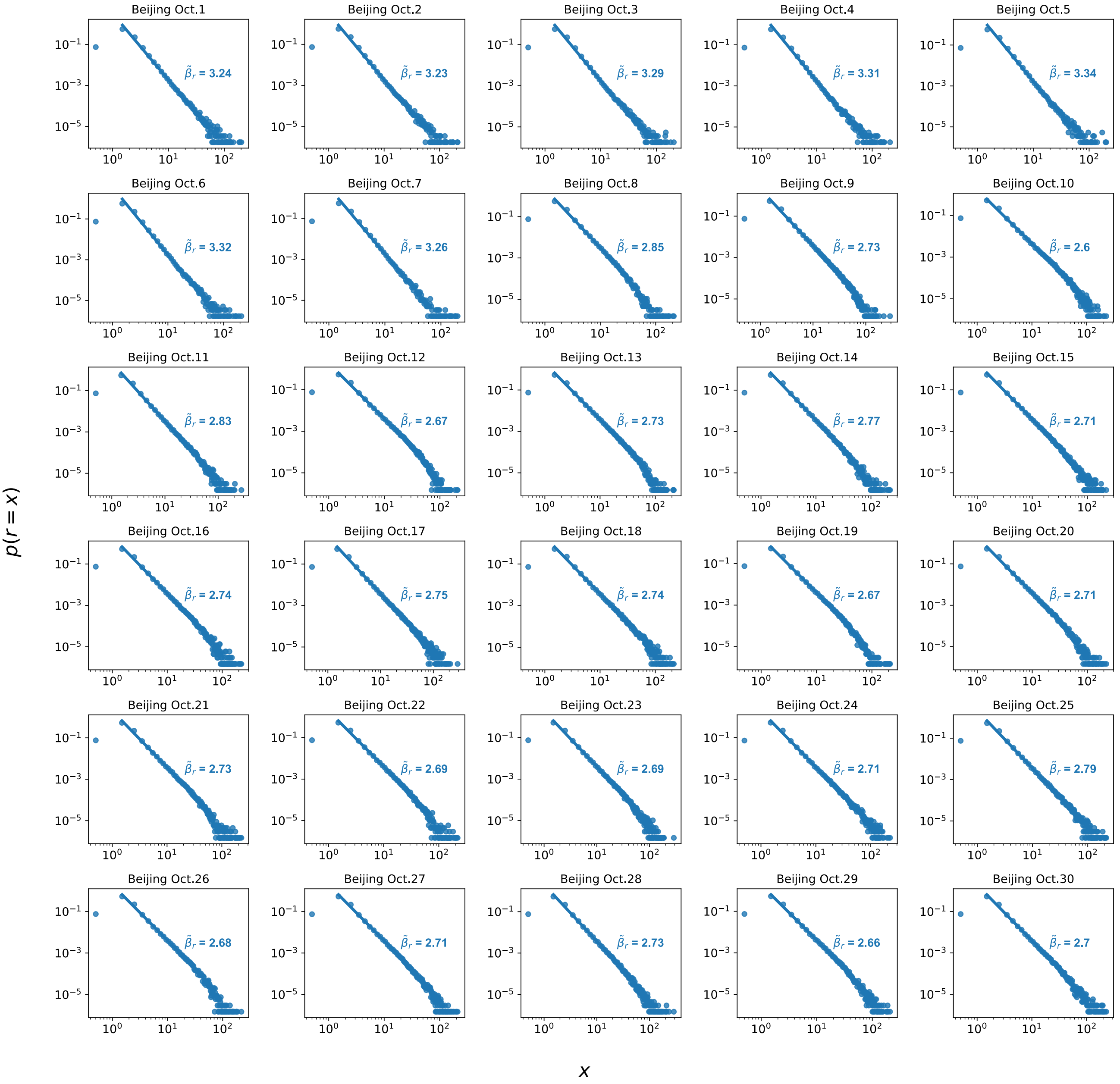

Supplementary Fig. 16. Distribution of ratio r between recovery duration T_R and growth duration T_G on 30 days of October 2015 in Beijing. The y-axis is the probability of the ratio r. The ratio r between recovery duration and growth duration approximately follows similar power-law patterns $p(r=x) \sim x^{-\tilde{\beta}_r}$ in different days.

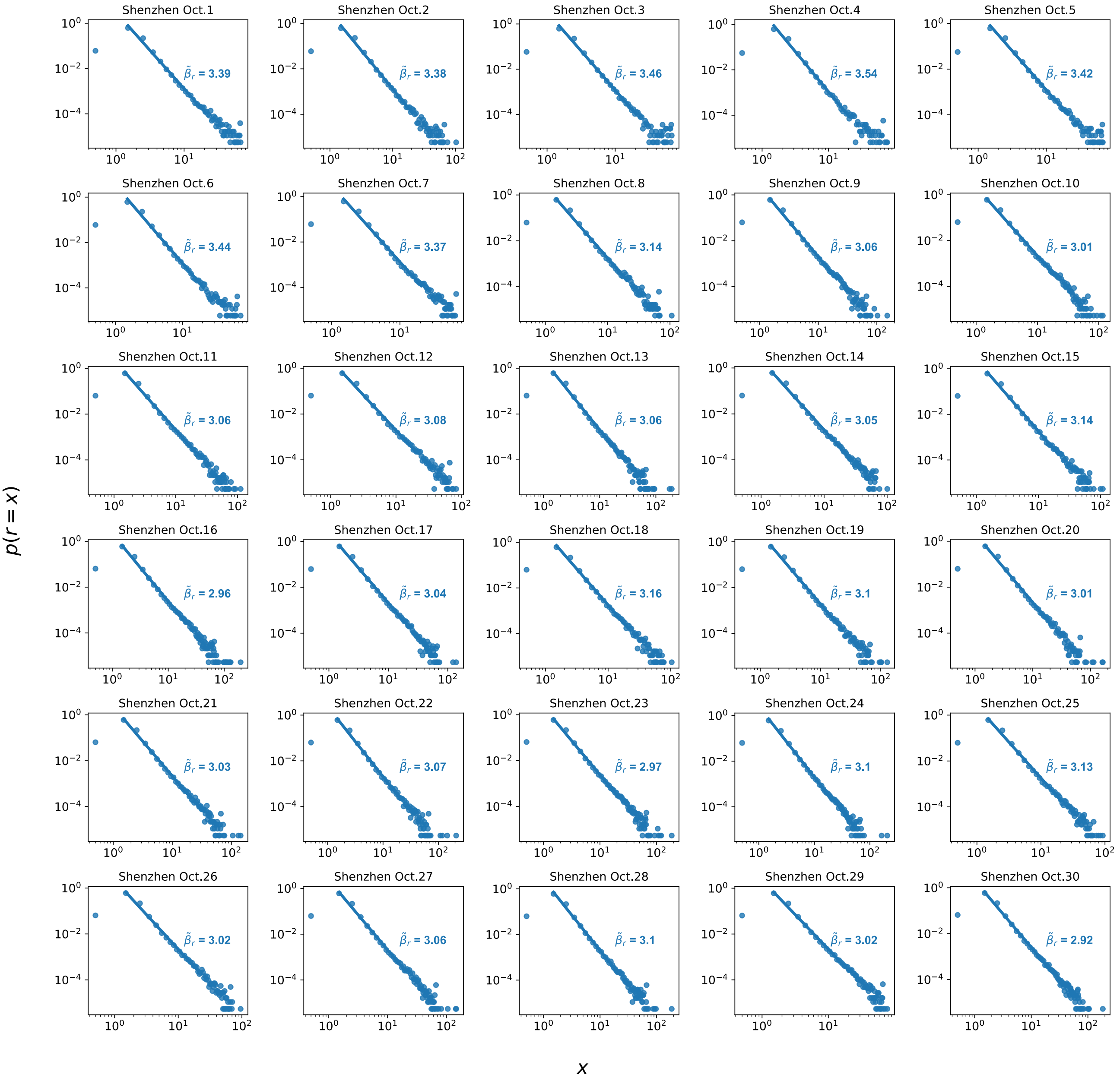

Supplementary Fig. 17. Distribution of ratio r between recovery duration T_R and growth duration T_G on 30 days of October 2015 in Shenzhen. The y-axis is the probability of the ratio r. The ratio r between recovery duration and growth duration approximately follows similar power-law patterns $p(r = x) \sim x^{-\tilde{\beta}_r}$ in different days.

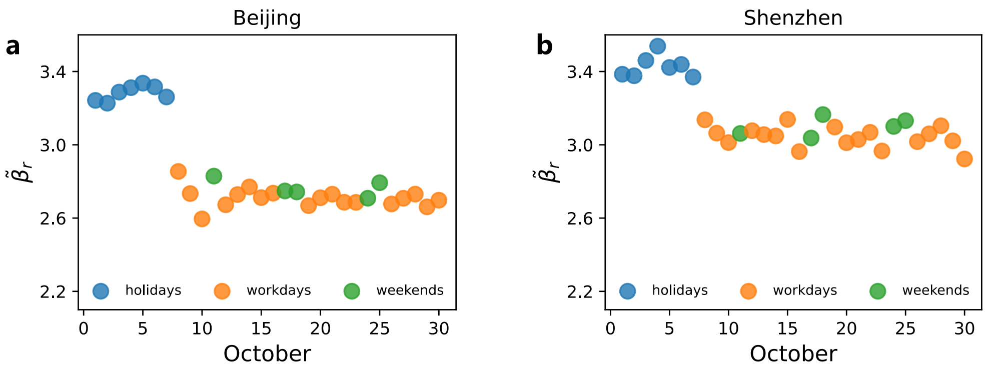

Supplementary Fig. 18. Exponents $\tilde{\beta}_r$ on 30 days of October, 2015 in (a) Beijing and (b) Shenzhen. The exponents in two cities indicate that the congestion components during the workdays take a much longer time to recover than growth compared with holidays.

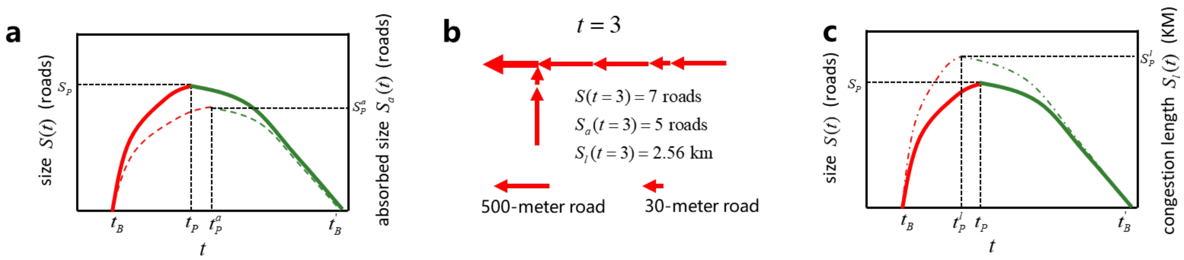

Supplementary Fig. 19. Demos of the absorbed congestion size $S_a(t)$, congestion length $S_l(t)$, and full size $S(t)$. a and **c.** Demos of dynamics of (a) the absorbed size $S_a(t)$ and (c) the congestion length $S_l(t)$ compared to the full size $S(t)$. **b**. Three types of size S of the congestions associated with the given bottleneck at the selected time t=3. The congestion component has totally 7 road segments, including five 500-meter roads and two 30-meter roads.

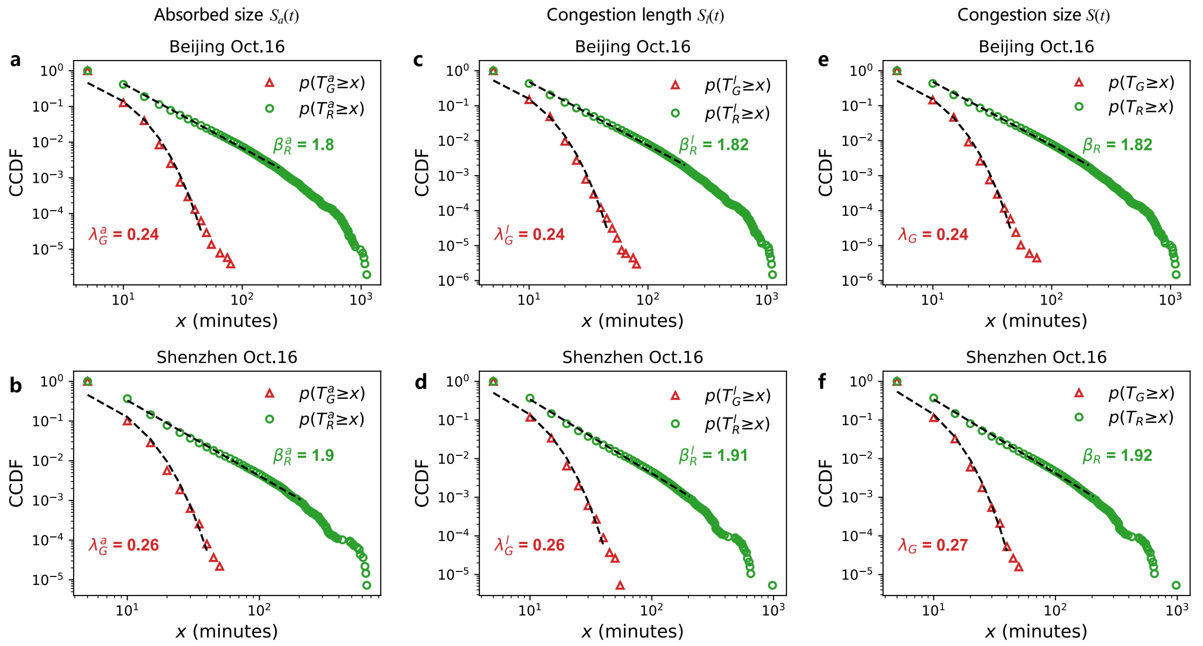

Supplementary Fig. 20. The CCDFs of growth duration (red triangles) and recovery duration (green circles) obtained by three definitions of congestion size. a and **b**. The CCDFs of the growth duration T_G^a and the recovery duration T_R^a identified based on the absorbed size $S_a(t)$ on October 16, Friday, 2015, for (a) Beijing and (b) Shenzhen. **c** and **d**. The CCDFs of the growth duration T_G^l and the recovery duration T_R^l identified based on the congestion length $S_l(t)$ on October 16, Friday, 2015, for (c) Beijing and (d) Shenzhen. **e** and **f**. The CCDFs of the growth duration T_G and the recovery duration T_R identified based on the full size $S(t)$, on October 16, Friday, 2015, for (e) Beijing and (f) Shenzhen.

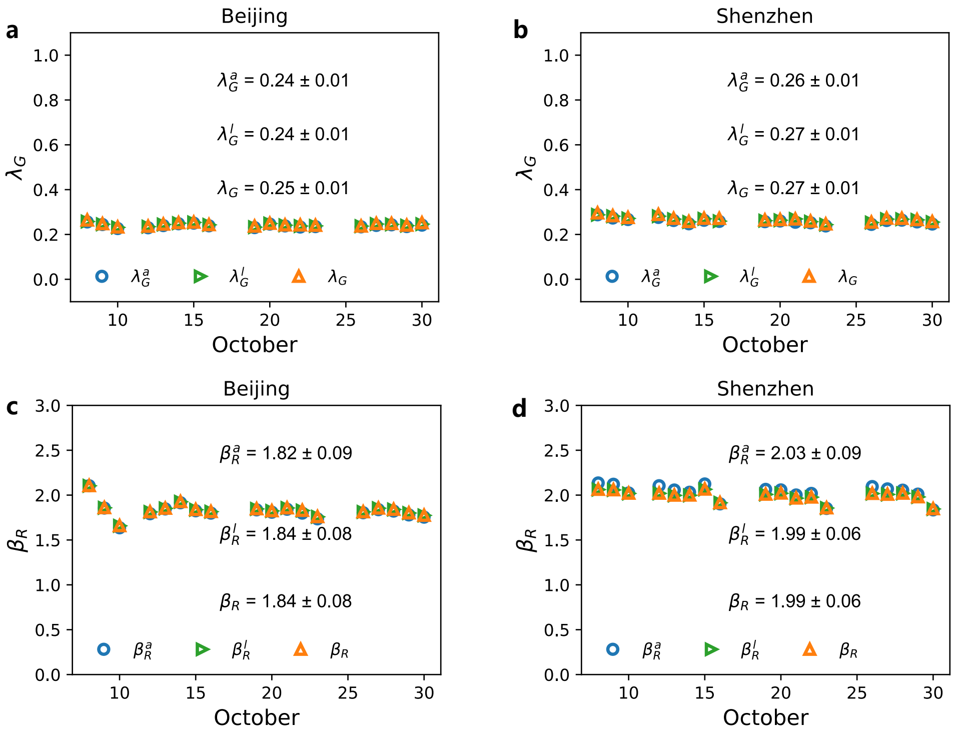

Supplementary Fig. 21. Exponents for CCDFs of the growth duration and the recovery duration obtained by three definitions of congestion size, on 18 workdays in October, 2015. a and **b.** Exponents λ_G^a, λ_G^l, and λ_G for the exponential distributions of CCDF of the growth duration respectively obtained by the dynamics of absorbed size $S_a(t)$, congestion length $S_l(t)$ and size $S(t)$, for 18 workdays in (a) Beijing and (b) Shenzhen. **c** and **d.** Exponents β_R^a, β_R^l, and β_R for the power-law distributions of CCDF of the recovery duration respectively obtained by the dynamics of absorbed size $S_a(t)$, congestion length $S_l(t)$ and size $S(t)$, for 18 workdays in (c) Beijing and (d) Shenzhen.

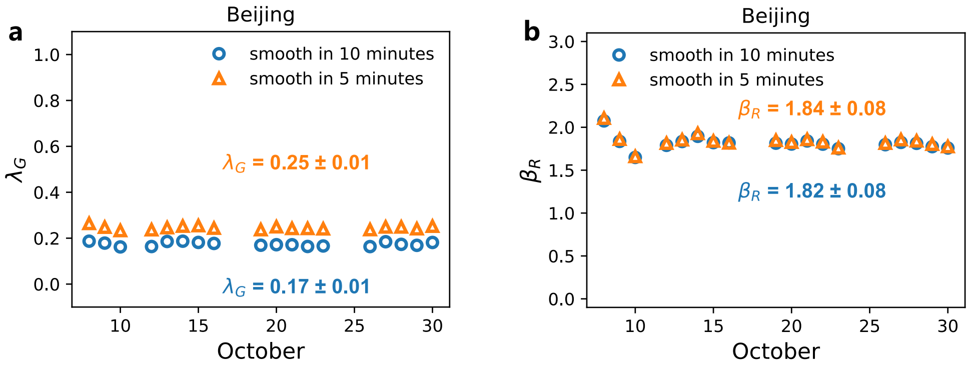

Supplementary Fig. 22. Exponents of (a) λ_G fitted by exponential distribution for the CCDF of the growth duration, and (b) β_R fitted by power-law distributions for the CCDF of the recovery duration, respectively by smoothing velocity records in 10 minutes and 5 minutes, in 18 workdays of Beijing. It is shown that power-law exponents β_R for recovery duration are robust in

two resolutions. The exponential exponent λ_G for growth duration becomes a bit smaller when smoothing in 10 minutes. This is because the evolution stages of some bottlenecks are merged to relate with a single bottleneck when smoothing in 10 minutes. For example, on Oct. 16, in Beijing, the number of bottlenecks is merged and decreased to over 390,000 by smoothing in 10 minutes, accounting for 58% of the bottlenecks (over 670,000) when smoothing every 5 minutes.

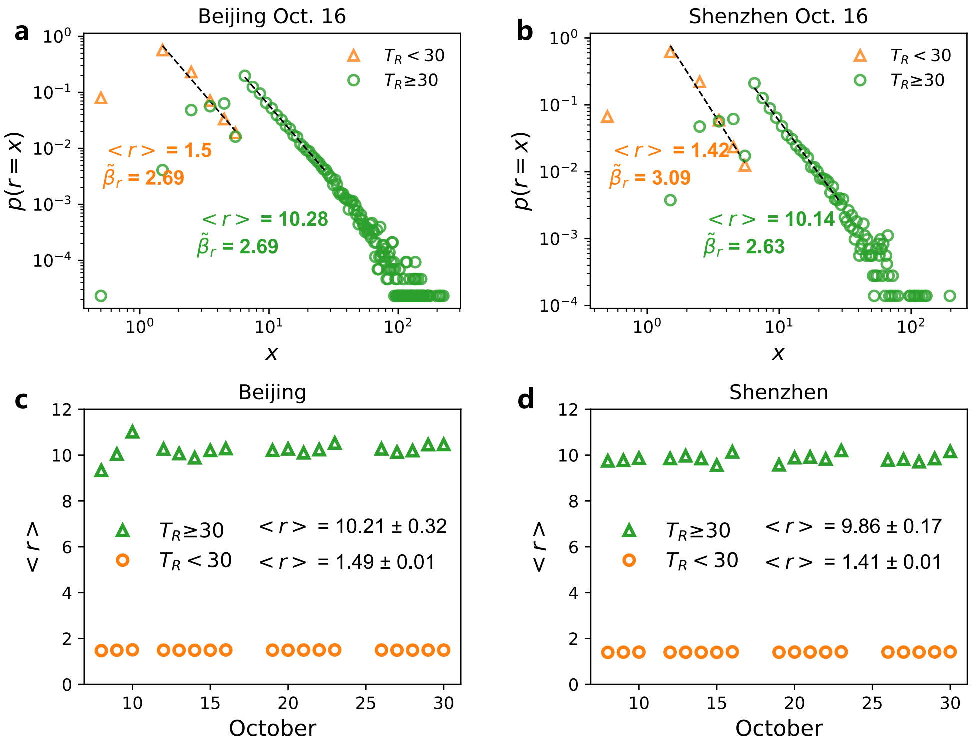

Supplementary Fig. 23. Values of mean ratio <*r*> between recovery duration T_R and growth duration T_G for bottlenecks with different T_R. a and **b.** Distributions of ratio *r* between recovery duration and growth duration respectively for bottlenecks with recovery duration $T_R \geq 30$ minutes and $T_R < 30$ minutes. **c** and **d.** Mean value <*r*> for two groups of bottlenecks on 18 workdays in Oct. 2015, of (c) Beijing and (d) Shenzhen.

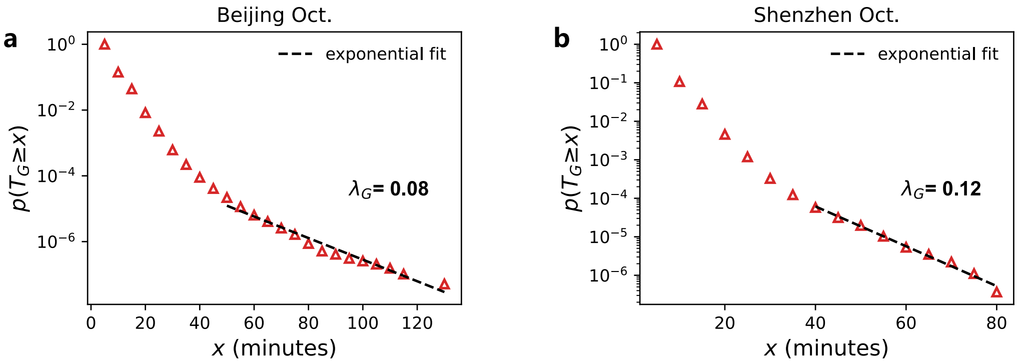

Supplementary Fig. 24. CCDF of growth duration in the tail by accumulating the data of 30 days in October 2015 together. **a** shows the exponential fitting of CCDF of growth duration above 50 minutes in Beijing and **b** shows the exponential fitting of CCDF of growth duration above 40 minutes in Shenzhen. Note that we find a suitable crossover time is 50 minutes in Beijing and 40 minutes in Shenzhen. By the suitable crossover time here, both regimes are well-fitted.

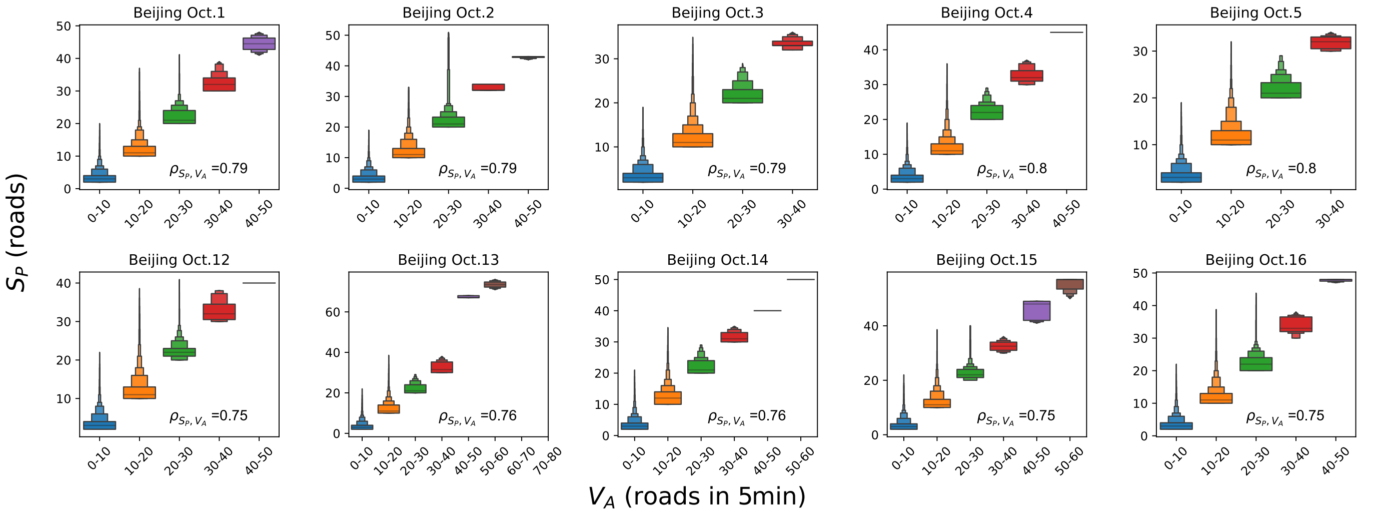

Supplementary Fig. 25. Box plots of maximal size S_P grouped by different growth speed V_A of ten days in October 2015, Beijing. The figures at the top and bottom are the results for five holidays and five workdays, respectively. Here, V_A means the average number of the increased congested road segments in every 5 minutes during the full growth stage. The outliers are not displayed in the box plots. The results indicate that similar correlation patterns between spatial size S_P and growth speed V_A exist on different days. Bottlenecks with $S_P \geq 2$ are counted here.

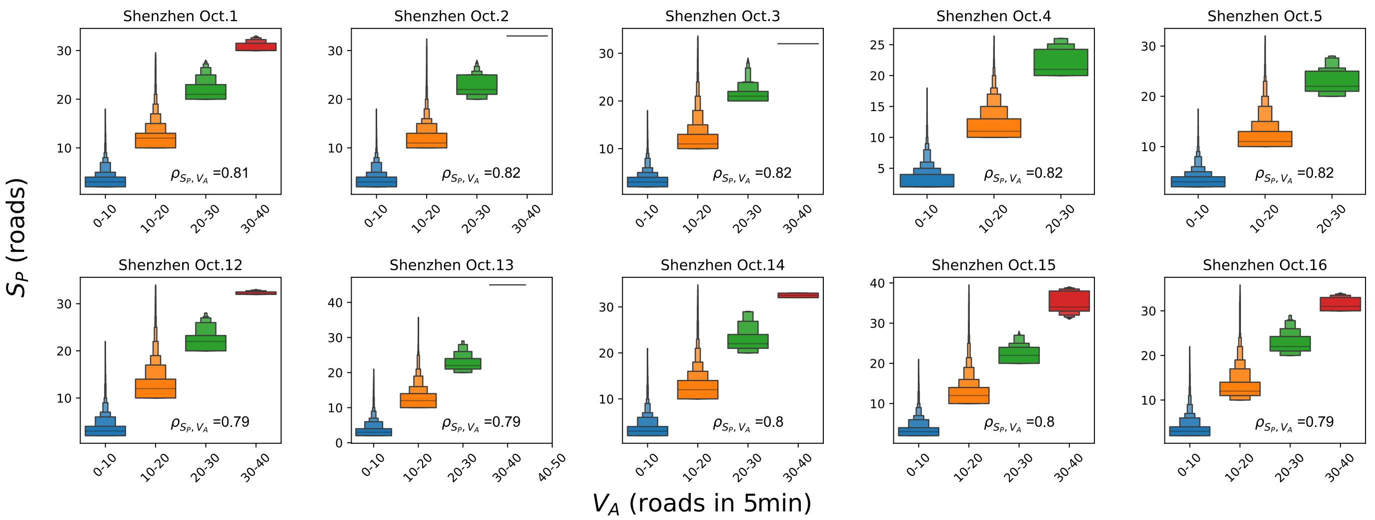

Supplementary Fig. 26. Box plots of maximal size S_P grouped by different growth speed V_A of ten days in October 2015, Shenzhen. The figures at the top and bottom are the results for five holidays and five workdays, respectively. Here, V_A means the average number of the increased congested road segments in every 5 minutes during the full growth stage. The outliers are not displayed in the box plots. The results indicate that similar correlation patterns between spatial size S_P and growth speed V_A exist on different days. Bottlenecks with $S_P \geq 2$ are counted here.

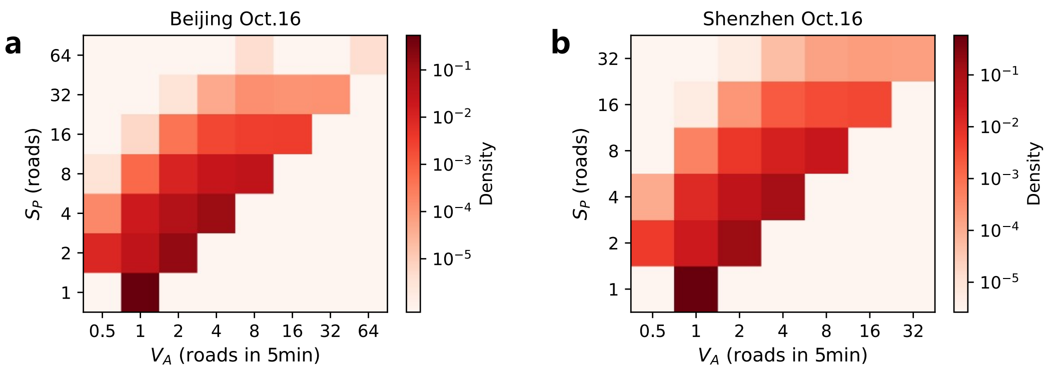

Supplementary Fig. 27. Density surfaces of the average growth speed V_A and the maximal size S_P on Oct. 16, 2015, Friday, for (a) Bejing and (b) Shenzhen. The x-axis is the average growth speed V_A, the y-axis is the maximal size S_P, and the color bar represents the density of the observations. It is shown that the congestion components with a larger average growth speed V_A, are more likely to reach a larger congestion size S_P.

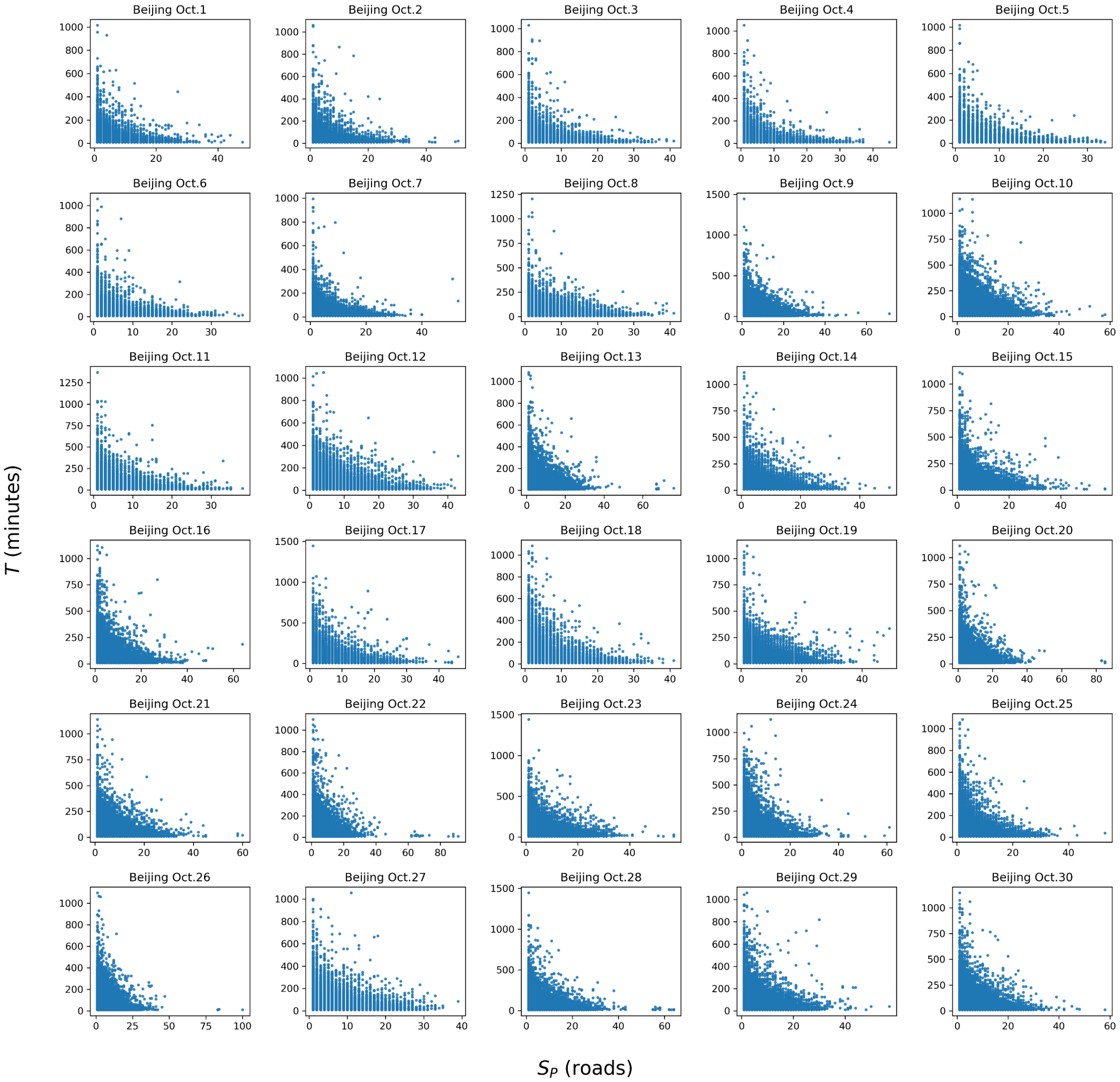

Supplementary Fig. 28. Scatter plots of the maximal size S_P and lifespan T of congestion components in October 2015, Beijing. The figures indicate that the congestion components with a larger size S_P are less probable to sustain for a very long duration. However, large congestions (e.g., $S_P \geq 20$) can still sustain for hours.

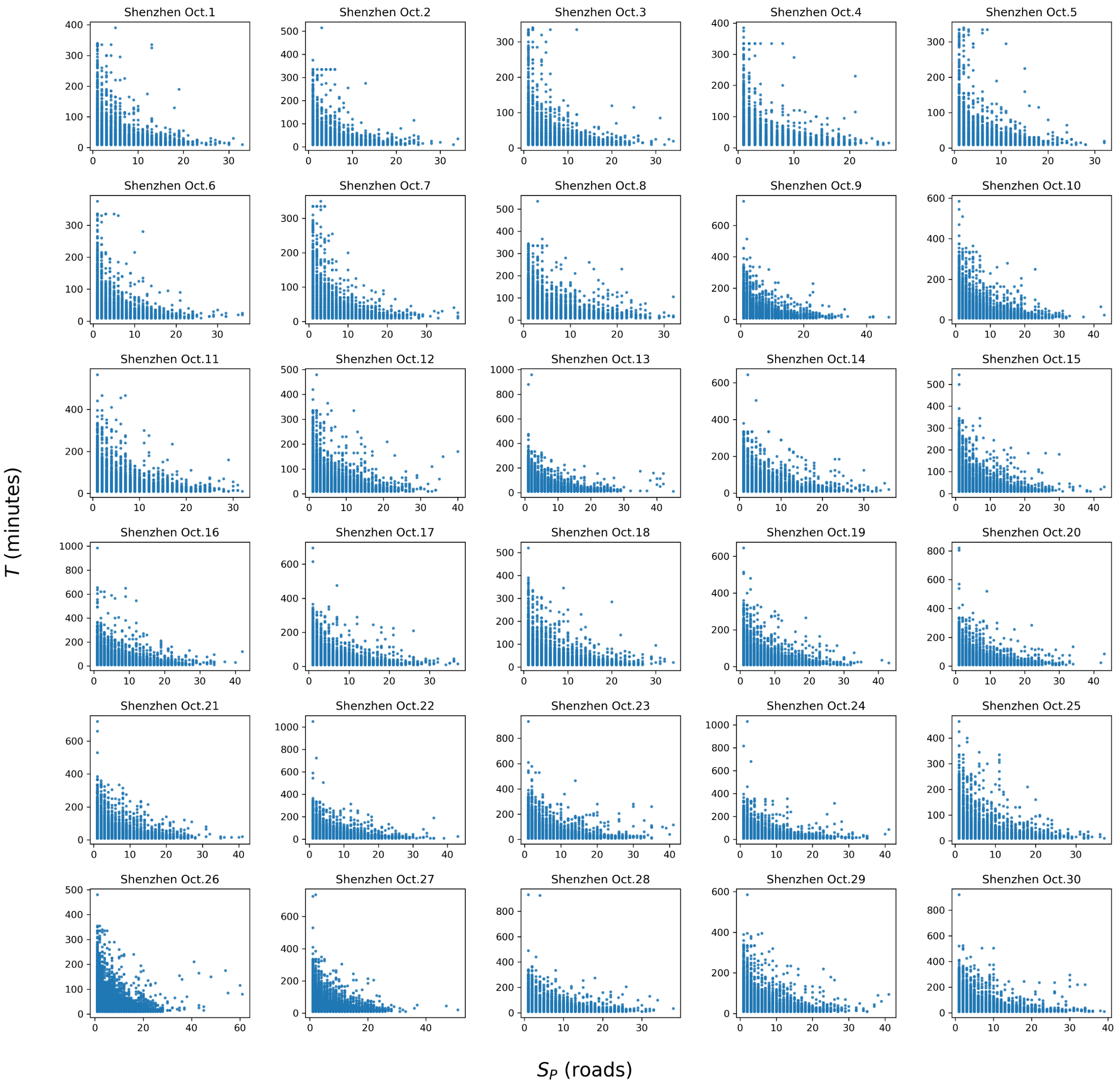

Supplementary Fig. 29. Scatter plots of the maximal size S_P and lifespan T of congestion components in October 2015, Shenzhen. The figures indicate that the congestion components with a larger size S_P are less probable to sustain for a very long duration. However, large congestions (e.g., $S_P \geq 20$) can still sustain for hours.

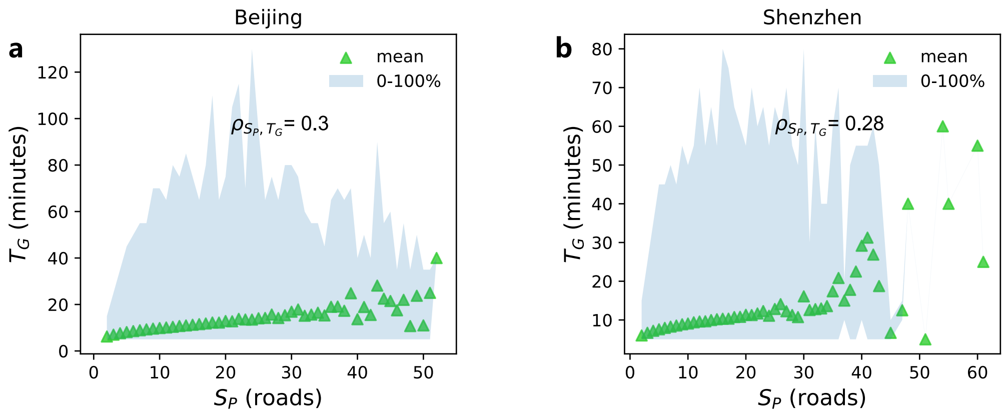

Supplementary Fig. 30. Relationship of growth duration T_G versus maximal size S_P on 18 workdays in Oct., 2015, in (a) Beijing and (b) Shenzhen. The bottlenecks included here have the maximal size $S_P \geq 2$. For Beijing, the *x*-axis displays the main part below 52. The growth duration T_G increases on average with S_P with large fluctuations. The Pearson correlation between S_P and T_G is 0.3 for Beijing and 0.28 for Shenzhen.

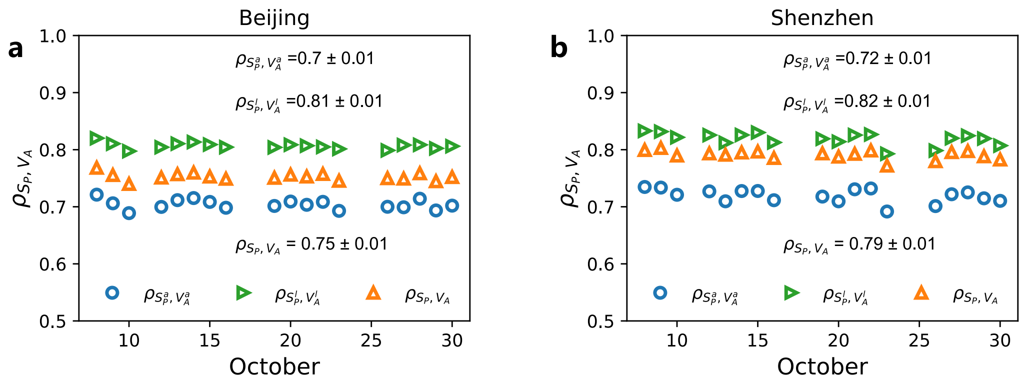

Supplementary Fig. 31. Correlations between the spatial maximal size and the growth speed obtained by three definitions of congestion size, for 18 workdays in October, 2015, in (a) Beijing and (b) Shenzhen. Based on the absorbed size $S_a(t)$, we computed the average growth speed V_A^a as the ratio between the maximal absorbed size S_P^a and the growth duration T_G^a, where S_P^a is the number of the absorbed congested roads when the size $S_a(t)$ reaches maximum. Based on the congestion length $S_l(t)$, we also calculate the average growth speed V_A^l as the ratio between the maximal length S_P^l and the growth duration T_G^l, where S_p^l is the length of the congestion component when the length $S_l(t)$ (kilometers) reaches maximum. Here, V_A^l means the average increased length (kilometers) of congested road segments per 5 minutes. The correlation $\rho_{S_P^a,V_A^a}$ is the Pearson correlation between the absorbed size S_P^a and its average growth speed V_A^a. The correlation $\rho_{S_P^l,V_A^l}$ is the Pearson correlation between the congestion length S_P^l and its average growth speed V_A^l. The correlation ρ_{S_P,V_A} is the Pearson correlation between the maximal congestion size S_P and its average growth speed V_A. The Pearson correlations in three definitions of size support our finding that

maximal jam size is highly and positively correlated to their growth speed.

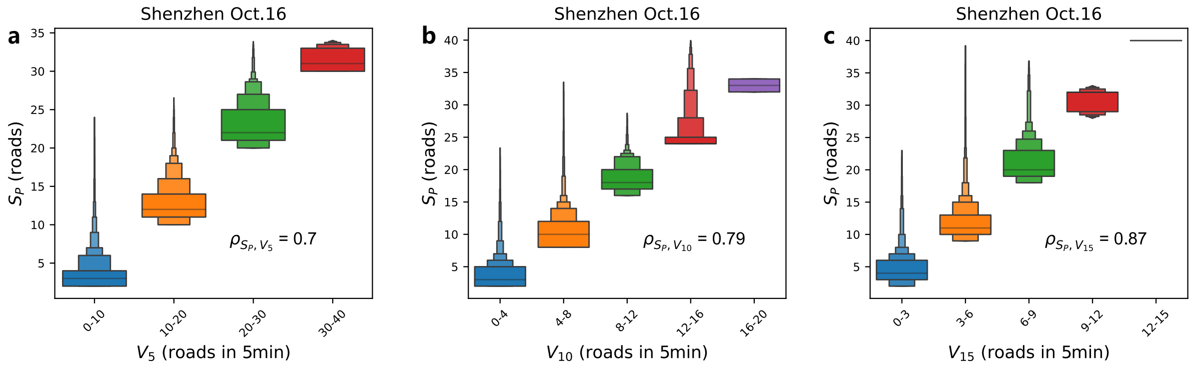

Supplementary Fig. 32. Box plots of maximal size S_P grouped by initial growth speed V_{T_I} obtained in different initial growth duration T_I, on October 16, 2015, in Shenzhen. The initial growth speed V_{T_I} is obtained in the initial growth duration of (**a**) 5 minutes, (**b**) 10 minutes and (**c**) 15 minutes. Here, V_{T_I} means the number of increased congested road segments in every 5 minutes during the early growth stage. The correlations between S_P and V_5, V_{10}, and V_{15} are respectively calculated for the congestion components with growth duration longer than or equal to 5 minutes, 10 minutes, and 15 minutes. The outliers are not displayed in the box plots. The figures indicate that the maximal size S_P of congestion components has a highly positive correlation with their growth speed even at a very early propagation stage.

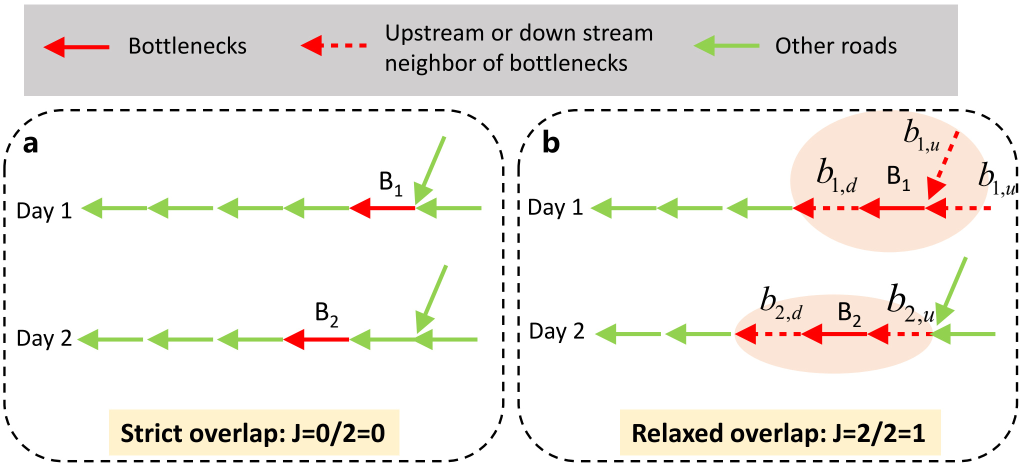

Supplementary Fig. 33. Demonstration of overlap calculations by the strict definition and the relaxed definition. a. By the strict definition, bottleneck B_1 on Day1 does not overlap with bottleneck B_2 on Day2, since the strict definition of overlap regards two bottlenecks being overlapped only if they emerge in the same road segment. **b**. By the relaxed definition, bottleneck B_1 on Day1 is regarded as overlapping with bottleneck B_2 on Day2, since the relaxed definition considers bottlenecks that emerged on 1-step nearby roads also overlapped. $b_{i,u}$ and $b_{i,d}$ are the 1-step upstream and downstream roads of the bottleneck B_i ($i = 1, 2$).

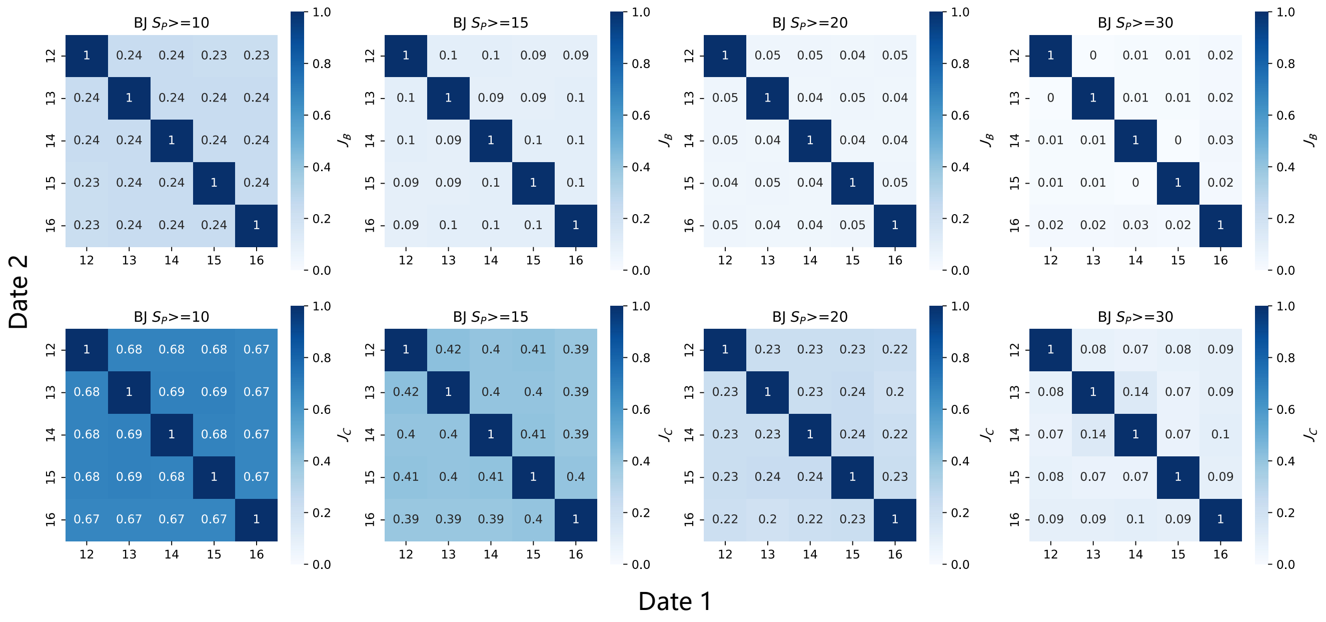

Supplementary Fig. 34. Jaccard index *J* for bottlenecks with maximal size S_P larger than a pre-defined threshold. The indices are calculated for every two days among five workdays, including October 12, 13, 14, 15, and 16, 2015, in Beijing. J_B is the Jaccard index for measuring the repetition of traffic bottlenecks between two workdays, and the four figures at the top are the J_B between every two days, respectively for bottlenecks with size S_P greater than or equal to 10, 15, 20 and 30. J_C is the Jaccard index for measuring the repetition of congestion components associated with the bottlenecks between two workdays, and the four figures at the bottom are the values of J_C respectively for bottlenecks with size S_P greater than or equal to 10, 15, 20 and 30. The smaller values of J_B than J_C indicate that the repetition of bottlenecks between different days is less than the repetition of their associated congestions. Notably, both the J_B and J_C are close to 0 if S_P is larger than 30.

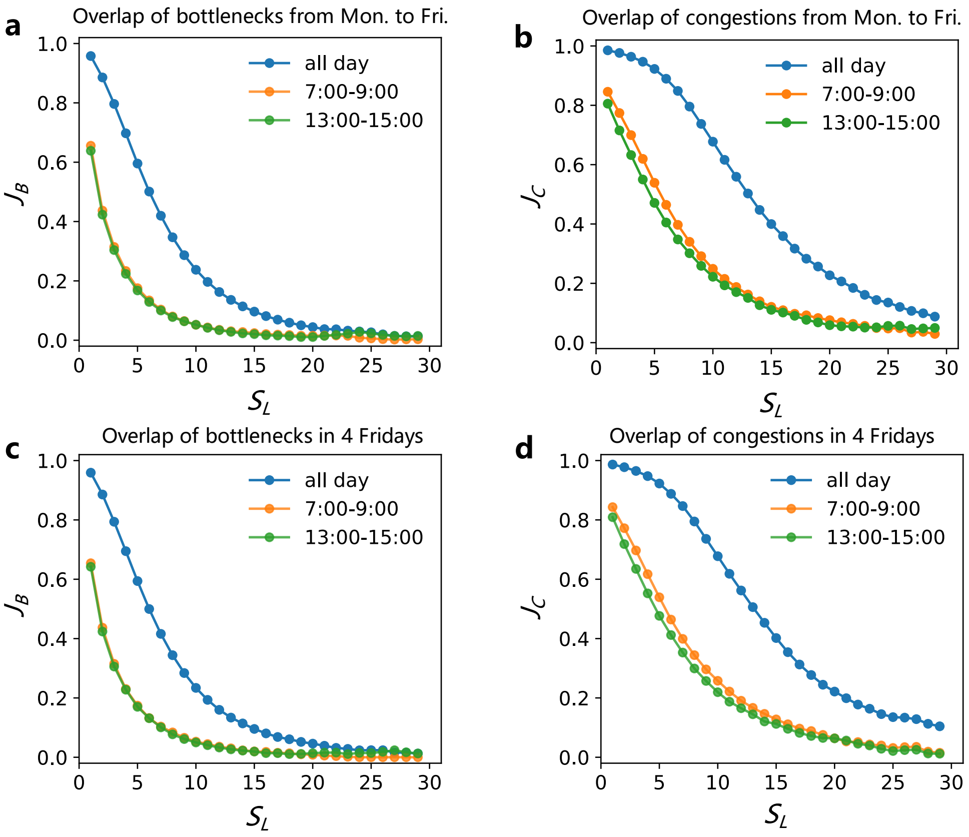

Supplementary Fig. 35. Jaccard indexes J_B for bottlenecks and J_C for their associated congestion components for bottlenecks under different threshold size S_L (i.e., bottlenecks with size $S_P \geqslant S_L$). J_B is the ratio between the size of overlapped bottlenecks and the size of the union of bottlenecks on two different days. J_C is the ratio between the size of the overlap of the associated congestions and the size of the union of the associated congestions on two different days. **a** and **b.** The Jaccard index (a) J_B and (b) J_C under different threshold size S_L for the bottlenecks in all day (0:00-24:00), rush hours (7:00 am-9:00 am) and non-rush hours (13:00 pm-15:00 pm), respectively. Each point is the mean value of the Jaccard index obtained between every two days among five workdays in a week, including October 12, 13, 14, 15, and 16, 2015, in Beijing. **c** and **d.** The Jaccard index (c) J_B and (d) J_C under different threshold size S_L for the bottlenecks in all day (0:00-24:00), rush hours (7:00 am-9:00 am) and non-rush hours (13:00 pm-15:00 pm), respectively. Each point is the mean value of the Jaccard index obtained between every two Fridays (Oct. 9, 16, 23, and 30, 2015) in 4 weeks, in Beijing. The curves show that repetition of both bottlenecks and their associated congestions decreases rapidly when S_L becomes larger, indicating that the major bottlenecks and their associated congested roads are rarely recurrent.

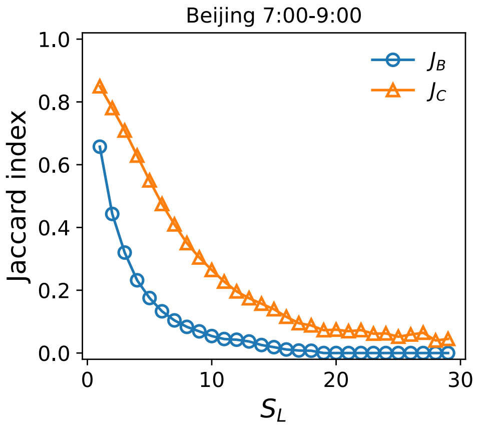

Supplementary Fig. 36. **Jaccard index** J_B **and Jaccard index** J_C **between two Fridays in different weeks, i.e., Oct. 9, and Oct. 16, 2015, in Beijing, for bottlenecks with different size threshold** S_L **(i.e.,** $S_P \geq S_L$**).** J_B is the ratio between the size of overlapped bottlenecks and the size of the union of bottlenecks in two different days. J_C is the ratio between the size of the overlap of the associated congestions and the size of the union of the associated congestions in two different days. The data is taken for rush hours 7:00 am -9:00 am.

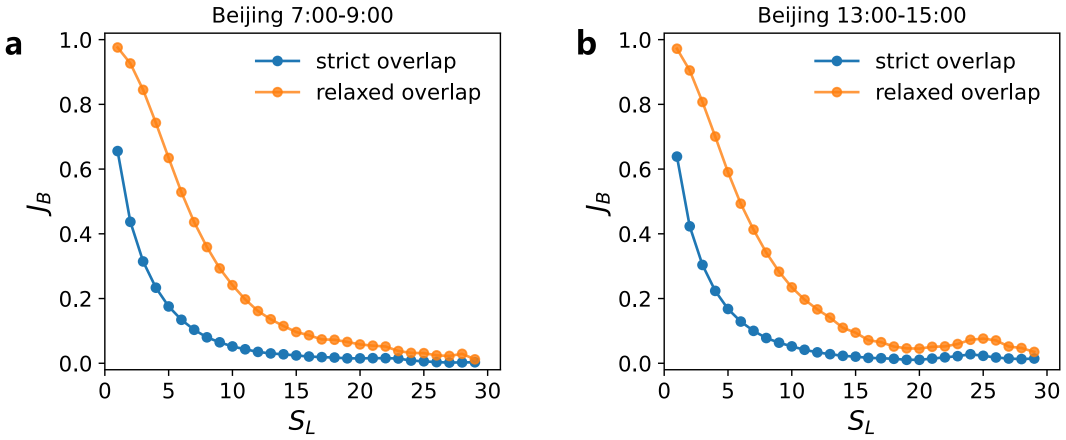

Supplementary Fig. 37. Jaccard indexes J_B **of the relaxed and strict overlaps for bottlenecks under different threshold size** S_L **(i.e., bottlenecks with size** $S_P \geqslant S_L$**) during (a) rush hours and (b) non-rush hours among different days.** We calculated the Jaccard indexes J_B for the bottlenecks between every two of 5 workdays (i.e., Oct. 12, 13, 14, 15 and 16, 2015, in Beijing) and average them.

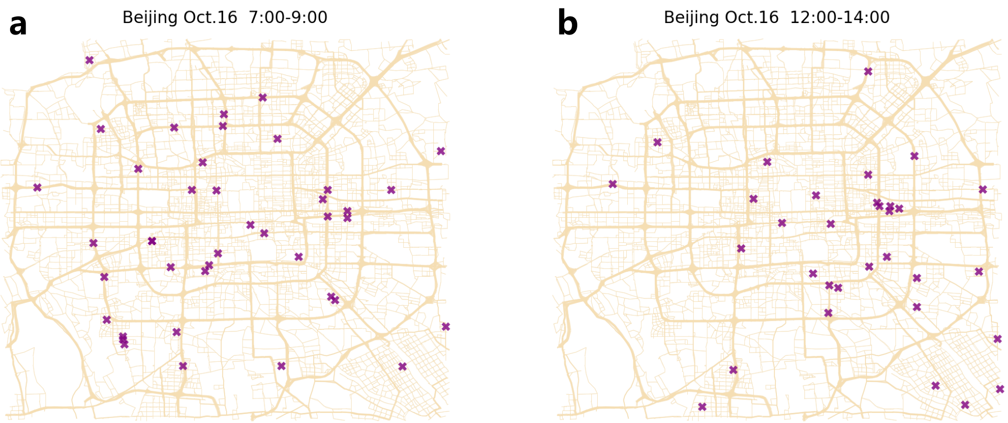

Supplementary Fig. 38. Examples of the false positive (purple stars) bottlenecks during (a) rush hours and (b) non-rush hours on Oct. 16, 2015, in Beijing. The false positive bottlenecks are the minor bottlenecks with S_P smaller than 20 roads yet wrongly predicted as major in the 15 minutes of their growth stage given the false positive rate being 5%. Here, the examples with $T_G \geq 15$ are displayed, and the probability P_i that a bottleneck is classified as major is based on the predictor V_{15}.

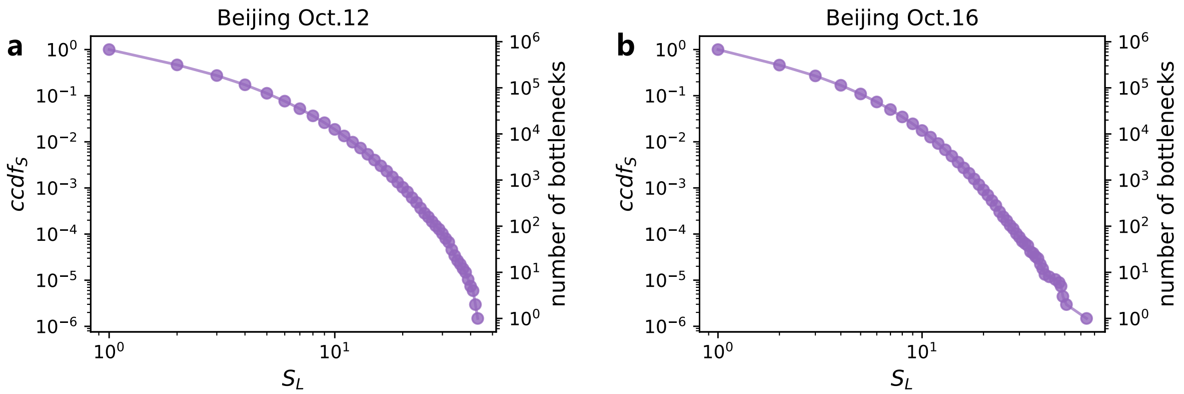

Supplementary Fig. 39. Complementary cumulative distribution function of size S_P on (a) Oct. 12, Monday and (b) Oct.16, Friday, 2015, in Beijing. The *x*-axis is the threshold size S_L. The left *y*-axis, i.e., the CCDF of the congestion size S_P, represents the probability of the bottleneck with the maximal size S_P larger than or equal to the threshold size S_L. The right *y*-axis is the number of the bottlenecks with maximal size S_P larger than or equal to the given threshold size S_L.

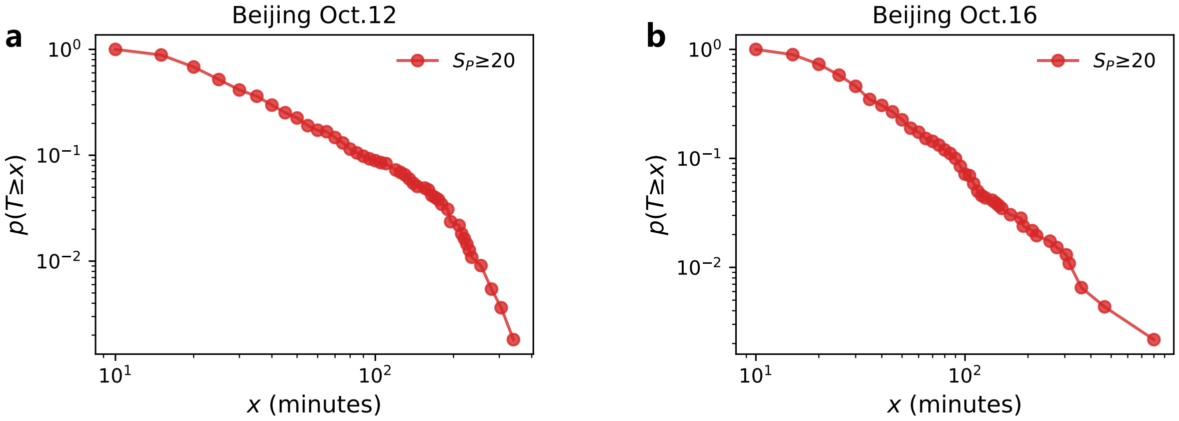

Supplementary Fig. 40. Complementary cumulative distribution function of the jam duration T for large congestion components with maximal size $S_P \geq 20$ roads on (a) Oct. 12, Monday and (b) Oct. 16, Friday, 2015, in Beijing. The y-axis, i.e., CCDF of T, is the probability that jam duration T longer than or equal to x.

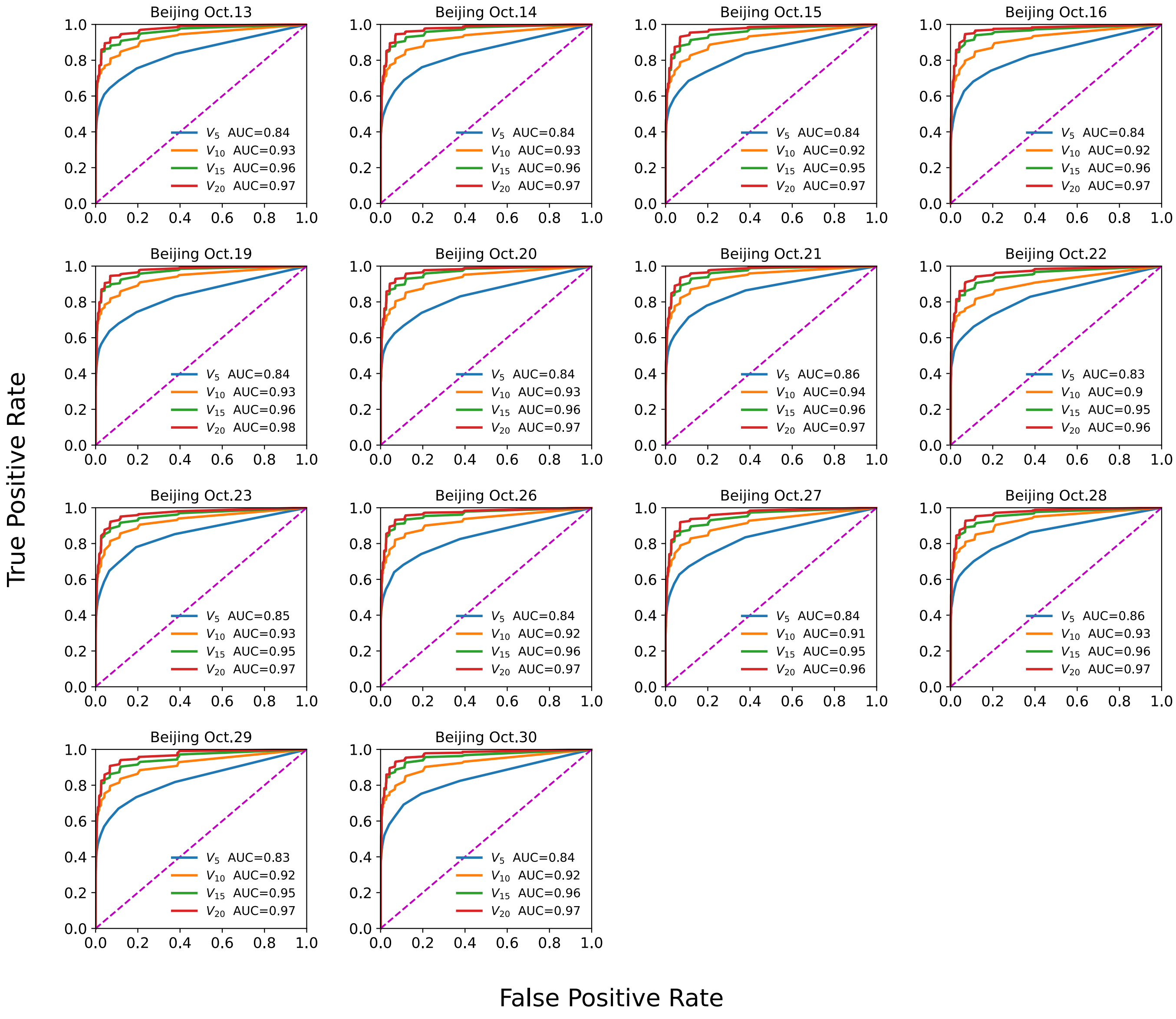

Supplementary Fig. 41. ROC curves of predicting whether a bottleneck could evolve into a major congestion component with maximal size greater than or equal to S_L=20, on 14 workdays in October 2015, Beijing. The results demonstrate the high consistency between the actual and the predicted major congestions based on prediction by their initial growth speed till the time T_I, since AUC values can increase to 0.95 if predicting the major bottlenecks at the first 15 minutes of their growth. The binary Probit model is trained based on the dynamics of the bottlenecks on Monday, October 12, 2015, Beijing.

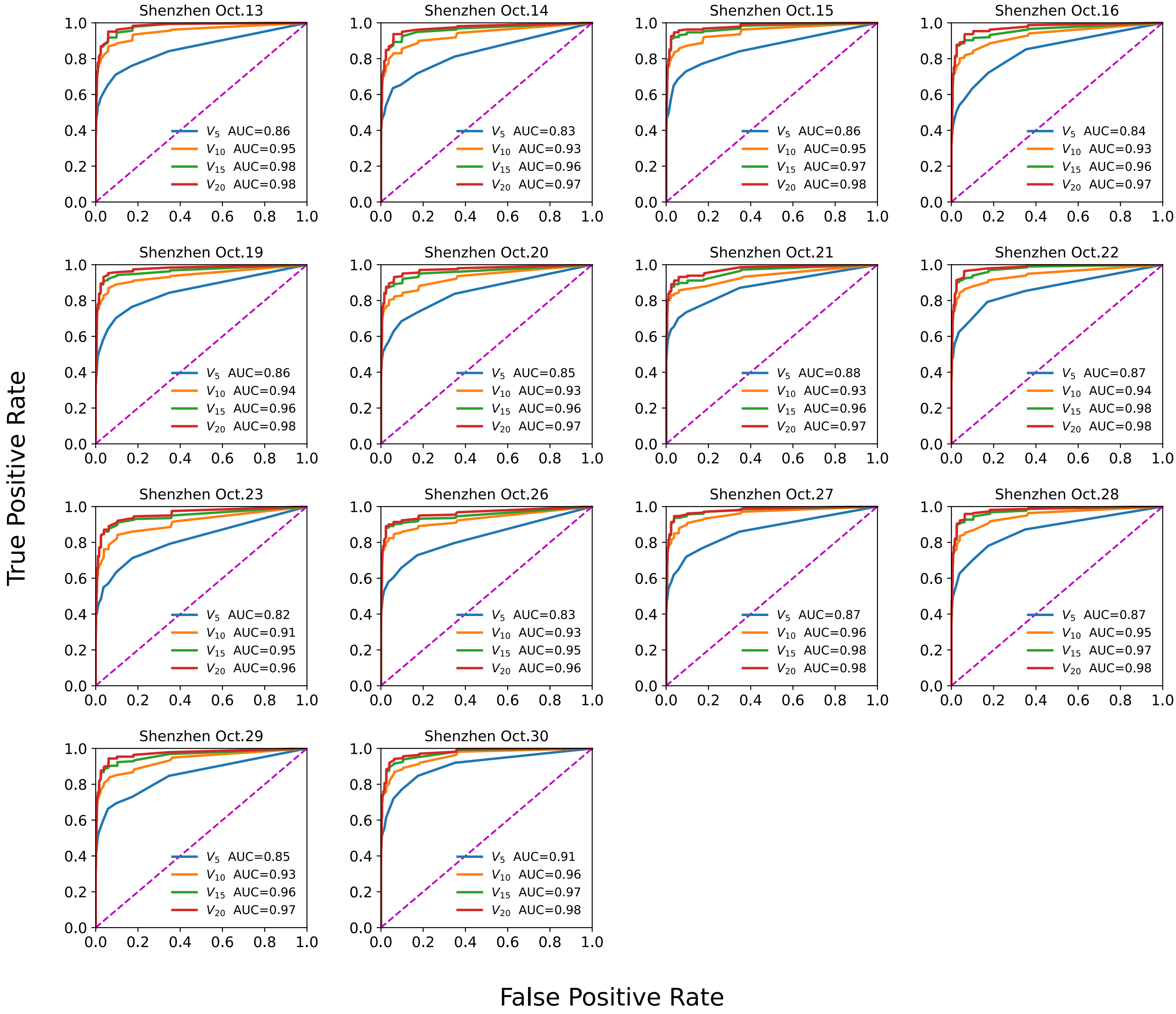

Supplementary Fig. 42. ROC curves of predicting whether a bottleneck could evolve into a major congestion component with maximal size greater than or equal to S_L=20, on 14 workdays in October 2015, Shenzhen. The results demonstrate the high consistency between the actual and the predicted major congestions based on prediction by their initial growth speed till the time T_I, since AUC values can increase to 0.95 if predicting the major bottlenecks at the first 15 minutes of their growth. The binary Probit model is trained based on the dynamics of the bottlenecks on Monday, October 12, 2015, Shenzhen.

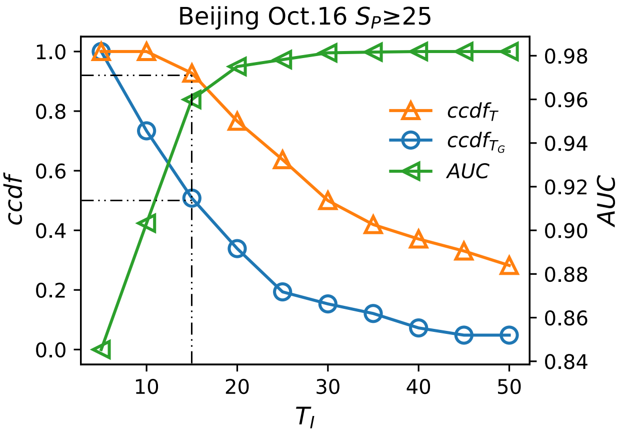

Supplementary Fig. 43. The CCDFs of growth duration T_G and total jam duration *T*, and AUC against the prediction time T_I for the major traffic congestions with size S_P larger than or equal to 25. The *x*-axis includes the prediction time of the initial 5 to 50 minutes of bottleneck growth. The left *y*-axis includes the CCDFs of growth duration T_G and total jam duration T. The right *y*-axis is the AUC of the prediction performance at the given prediction time T_I. The figures are plotted based on the bottleneck dynamics on Oct. 16, 2015, in Beijing.

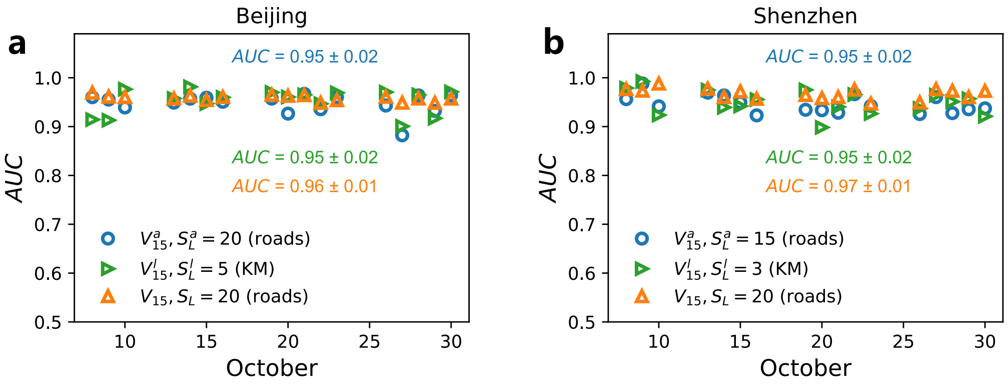

Supplementary Fig. 44. Prediction performances based on the initial growth speed obtained by three definitions of congestion size, on 17 workdays for Oct. 2015, in (a) Beijing and (b) Shenzhen. The prediction models are trained based on the dynamics of bottlenecks on Oct. 12, Monday, 2015. For the definition of absorbed size $S_a(t)$, we calculate the initial growth speed $V^a_{T_I}$ as the number of the absorbed congested roads at the initial growth duration T_I (e.g., 15 minutes) divided by duration T_I, and applied initial growth speed within the first 15 minutes to predict whether an absorbed congestion component would develop to be larger than or equal to the threshold size $S^a_L = 20$ (Beijing) or $S^a_L = 15$ (Shenzhen). For the definition of congestion length $S_l(t)$, we computed the initial growth speed $V^l_{T_I}$ of the congestions as the length of the congestions at the initial growth duration T_I divided by the duration T_I, and applied the initial growth speed within the first 15 minutes to predict whether the length of the congestions associated with a bottleneck would develop

to be longer than or equal to S_L^l=5 KM (Beijing) or S_L^l=3 KM (Shenzhen). For the definition of full congestion size $S(t)$, we use initial growth speed within the first 15 minutes to predict whether the congestions associated with a bottleneck would develop to be larger than or equal to $S_L = 20$. The AUC (the area under the ROC curve) values are around 0.95 for the predictions in three definitions of *S*, which supports the robustness of the prediction performances.

Supplementary Table 1. The *p*-values for the fitted exponential and power-law distributions of growth duration, on Oct. 16, 2015 in Beijing and Shenzhen. The *p*-value is calculated based on the fittings of T_G above 10 minutes. If p is large (commonly larger than 0.1)[15], the distance between the empirical data and the estimated curve can be considered as statistical fluctuations; if it is smaller than 0.1, the model may not be suitable to fit the empirical data. Note that the *p*-values for the exponential model of growth duration are 0.126 for Beijing and 0.191 for Shenzhen, supporting that the exponential model could well describe the main part of the distribution of T_G. In contrast, for the power law assumption, the *p*-values are much smaller than 0.1.

	Exponential	Power law
Beijing	**0.126**	0.003
Shenzhen	**0.191**	0.018

Supplementary References

1. Lighthill, M.J. & Whitham, G.B. On kinematic waves II. A theory of traffic flow on long crowded roads. *Proceedings of the Royal Society of London. Series A. Mathematical and Physical Sciences* **229**, 317-345 (1955).
2. Richards, P.I. Shock waves on the highway. *Operations Research* **4**, 42-51 (1956).
3. Biham, O., Middleton, A.A. & Levine, D. Self-organization and a dynamical transition in traffic-flow models. *Physical Review A* **46**, R6124 (1992).
4. Nagel, K. & Schreckenberg, M. A cellular automaton model for freeway traffic. *Journal de Physique I* **2**, 2221-2229 (1992).
5. Kerner, B.S. Experimental features of self-organization in traffic flow. *Physical Review Letters* **81**, 3797 (1998).
6. Kerner, B.S. & Rehborn, H. Experimental properties of phase transitions in traffic flow. *Physical Review Letters* **79**, 4030 (1997).
7. Kerner, B.S., Klenov, S.L. & Wolf, D.E. Cellular automata approach to three-phase traffic theory. *Journal of Physics A: Mathematical and General* **35**, 9971 (2002).
8. Arnott, R. & Small, K. The economics of traffic congestion. *American Scientist* **82**, 446-455 (1994).
9. Nagel, K., Wolf, D.E., Wagner, P. & Simon, P. Two-lane traffic rules for cellular automata: A systematic approach. *Physical Review E* **58**, 1425 (1998).
10. Hidas, P. Modelling lane changing and merging in microscopic traffic simulation. *Transportation Research Part C: Emerging Technologies* **10**, 351-371 (2002).
11. Daganzo, C.F. A behavioral theory of multi-lane traffic flow. Part II: Merges and the onset of congestion. *Transportation Research Part B: Methodological* **36**, 159-169 (2002).
12. Newell, G.F. A simplified theory of kinematic waves in highway traffic, part I: General theory. *Transportation Research Part B: Methodological* **27**, 281-287 (1993).

13. Daganzo, C.F. The cell transmission model: A dynamic representation of highway traffic consistent with the hydrodynamic theory. *Transportation Research Part B: Methodological* **28**, 269-287 (1994).

14. Daganzo, C.F. The cell transmission model, part II: network traffic. *Transportation Research Part B: Methodological* **29**, 79-93 (1995).

15. Clauset, A., Shalizi, C.R. & Newman, M.E. Power-law distributions in empirical data. *SIAM Review* **51**, 661-703 (2009).

16. Vickrey, W.S. Congestion theory and transport investment. *The American Economic Review* **59**, 251-260 (1969).

17. Arnott, R., De Palma, A. & Lindsey, R. Economics of a bottleneck. *Journal of Urban Economics* **27**, 111-130 (1990).

18. Ben-Akiva, M., De Palma, A. & Kanaroglou, P. Dynamic model of peak period traffic congestion with elastic arrival rates. *Transportation Science* **20**, 164-181 (1986).

19. Saberi, M., Hamedmoghadam, H., Ashfaq, M., Hosseini, S.A., Gu, Z., Shafiei, S., Nair, D.J., Dixit, V., Gardner, L. & Waller, S.T. A simple contagion process describes spreading of traffic jams in urban networks. *Nature Communications* **11**, 1616 (2020).

20. Zhang, L., Zeng, G., Li, D., Huang, H.-J., Stanley, H.E. & Havlin, S. Scale-free resilience of real traffic jams. *Proceedings of the National Academy of Sciences of the United States of America* **116**, 8673-8678 (2019).

21. Li, D., Fu, B., Wang, Y., Lu, G., Berezin, Y., Stanley, H.E. & Havlin, S. Percolation transition in dynamical traffic network with evolving critical bottlenecks. *Proceedings of the National Academy of Sciences* **112**, 669-672 (2015).

22. Brockmann, D. & Helbing, D. The hidden geometry of complex, network-driven contagion phenomena. *Science* **342**, 1337-1342 (2013).

23. Dakos, V., Scheffer, M., Van Nes, E.H., Brovkin, V., Petoukhov, V. & Held, H. Slowing down as an early warning signal for abrupt climate change. *Proceedings of the National Academy of Sciences* **105**, 14308-14312 (2008).

24. Akbarzadeh, M. & Estrada, E. Communicability geometry captures traffic flows in cities. *Nature Human Behaviour* **2**, 645-652 (2018).

25. Lee, M., Barbosa, H., Youn, H., Holme, P. & Ghoshal, G. Morphology of travel routes and the organization of cities. *Nature Communications* **8**, 2229 (2017).

26. Zhao, J., Li, D., Sanhedrai, H., Cohen, R. & Havlin, S. Spatio-temporal propagation of cascading overload failures in spatially embedded networks. *Nature Communications* **7**, 10094 (2016).

27. Çolak, S., Lima, A. & González, M.C. Understanding congested travel in urban areas. *Nature Communications* **7**, 10793 (2016).

28. Roughgarden, T. *Selfish routing and the price of anarchy*. (MIT Press, London, 2005).

29. Schröder, M., Storch, D.-M., Marszal, P. & Timme, M. Anomalous supply shortages from dynamic pricing in on-demand mobility. *Nature Communications* **11**, 1-8 (2020).

30. Dussutour, A., Fourcassié, V., Helbing, D. & Deneubourg, J.-L. Optimal traffic organization in ants under crowded conditions. *Nature* **428**, 70-73 (2004).

31. Yeung, C.H., Saad, D. & Wong, K.M. From the physics of interacting polymers to optimizing routes on the London Underground. *Proceedings of the National Academy of Sciences* **110**, 13717-13722 (2013).

32. Cantarella, G.E. & Cascetta, E. Dynamic processes and equilibrium in transportation

networks: towards a unifying theory. *Transportation Science* **29**, 305-329 (1995).

33. Vazifeh, M.M., Santi, P., Resta, G., Strogatz, S.H. & Ratti, C. Addressing the minimum fleet problem in on-demand urban mobility. *Nature* **557**, 534-538 (2018).

34. He, Q., Head, K.L. & Ding, J. Multi-modal traffic signal control with priority, signal actuation and coordination. *Transportation Research Part C: Emerging Technologies* **46**, 65-82 (2014).

35. Al-Madani, H.M. Dynamic vehicular delay comparison between a police-controlled roundabout and a traffic signal. *Transportation Research Part A: Policy and Practice* **37**, 681-688 (2003).

36. Le, T., Kovács, P., Walton, N., Vu, H.L., Andrew, L.L. & Hoogendoorn, S.S. Decentralized signal control for urban road networks. *Transportation Research Part C: Emerging Technologies* **58**, 431-450 (2015).

37. Hamedmoghadam, H., Jalili, M., Vu, H.L. & Stone, L. Percolation of heterogeneous flows uncovers the bottlenecks of infrastructure networks. *Nature Communications* **12**, 1254 (2021).

38. Zeng, G., Gao, J., Shekhtman, L., Guo, S., Lv, W., Wu, J., Liu, H., Levy, O., Li, D. & Gao, Z. Multiple metastable network states in urban traffic. *Proceedings of the National Academy of Sciences of the United States of America* **117**, 17528-17534 (2020).

39. Zeng, G., Li, D., Guo, S., Gao, L., Gao, Z., Stanley, H.E. & Havlin, S. Switch between critical percolation modes in city traffic dynamics. *Proceedings of the National Academy of Sciences of the United States of America* **116**, 23-28 (2019).
