# Supplementary material for: Spatiotemporal dynamics of traffic bottlenecks yields an early signal of heavy congestions
Source: Nat Commun. 2023 Dec 4;14:8002. doi: 10.1038/s41467-023-43591-7 (PMC10695996; doi:10.1038/s41467-023-43591-7)
Supplement: Supplementary file 2 — Peer Review File [file 41467_2023_43591_MOESM2_ESM.pdf]

nature portfolio

Peer Review File

Spatiotemporal dynamics of traffic bottlenecks: an early signal of heavy congestions

Open Access This file is licensed under a Creative Commons Attribution 4.0 International License, which permits use, sharing, adaptation, distribution and reproduction in any medium or format, as long as you give appropriate credit to the original author(s) and the source, provide a link to the Creative Commons license, and indicate if changes were made. In the cases where the authors are anonymous, such as is the case for the reports of anonymous peer reviewers, author attribution should be to 'Anonymous Referee' followed by a clear attribution to the source work. The images or other third party material in this file are included in the article's Creative Commons license, unless indicated otherwise in a credit line to the material. If material is not included in the article's Creative Commons license and your intended use is not permitted by statutory regulation or exceeds the permitted use, you will need to obtain permission directly from the copyright holder. To view a copy of this license, visit http://creativecommons.org/licenses/by/4.0/.

REVIEWER COMMENTS

Reviewer #1 (Remarks to the Author):

This review follows the Universal Principle Review approach. Lower numerical scores indicate higher quality just as in NIH scoring.

My scores are normalized by my expectations for publications in Nature Communications.

Quality of research: Score 2 (Range 1-3)

Duan et al. study the time dynamics of traffic bottlenecks with the goal of enhancing predictability of magnitude (extent and duration) of congestion events. They analyze traffic velocity data for two large cities in China for a period of 30 days in October 2015 at a resolution of 1 minute.

They report several findings. First, that the distribution of durations of growth and dissipation of bottlenecks decay as power laws. This is important because it means that even very long (much longer than the mean) congestions can occur in the system. Second, that the maximal number of congested road segments is positively correlated with the growth rate of the congestion event during its first 15 minutes. Third, they use this correlation to develop a predictive model for the prediction of whether the congestion will be a heavy one.

The topic of the study is very interesting and of practical impact to the many individuals that have to commute to work and back home. The ability to predict the final extent of a congestion event could be potentially very helpful to commuters and planners.

I feel, however, that this last opportunity is not really explored in the study. What I mean is that, if we can predict that a congestion event is going to be heavy, but if commuters have no alternative routes, then nothing with practical significance would be accomplished.

Additionally, the analysis does not make references to other issues that can contribute to the congestion and that could be equally or more predictive. Traffic studies from 3 decades ago suggested that congestion can arise spontaneously (someone thinks they see something on the road and breaks suddenly creating a cascade) when traffic density (demand) is high enough. But congestion can also arise

from well known causes: a highway exit/entry, a collision between vehicles, roadway construction, closing of lanes, etc. The reader does not know whether information on those sorts of events was available to the authors or not and whether they could have led to a different model for the size of the congestion event.

CONCERNS:

* The authors provide very few details on their data in the main text (line 89) and hardly more in the Methods section. This makes the evaluation of the research results difficult, if not impossible, to accomplish.

Specificaly, while some basic statistics are provided, much that is important is left unspecified. For example, how is the data gathered? road sensors or cell phone signals? What is the typical time coverage for each road segment? What about outliers? are the distributions of speeds over a 5-minute interval Gaussian? Was this tested?

* Representation of congestion events (Figs. 1 and S3). The use of arrows in not good since it blocks view of the roadways in the maps. Moreover, the choice of colors and their shades is not appropriate. The red-green color scheme is useless to color blind people.

I recommend using symbols of different sizes or different symbols to represent each of the three time periods highlighted and to place said symbols somewhere within the road segment. If using same symbols with different sizes or colors, please choose colors in sequentially logical manner instead of the current down and then up saturation).

* When defining S, the authors do not specify if there is a minimum length for the definition of a segment. The road network figures show some segments are too small to allow for a full arrow. Would the results change is some small segments were absorbed into the nodes?

Also, would other definitions of S that take into consideration not the number but the sum of the lengths of the road segments congested lead to any difference on the conclusions of the study?

* I do not understand the results of Figs. 3 and 4. Equation (7) states that V_A is proportional to S_p. So, of course the two variables would be correlated!

The only thing that could alter that correlation is if T_G is also a function of S_p, in which case studying the relationship between S_p and T_G would be the thing to do...

Also, I really do not understand what is the point of showing box plots. I suggest that the authors plot density surfaces instead since they provide more information.

* Captions of Figures in SI do not provide full information to interpret them.

* Data in Fig. S4c suggests that some cases of multiple DTBs could be due to data issues... This would affect the distributions of durations of growth and dissipation.

MAJOR CONCERNS:

* I am concerned that neither the data nor the code used in this study are being posted on public sites. Research has shown that statements about 'sharing data or code upon request' are rarely followed through. Code should be posted to a public GitHub repository and data should be posted to some public repository that assigns a DOI.

* The results of Figs. 2 and S5-S8 are quite unconvincing. I am surprised by a couple of things, first, that the authors do no plot the survival function instead of the probability density. Second, that they do not appear to have performed any model selection analysis (Clauset, Shalizi and Newman have actually published a Python package that makes such an analysis quite easy to implement).

Third, the power law fit to T_G is utterly unconvincing. The data is clearly convex and the exponent estimate is very larger, suggesting that an exponential functional form is likely a much better description of the data.

* I find that the authors do not provide compelling evidence for the lack of recurrence of the heavy congestion events. One would not expect large recurrence when comparing rush versus non-rush hours, but would expect large recurrence when comparing rush hour in different workdays. Moreover, the low Jaccard index for bottlenecks associated with large congestions is not surprising, since one could expect the actual starting road segment to possibly vary within some close set of road segments.

I think it would be important to consider other definitions of overlap between congestion events so that one can be convinced of the robustness of the conclusions. I also thing that the manuscript could benefit from some diagrams in the SI showing how overlap is calculated.

Reproducibility of research: Score 3 (Range 1-3)

I found the supplementary information to contain too little information about how data was gathered and there is no intent to publicly share the data or the code/software used to analyze the data.

I find this unacceptable!

Completeness of research: Score 2 (Range 1-3)

I think the authors systematically choose a single way to calculate some quantity when other choices could potentially have altered their conclusions. I believe that some additional degree of sensitivity analysis of the results would help strengthen the study.

Scholarship: Score 1 (Range 1-3)

I find the the review of prior literature thorough and compelling.

Impact of research: Score 2 (Range 1-4)

I believe that the authors did a great job of motivating their study and I am fully persuaded by the importance of the topic. I am, however, not fully convinced by the significance of their findings and believe that some of the analyses need to be strengthened in order to increase the impact of the study.

Reviewer #2 (Remarks to the Author):

Overall, the research is interesting, novel and a contribution to the body of scientific body of knowledge on traffic science. The developed approach examines traffic congestion on a highly aggregate scale but in quite creative ways. While the work is of value to traffic science, the motivation related to signalization could benefit from a bit further coverage given the significant range of research which has been done related to real-time monitoring for traffic control.

Specific Comments:

1. As per your definition S(t) is the number of congested links – but, by integrating the difference in the number of congested and dissipated links over time would this not give you the duration of overall congestion and not the number of congested links? (Eq -3)

a. Same with eq-4 in the supplementary text.

2. It seems that generalizing the statement that dissipation takes twice more than the build-up could be further discussed. Since the article is studying the network level, not at a corridor. if the flow in one corridor is high for a prolonged period, it causes the dissipation to last longer, and it need not be because of the initial congestion bottleneck but can also be because of traffic control devices?

3. Any reason why the beta‘s on the weekend is similar to a weekday and not a holiday or some other value?

4. Line 304- In traffic studies, it tends to be more common to check how the patterns are based on the day of the week not across days within a week.

5. Line 105 – Should it be “emergence”, not “emergency”?

6. Some sentences might benefit from a bit further refinement – e.g., …we follow the lifecycle of each dynamics of traffic bottlenecks (DTB) in the entire road networks…

7. In supplementary figures 12 and 13, the trend is counter-intuitive, because larger congestions should take more time to dissipate than smaller congestion however all plots seem to show opposite trend. Could this be explained?

8. The predicator analysis could benefit from a bit further attention:

-To better understand the efficacy of the predictor, it is important to mention the overall congestion stats, i.e., a histogram of congestion size Sp to indicate the share of SL > 20 that are actually being predicted. Then, an appropriate mention of the actual time duration distributions of all such larger congestions.

-Supp. fig 12 and 13 seem to show that larger congestions do not have very large time durations. Then what is the efficacy left of using a V15 predictor that detects congestion after 15 mins at which time most of the detected large congestions are close to finishing the growth stage. The analysis could include the actual growth trends against prediction times.

9. Why are J_c values higher than J_b, are they calculated for all congestions or for those where the J_b is high? Can the authors clarify the fact that even when bottleneck or J_b is less similar still the associated congestions show more similarity?

10. Using the term 'free-flow traffic state' for non-congested links is not generally common usage in traffic science. Since the congestion weight < 0.5 (eq. 9) won't represent free flow conditions. I suggest using 'uncongested-state' vs 'congested-state'. An uncongested state might still have travel times somewhat higher than free flow.

11. Fig. 5, it would also be interesting to show false positives or the false alarms that the predictor generates.

Reviewer #3 (Remarks to the Author):

The manuscript is built upon a method of identifying traffic bottleneck links and observing the propagation and dissipation of traffic jams corresponding to these bottlenecks. The results show that recovery and growth patterns of traffic jams are distinctively different, using the evidence from real traffic data collected from two city networks. The main finding is that the growth speed of congestion

measured in 5-15 mins of the emergence is significantly correlated with the maximal size of the growing congestion pocket, and thus can be used to make accurate predictions of major traffic jams. The idea of the paper is original and the results are immensely useful and both missing in the existing literature, and therefore, I recommend publication of the manuscript after necessary revisions rendered by the comments below.

1. A general comment on the main text is that in many parts it deviates from the main message. As an example, the material written around equations 2 and 3 did not belong to the main text in my opinion. The manuscript can become much sharper and better deliver its main finding by highlighting the main theme, which is as suggested by the title, is the existence of early signal of heavy congestion in real traffic network data.

2. I found the explanations supporting the observed difference between growth and recovery speeds troubling. The scaling laws derived from the data seem valid and very useful, however, the quite trivial explanation should be the fact that vehicular flows arrive at the congested component with a much higher speed than they leave the jam. The analysis given in lines 184-196 lacks rigor. Basically, the mathematics presented, only suggest that the slowest congestion can grow is at the rate of 0 but there is no upper bound. I strongly suggest that authors remove that paragraph and explain the phenomenon through domain knowledge of traffic network flows.

3. In Fig. 2 for both cities <r> is inversely changing relative to \beta_R, and perhaps surprisingly, the relationship is not as clear with \beta_G. Is the average recovery to growth rate is heavily influenced by the heavy tail in recovery duration distribution? It would be interesting to investigate this, and possibly find an explanation for the phenomenon.

In the following I present a separate list of comments which I consider to be of minor importance compared to the above.

4. In two places, it is suggested that the growth/recovery discrepancy in traffic resembles natural phenomena such as spread of a virus. Unfortunately, this is due to very different but quite apparent reasons. As suggested in another comment, the high speed of vehicles when arriving to the jam and their departure starting at very low speed, explains this phenomenon in traffic networks. I feel the statements can be misleading to some portions of the audience for this paper and recommend that it would be removed or majorly revised.

5. DTB is introduced as the acronym for “dynamics of traffic bottlenecks”, but then used all over the text, seemingly, to refer to “bottlenecks”. After authors introduce the method used to find the trunks, simply using “bottleneck (link)” is superior to the use of “DTB” to refer to those links.

6. When observing the recovery of a congested component, does the algorithm: i) count the (decreasing number of) connected road segments that remain congested over time? Or ii) follows the set of congested roads recorded at the peak of the component size? Also, the trunk may recover sooner than the whole congested component, and after its disappearance other neighboring roads may become

congested. Will they be considered as branches of the same tree even though they appeared after the bottleneck's recovery? Please clarify this in the Methods section or the main text.

7. In Fig. 2 for each congested component, propagation and dissipation durations are plotted in a & b panels. Why are there so many more data points associated with T_R, although they should be equal. Is there a missing explanation or plotting error there?

8. The Jaccard index of 0.05 indicates that 5% of all bottlenecks appearing in two days are repeated in both days. This has a subtle difference to what is stated in the manuscript.

9. A concise explanation of the bottleneck identification method (presented in earlier works) in the main text can aid the reader's understanding and will improve the manuscript.

Responds to the reviewers' comments of

"Spatiotemporal dynamics of traffic bottlenecks: an early signal of heavy congestions"

by Jinxiao Duan, Guanwen Zeng, Nimrod Serok, Daqing Li, Efrat Blumenfeld Lieberthal*,
Hai-Jun Huang* and Shlomo Havlin*

(Manuscript No.: NCOMMS-23-10587)

We would like to first thank the anonymous reviewers for their valuable comments and suggestions. Listed below is a summary of the main changes, followed by an itemized response to all review comments.

Reviewer #1 (Remarks to the Author):

Duan et al. study the time dynamics of traffic bottlenecks with the goal of enhancing predictability of magnitude (extent and duration) of congestion events. They analyze traffic velocity data for two large cities in China for a period of 30 days in October 2015 at a resolution of 1 minute.

They report several findings. First, that the distribution of durations of growth and dissipation of bottlenecks decay as power laws. This is important because it means that even very long (much longer than the mean) congestions can occur in the system. Second, that the maximal number of congested road segments is positively correlated with the growth rate of the congestion event during its first 15 minutes. Third, they use this correlation to develop a predictive model for the prediction of whether the congestion will be a heavy one.

The topic of the study is very interesting and of practical impact to the many individuals that have to commute to work and back home. The ability to predict the final extent of a congestion event could be potentially very helpful to commuters and planners.

Response: We thank the reviewer very much for the insightful comments, and for considering our paper "very interesting and of practical impact" and "potentially very helpful to commuters and planners". We have carefully considered all the comments, and thoroughly addressed all the concerns of the reviewer. Below we provide our point-by-point responses.

I feel, however, that this last opportunity is not really explored in the study. What I mean is that, if we can predict that a congestion event is going to be heavy, but if commuters have no alternative routes, then nothing with practical significance would be accomplished.

Response: We thank the reviewer very much for this valuable comment. Our prediction results may help commuters in the following way. On one hand, warning the travelers with alternative routes to be far away from the predicted heavy congestion regions (Yeung et al., 2013; Vazifeh et al., 2018) could alleviate the traffic flows in predicted areas. On the other hand, rescheduling departure time of the potential travelers with no alternative routes by road pricing strategies (Schröder et al., 2020) could reduce traffic demand in the predicted area.

These methods of demand management have also been found effective in alleviating congestion in earlier studies (Alonso-Mora et al., 2017; Lee et al., 2017; Akbarzadeh and Estrada, 2018). Moreover, coordinated management from supply side could also help to alleviate the traffic on the predicted heavy congestions (Çolak et al., 2016). We have now clarified this important issue in the Discussion of the revised Main Text.

References:

Akbarzadeh, M., Estrada, E. 2018. Communicability geometry captures traffic flows in cities. Nature Human Behaviour 2 (9), 645-652.

Alonso-Mora, J., Samaranayake, S., Wallar, A., Frazzoli, E., Rus, D. 2017. On-demand high-capacity ride-sharing via dynamic trip-vehicle assignment. Proceedings of the National Academy of Sciences 114 (3), 462-467.

Çolak, S., Lima, A., González, M.C. 2016. Understanding congested travel in urban areas. Nature Communications 7, 10793.

Lee, M., Barbosa, H., Youn, H., Holme, P., Ghoshal, G. 2017. Morphology of travel routes and the organization of cities. Nature Communications 8 (1) 2229.

Schröder, M., Storch, D.-M., Marszal, P., Timme, M. 2020. Anomalous supply shortages from dynamic pricing in on-demand mobility. Nature communications 11 (1), 4831.

Vazifeh, M.M., Santi, P., Resta, G., Strogatz, S.H., Ratti, C. 2018. Addressing the minimum fleet problem in on-demand urban mobility. Nature 557 (7706), 534-538.

Yeung, C.H., Saad, D., Wong, K.M. 2013. From the physics of interacting polymers to optimizing routes on the London Underground. Proceedings of the National Academy of Sciences 110 (34), 13717-13722.

Additionally, the analysis does not make references to other issues that can contribute to the congestion and that could be equally or more predictive. Traffic studies from 3 decades ago suggested that congestion can arise spontaneously (someone thinks they see something on the road and breaks suddenly creating a cascade) when traffic density (demand) is high enough. But congestion can also arise from well known causes: a highway exit/entry, a collision between vehicles, roadway construction, closing of lanes, etc. The reader does not know whether information on those sorts of events was available to the authors or not and whether they could have led to a different model for the size of the congestion event.

Response: We thank the reviewer very much for this helpful comment. We further improve our revised manuscript accordingly.

For congestion emerged spontaneously, attention is often paid to congestion phase transition (Helbing, 2001; Daganzo, 2007). For example, the kinematic wave theory (Lighthill and Whitham, 1955; Richards, 1956) explains the transition from free flow to congestion as the result of wave interactions. The cellular automaton (CA) models (Biham et al., 1992; Nagel and Schreckenberg, 1992) have shown traffic jams can spontaneously form when the density is high enough. The three-phase traffic theory (Kerner and Rehborn, 1997; Kerner, 1998; Kerner et al., 2002) has found that traffic phase transition can occur in free flow, synchronized flow, and congested phases. These phase transition phenomena are helpful in understanding congestion occurrences and in developing emergency response plans.

For congestion emerged from well known causes, much attention has been directed on jam formations. For example, Newell (1993) proposed a simplified theory of kinematic waves in analyzing congestion hysteresis phenomenon on a highway exit/entry. The cell transmission

model (CTM) (Daganzo, 1994, 1995) has considered vehicle conservation and described traffic jams as a series of cells in temporary obstructions, such as a collision between vehicles. The famous queue model (Arnott and Small, 1994) has described the congestion formation behavior nearby a bottleneck when its capacity is reduced or demand is increased, such as caused by roadway construction. The lane-changing model (Nagel et al., 1998; Daganzo, 2002; Hidas, 2002) has described the decision-making behavior when some lanes are blocked or closed. Such models are helpful in understanding the jam formation in the microscopic scale.

The above studies have developed excellent macroscopic or microscopic approaches in understanding congestion (Arnott and Small, 1994; Wright and Roberg, 1998). Meanwhile, in the network scale, it remains unclear how congestion emerges and propagates spatiotemporally, especially in megacities from real data. We have focused on this important yet missing point. Our method describes patterns of spatiotemporal congestion propagation and dissipation in real urban networks in megacities. In one way, our findings can provide data explanations for the well-established models. For example, our identified scaling patterns in *real urban networks* can well explain and expand the 1-dimensional scaling results modelled in existing studies (Nagatani, 1995; Petri et al., 2013). In another way, our findings may help to extend mesoscopic traffic models in the urban network.

We have revised the Introduction and Discussion to highlight the contributions of these important studies and discussed the innovations of our method in the revised Main Text.

References:

Arnott, R., Small, K. 1994. The economics of traffic congestion. American Scientist 82 (5), 446-455.

Biham, O., Middleton, A.A., Levine, D. 1992. Self-organization and a dynamical transition in traffic-flow models. Physical Review A 46 (10), R6124.

Daganzo, C.F. 1994. The cell transmission model: A dynamic representation of highway traffic consistent with the hydrodynamic theory. Transportation Research Part B: Methodological 28 (4), 269-287.

Daganzo, C.F. 1995. The cell transmission model, part II: network traffic. Transportation Research Part B: Methodological 29 (2), 79-93.

Daganzo, C.F. 2002. A behavioral theory of multi-lane traffic flow. Part II: Merges and the onset of congestion. Transportation Research Part B: Methodological 36 (2), 159-169.

Daganzo, C.F. 2007. Urban gridlock: Macroscopic modeling and mitigation approaches. Transportation Research Part B: Methodological 41 (1), 49-62.

Helbing, D. 2001. Traffic and related self-driven many-particle systems. Reviews of modern physics 73 (4), 1067.

Hidas, P. 2002. Modelling lane changing and merging in microscopic traffic simulation. Transportation Research Part C: Emerging Technologies 10 (5-6), 351-371.

Kerner, B.S. 1998. Experimental features of self-organization in traffic flow. Physical review letters 81 (17), 3797.

Kerner, B.S., Klenov, S.L., Wolf, D.E. 2002. Cellular automata approach to three-phase traffic theory. Journal of Physics A: Mathematical and General 35 (47), 9971.

Kerner, B.S., Rehborn, H. 1997. Experimental properties of phase transitions in traffic flow. Physical Review Letters 79 (20), 4030.

Lighthill, M.J., Whitham, G.B. 1955. On kinematic waves II. A theory of traffic flow on long crowded roads. Proceedings of the Royal Society of London. Series A. Mathematical and Physical Sciences 229 (1178), 317-345.

Nagatani, T. 1995. Self-organized criticality and scaling in lifetime of traffic jams. Journal of the Physical Society of Japan 64 (1), 31-34.

Nagel, K., Schreckenberg, M. 1992. A cellular automaton model for freeway traffic. Journal de physique I 2 (12), 2221-2229.
Nagel, K., Wolf, D.E., Wagner, P., Simon, P. 1998. Two-lane traffic rules for cellular automata: A systematic approach. Physical Review E 58 (2), 1425.
Newell, G.F. 1993. A simplified theory of kinematic waves in highway traffic, part I: General theory. Transportation Research Part B: Methodological 27 (4), 281-287.
Petri, G., Expert, P., Jensen, H.J., Polak, J.W. 2013. Entangled communities and spatial synchronization lead to criticality in urban traffic. Scientific reports 3 (1), 1-8.
Richards, P.I. 1956. Shock waves on the highway. Operations Research 4 (1), 42-51.
Wright, C., Roberg, P. 1998. The conceptual structure of traffic jams. Transport Policy 5 (1), 23-35.

CONCERNS:

Comment R1C1: The authors provide very few details on their data in the main text (line 89) and hardly more in the Methods section. This makes the evaluation of the research results difficult, if not impossible, to accomplish.

Specificaly, while some basic statistics are provided, much that is important is left unspecified. For example, how is the data gathered? road sensors or cell phone signals? What is the typical time coverage for each road segment? What about outliers? are the distributions of speeds over a 5-minute interval Gaussian? Was this tested?

Response: We thank the reviewer very much for this important comment. We have provided more details on the datasets as follows.

(1) How is the data gathered? Road sensors or cell phone signals? What is the typical time coverage for each road segment?

The traffic data are gathered from numerous trajectory records by the GPS devices in floating cars (e.g., taxies and private cars), and the velocity data are aggregated and matched to each road segment (link) by the data provider from the gathered traffic data. The time coverage of the data for each road segment is from 0:00 to 24:00 (with a resolution of 1 minute) for each day between Oct. 1 and Oct. 30, 2015. We have added these descriptions of data in Subsection 2.1 and Methods of the revised Main Text.

(2) What about outliers?

We have regarded the road velocity record as an outlier point if it is missing for a specific instant in our dataset, which could be due to few floating cars passing through the road at the specific time. The outliers of the velocity records in the main roads account for around 3-5% of their total velocity records at most time (6:00 am-24:00 pm and 0:00 am-2:00 am) and 10-20% at midnight (2:00 am-6:00 am).

When handling outliers, we regarded a road with outlier velocity as congestion state, if it connects its upstream and downstream roads that have become congested in the predefined chronological order. Specifically, for an outlier road, if its downstream road has been congested earlier than its upstream road, with the difference of their congestion time smaller than a predefined time-interval, i.e., 2θ (θ set as 10 minutes here) (Fig. **R1**), it is regarded as a congestion state. Otherwise, it is regarded as uncongested. In general, around 2% of the outliers have been assigned as congested states. We have clarified these analyses of the

outliers in Supplementary Note 1 and Supplementary Fig. 1 of the revised SI, and discussed them in Methods of the revised Main Text.

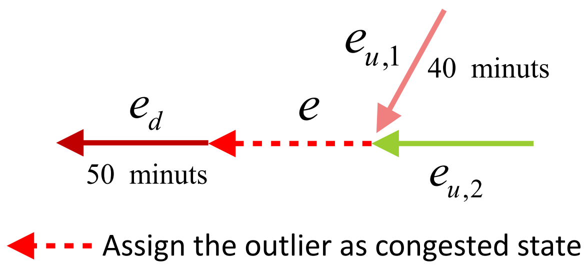

Fig. R1. Demonstration of assigning the traffic state for a specific road with an outlier. The arrows of the links are the directions of traffic flow. The road e is assigned as a congested state since its downstream road e_d has been congested 10 minutes earlier than its upstream road $e_{u,1}$. The solid links are the roads with normal velocity records, the dashed links are the roads with an outlier of velocity record. The red links are the congested roads (the darker red means the congestion has lasted for a longer time). The green links are the uncongested roads. The number below the link is the time duration that the congested road has been continuously congested up to now.

(3) Are the distributions of speeds over a 5-minute interval Gaussian? Was this tested?

We have tested here if distribution of the road velocity over 5-minute interval is close to Gaussian. To plot the distribution of velocity over a 5-minute interval for all the roads, we first rescale the velocity records in the given 5-minute interval of a road to be the relative deviation of the velocity from its mean. The rescaled velocity $U_e^S(i)$ of road segment e at minute i ($i = 1,2,3,4,5$) is

$$U_e^S(i) = \frac{U_e(i) - U_e^a}{U_e^a}, \qquad \text{(R1)}$$

where $U_e(i)$ is the actual velocity record of road e at minute i, and U_e^a is the mean value of the actual velocity records of road e in the given 5-minute interval. In Fig. **R2**, it is seen that the empirical distribution of U_e^S shows a somewhat fat-tail feature compared with the fitted Gaussian distributions during both typical periods. The p-values of the Kolmogorov-Smirnov KS statistic (Clauset et al., 2009) are $p < 0.0001$ for all the estimated distributions, indicating that the rescaled velocity deviates from the Gaussian distribution. The tails of the actual data are somewhat broader than the estimated Gaussian distributions, meaning that a small fraction of velocity records may have a large deviation from its mean value over 5-minute interval. However, the standard errors are relatively small, indicating that the road velocity records are generally stable over a 5-minute interval. We have added all these results in Supplementary Note 1 and Supplementary Fig. 2 of the revised SI, and discussed them in Methods of the revised Main Text.

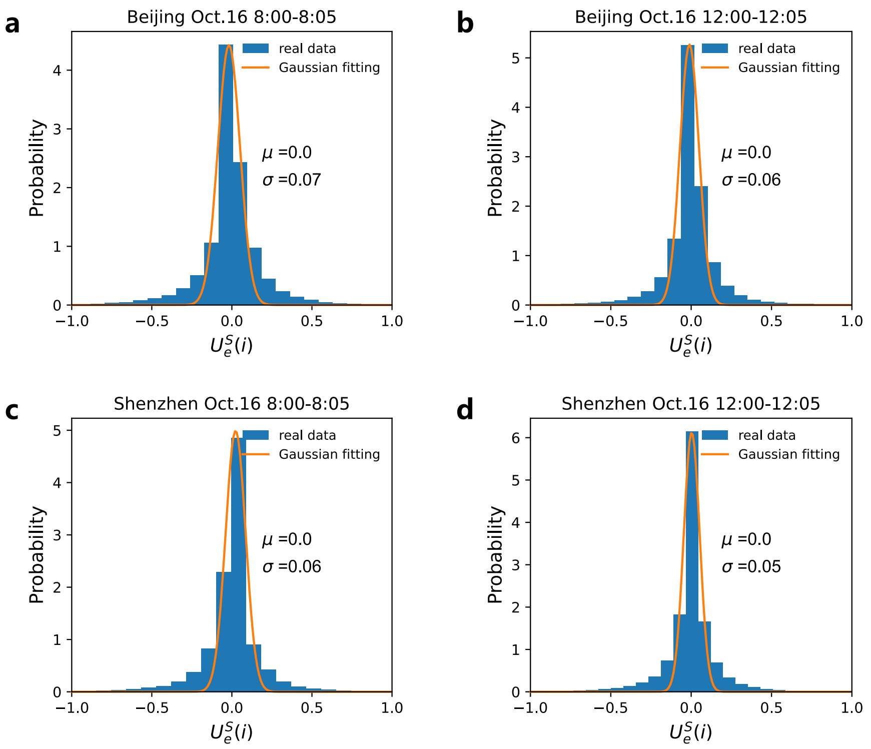

Fig. R2. Distributions of rescaled velocity U_e^S over a 5-minute interval in Beijing and Shenzhen. **a** and **b**. Distributions of the empirical rescaled velocity and the fitted curves of Gaussian model, in (**a**) rush hours (8:00-8:05) and (**b**) non-rush hours (12:00-12:05) on Oct. 16, 2015, in Beijing. **c** and **d**. Distributions of the empirical rescaled velocity and the fitted curves of Gaussian distributions, in (**c**) rush hours (8:00-8:05) and (**d**) non-rush hours (12:00-12:05) on Oct. 16, 2015, in Shenzhen. In the given 5-minute interval, the distribution includes velocity records in all the roads.

References:

Clauset, A., Shalizi, C.R., Newman, M.E. 2009. Power-law distributions in empirical data. SIAM review 51 (4), 661-703.

Comment R1C2: Representation of congestion events (Figs. 1 and S3). The use of arrows in not good since it blocks view of the roadways in the maps. Moreover, the choice of colors and their shades is not appropriate. The red-green color scheme is useless to color blind people.

I recommend using symbols of different sizes or different symbols to represent each of the three time periods highlighted and to place said symbols somewhere within the road segment. If using same symbols with different sizes or colors, please choose colors in sequentially logical manner instead of the current down and then up saturation).

Response: We thank the reviewer very much for this suggestion. We have replotted the corresponding figures to improve their readability, as shown in Figs. **R3** and **R4** below. In the revised version, we have designed lines of different styles, sizes, and colors to represent the congested road segments at different times. We have also modified the saturation of the colors to make it sequentially logical. Since the arrows represent the directions of traffic flow

and are important for bottleneck identifications, we adjusted the size and style of the arrows and the lines to block less view of the roadways in the maps. We have updated Figs. **R3** and **R4** being Fig. 1 of the revised Main Text, and Supplementary Fig. 3 of the revised SI.

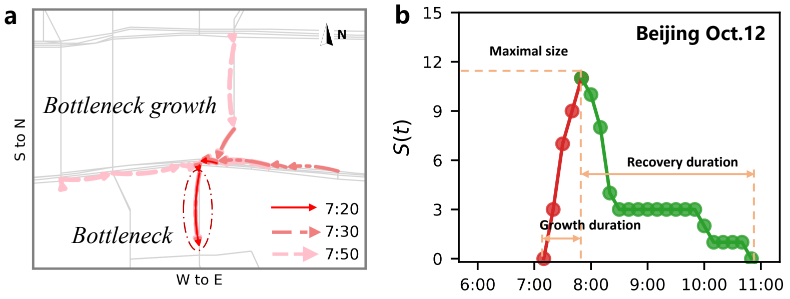

Fig. R3. Propagation and dissipation of a typical bottleneck in Beijing traffic network. a. Several snapshots of the congested road segments during growth stage at 7:20, 7:30, and 7:50 for a specific but typical bottleneck on Monday, October 12, 2015, in Beijing. The congestions originated from the bottleneck (circled red solid links), and gradually developed to more congestions (light red dashed links) in the upstream neighbouring streets. The arrows of the links are the directions of traffic flow. **b**. The size S changes over time. This congestion component lasted for more than 3 hours after its bottleneck emerged. Its maximal size S_P reached 11 road segments at 7:50, and then the size S began to decrease until it completely vanished at 10:50. **c**. Several snapshots of the congested road segments during the recovery stage at 7:50, 8:20, and 10:40. The congestion component gradually broke down from its maximal size (light green dashed links) as the road segments become no longer congested.

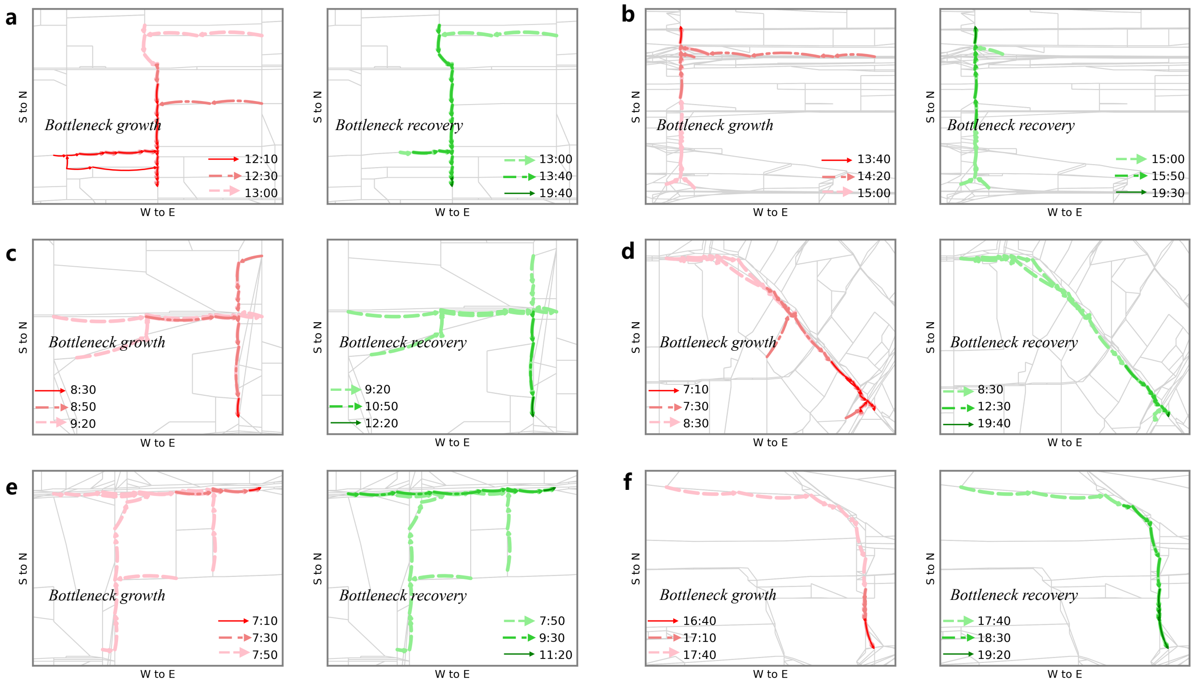

Fig. R4. Propagation and dissipation of congestion components associated with six typical bottlenecks, on Monday, October 12, 2015, in Beijing. The congestions originated at the bottleneck (dark red solid links), developed into more congestions (light red dashed links) in the upstream neighbouring streets during growth stage, and broke down from its maximal size (light green dashed links) as the road segments are no longer congested during recovery stage.

Comment R1C3: When defining S, the authors do not specify if there is a minimum length for the definition of a segment. The road network figures show some segments are too small to allow for a full arrow. Would the results change is some small segments were absorbed into the nodes?

Also, would other definitions of S that take into consideration not the number but the sum of the lengths of the road segments congested lead to any difference on the conclusions of the study?

Response: We thank the reviewer for this important comment. In the original manuscript, we did not specify a minimum length for the definition in the congestion size. We have now made new analysis by absorbing the very short roads or considering the congestion length, and found that the new results support robustness of our conclusions.

(1) When absorbing the very short roads into the nodes, we define the absorbed congestion size $S_a(t)$ of a jam component as the number of associated congested roads longer than or equal to 50 meters (Figs. **R5**a and b). The new results indicate that the patterns of the bottleneck dynamics and the performances of the predictions are robust.

(***i***) The distributions of growth duration and recovery duration identified by the absorbed size $S_a(t)$ (Figs. **R6**a and b) are very similar to those obtained by full size $S(t)$ defined in the manuscript (Figs. **R6**e and f). The stable exponents β of the growth duration (blue circles in Figs. **R7**a and b) and the recovery duration (blue circles in Figs. **R7**c and d), support our finding that scaling laws are regular in different days (orange triangles in Fig. **R7**).

(***ii***) The Pearson correlations $\rho_{S_P^a,V_A^a}$ between the maximal absorbed congestion size and the average growth speed in the new analysis (blue circles in Fig. **R8**) are slightly lower than the ρ_{S_P,V_A} in the manuscript (orange triangles in Fig. **R8**). This supports our finding that maximal jam size is highly and positively correlated to their growth speed.

(***iii***) The AUC values are around 0.95 for the predictions in the new analyses (blue circles in Fig. **R9**). This indicates the prediction results of the initial growth speed obtained by absorbed size, exhibit stable and good performance as in the manuscript (orange triangles in Fig. **R9**).

(2) When considering the sum of length as definition of S (Figs. **R5**b and c), the new results also indicate that the patterns of the bottleneck dynamics and the performances of the predictions are robust.

(***i***) The distributions of growth duration and recovery duration identified by the congestion length $S_l(t)$ (Figs. **R6**c and d) are very similar to those obtained by full size $S(t)$ defined in the manuscript (Figs. **R6**e and f). The stable exponents β of the growth duration (green triangles in Figs. **R7**a and b) and the recovery duration (green triangles in Figs. **R7**c and d),

support our finding that scaling laws are regular in different days (orange triangles in Fig. **R7**).

(***ii***) The Pearson correlations $\rho_{S_P^l,V_A^l}$ between the maximal congestion length and the average growth speed in the new analysis (green triangles in Fig. **R8**) are slightly higher than the ρ_{S_P,V_A} in the manuscript (orange triangles in Fig. **R8**). This supports our finding that maximal jam size is highly and positively correlated to their growth speed.

(***iii***) The AUC (the area under the ROC curve) values are around 0.95 for the predictions in the new analyses (green triangles in Fig. **R9**). This indicates the prediction results of the initial growth speed obtained by congestion length, exhibit stable and good performance as in the manuscript (orange triangles in Fig. **R9**). We have added all the above results in Supplementary Note 4, and Supplementary Figs. 13, 14, 15, 27 and 40 of the revised SI, and discussed them in Subsections 2.2, 2.3, and 2.5 of the revised Main Text.

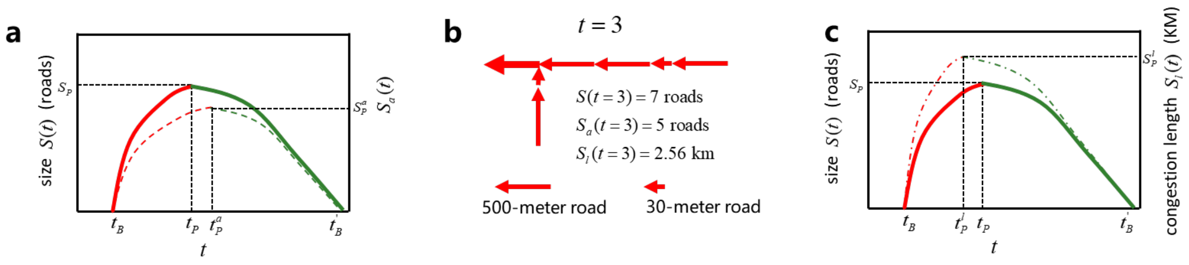

Fig. R5. Demos of the absorbed congestion size $S_a(t)$, congestion length $S_l(t)$, and size $S(t)$ in the manuscript. a and **c.** Demos of dynamics of (a) the absorbed size $S_a(t)$ and (c) the congestion length $S_l(t)$ compared to the size $S(t)$ in the manuscript. **b**. Three types of size S of the congestions associated with the given bottleneck at the selected time t=3. The congestion component has totally 7 road segments, including five 500-meter roads and two 30-meter roads.

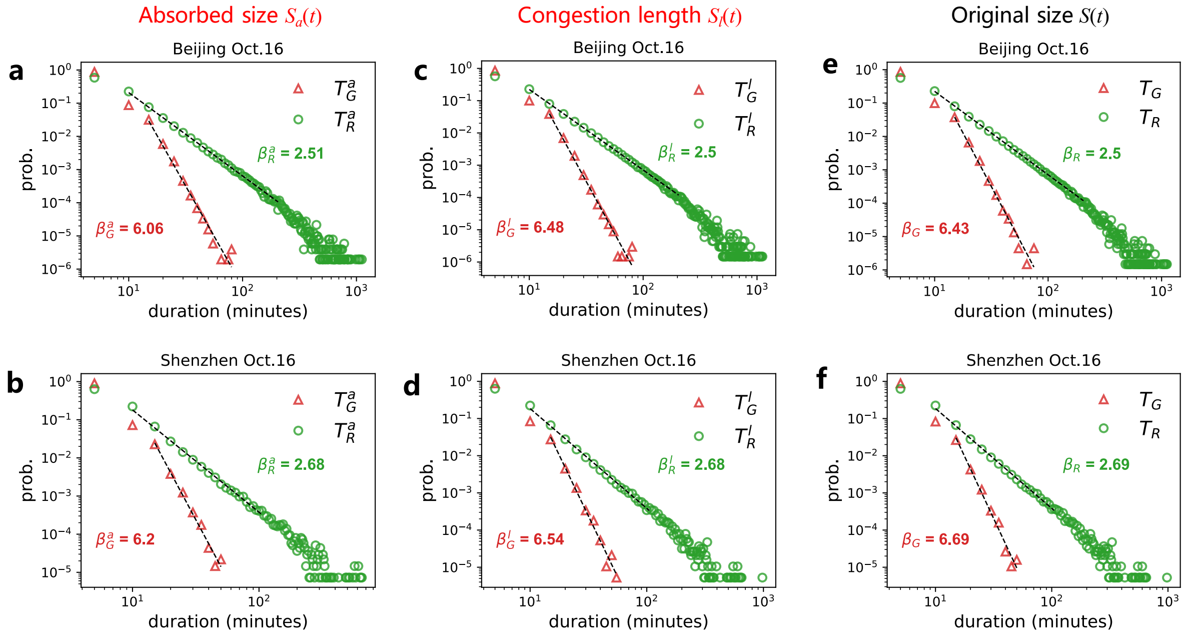

Fig. R6. Distributions of growth duration and recovery duration obtained by three definitions of congestion size. a and **b**. Distributions of the growth duration T_G^a and the recovery duration T_R^a identified based on the absorbed size $S_a(t)$ on October 16, Friday, 2015, for (a) Beijing and (b)

Shenzhen. **c** and **d**. Distributions of the growth duration T_G^l and the recovery duration T_R^l identified based on the congestion length $S_l(t)$ on October 16, Friday, 2015, for (c) Beijing and (d) Shenzhen. **e** and **f**. Distributions of the growth duration T_G and the recovery duration T_R identified based on the size $S(t)$ defined in the manuscript, on October 16, Friday, 2015, for (e) Beijing and (f) Shenzhen. *Here, the power-law distributions of growth duration* T_G *and recovery duration* T_R *are fitted in the ranges with the minimal values* $x_{\min} = 15$ *minutes and* $x_{\min} = 10$ *minutes respectively.*

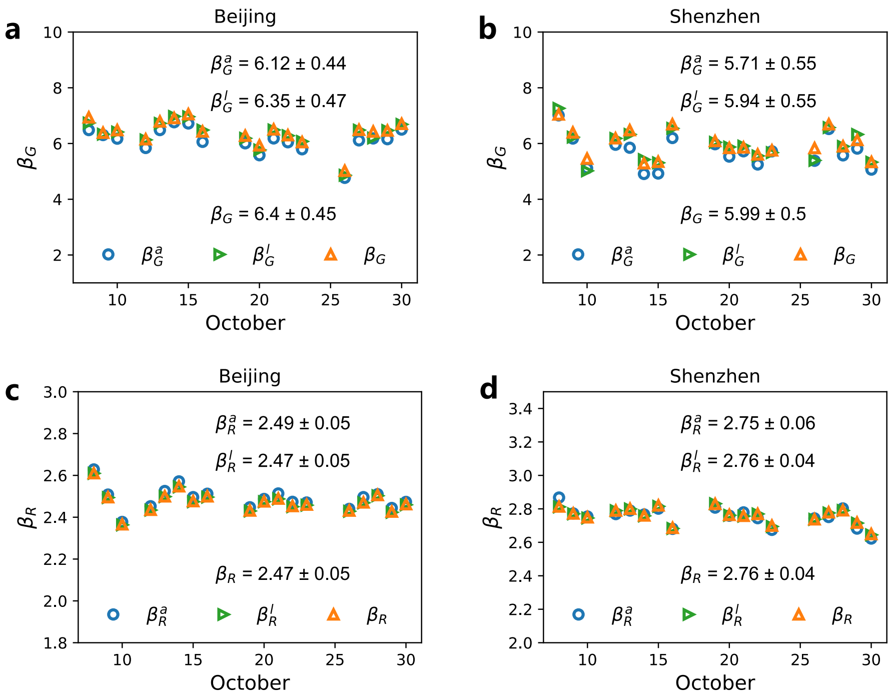

Fig. R7. Exponents of distributions of growth duration and recovery duration obtained by three definitions of congestion size, on 18 workdays in October, 2015. a and **b.** Exponents β_G^a, β_G^l, and β_G for the distributions of growth duration respectively obtained by the dynamics of absorbed size $S_a(t)$, congestion length $S_l(t)$ and size $S(t)$ in the manuscript, for 18 workdays in (a) Beijing and (b) Shenzhen. **c** and **d.** Exponents β_R^a, β_R^l, and β_R for the distributions of recovery duration respectively obtained by the dynamics of absorbed size $S_a(t)$, congestion length $S_l(t)$ and size $S(t)$ in the manuscript, for 18 workdays in (c) Beijing and (d) Shenzhen. *Here, the power-law distributions for growth duration* T_G *and recovery duration* T_R *are fitted in the ranges with the minimal values* $x_{\min} = 15$ *minutes and* $x_{\min} = 10$ *minutes respectively.*

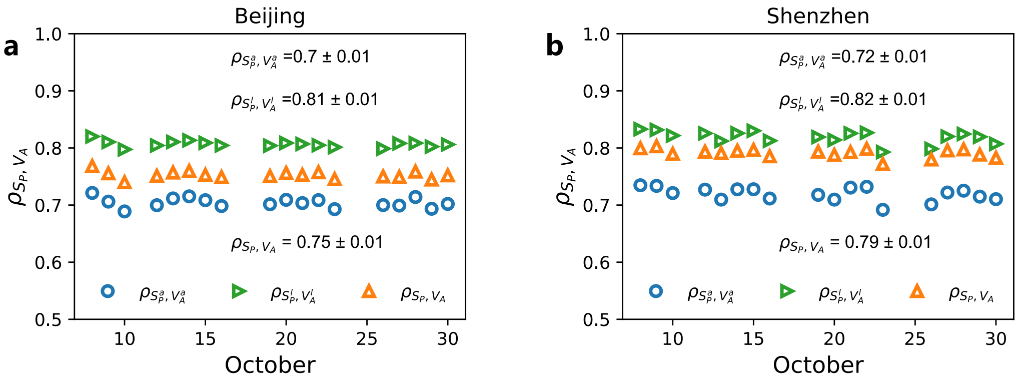

Fig. R8. Correlations between the spatial maximal size and the growth speed obtained by three definitions of congestion size, for 18 workdays in October, 2015, in (a) Beijing and (b) Shenzhen. Based on the absorbed size $S_a(t)$, we computed the average growth speed V_A^a as the ratio between the maximal absorbed size S_P^a and the growth duration T_G^a, where S_P^a is the number of the absorbed congested roads when the size $S_a(t)$ reaches maximum. Based on the congestion length $S_l(t)$, we also calculate the average growth speed V_A^l as the ratio between the maximal length S_P^l and the growth duration T_G^l, where S_p^l is the length of the congestion component when the length $S_l(t)$ (kilometers) reaches maximum. Here, V_A^l means the average increased length (kilometers) of congested road segments per 5 minutes. The correlation $\rho_{S_P^a,V_A^a}$ is the Pearson correlation between the absorbed size S_P^a and their average growth speed V_A^a. The correlation $\rho_{S_P^l,V_A^l}$ is the Pearson correlation between the congestion length S_P^l and its average growth speed V_A^l. The correlation ρ_{S_P,V_A} is the Pearson correlation between the maximal congestion sizes S_P and their average growth speed V_A in the manuscript.

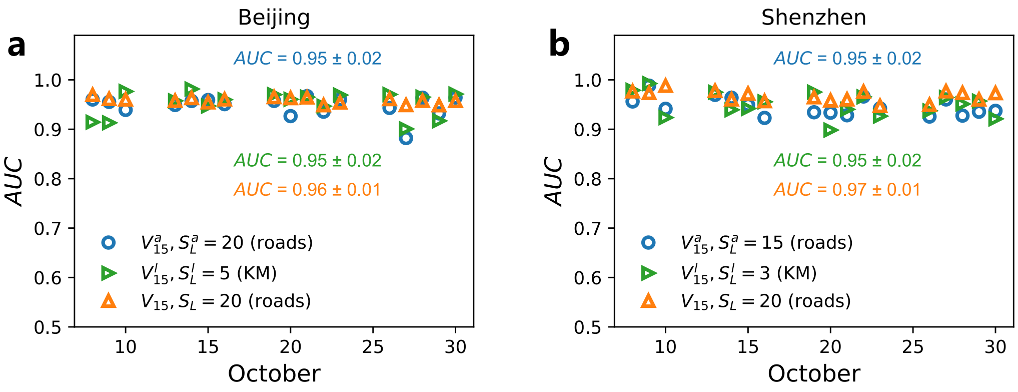

Fig. R9. Prediction performances based on the initial growth speed obtained by three definitions of congestion size, on 17 workdays for Oct. 2015, in (a) Beijing and (b) Shenzhen. The prediction models are trained based on the dynamics of bottlenecks on Oct. 12, Monday, 2015. For the definition of absorbed size $S_a(t)$, we calculate the initial growth speed $V_{T_I}^a$ as the number of the absorbed congested roads at the initial growth duration T_I (e.g., 15 minutes) divided by duration T_I, and applied initial growth speed V_{15}^a at the first 15 minutes to predict whether an absorbed congestion component would develop to be larger than or equal to the threshold size $S_L^a = 20$ (Beijing) or $S_L^a = 15$ (Shenzhen). For the definition of congestion length $S_L^l(t)$, we computed the initial growth speed $V_{T_I}^l$ of the congestions as the length of the congestions at the initial growth duration T_I divided by the duration T_I, and applied the initial growth speed V_{15}^l at the first 15 minutes to predict whether the length of the congestions associated with a bottleneck would develop to be longer than or equal to S_L^l =5 KM (Beijing) or S_L^l =3 KM (Shenzhen). For the definition of congestion size $S(t)$ in the manuscript, we use initial growth speed V_{15} at the first 15 minutes to predict whether the congestions associated with a bottleneck would develop to be larger than or equal to $S_L = 20$. The AUC (the area under the ROC curve) values are around 0.95 for the predictions in three definitions of *S*, which supports the robustness of the prediction performances.

Comment R1C4:

R1C4.1. I do not understand the results of Figs. 3 and 4. Equation (7) states that V_A is proportional to S_p. So, of course the two variables would be correlated!

The only thing that could alter that correlation is if T_G is also a function of S_p, in which case studying the relationship between S_p and T_G would be the thing to do...

Response: We thank the reviewer for this important comment. Following the suggestion of the reviewer, we analyzed the relationship between T_G and S_P, and found indeed that these two parameters are correlated. It is shown in Fig. **R10** that the growth duration T_G increases on average with S_P showing large fluctuations. The Pearson correlation between S_P and T_G is 0.3 for Beijing and 0.28 for Shenzhen. We have added the above analysis in Subsection 2.3 of the revised Main Text, and added Fig. **R10** in Supplementary Fig. 26 of the revised SI.

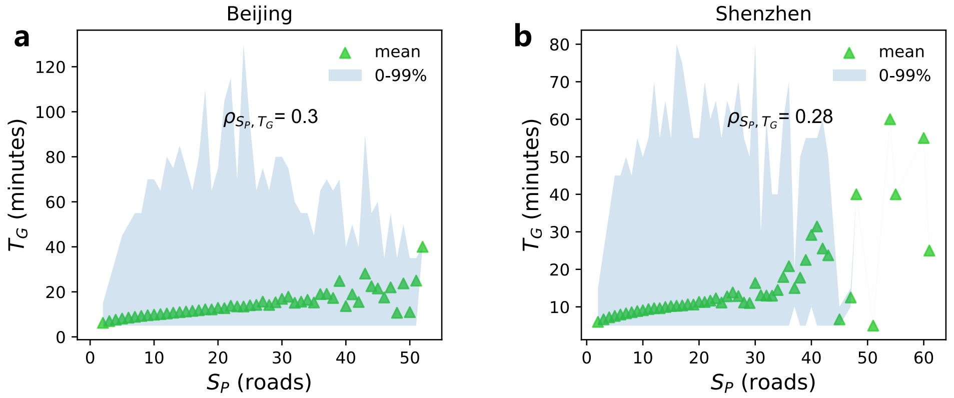

Fig. R10. Relationship of growth duration T_G versus maximal size S_P on 18 workdays in Oct., 2015, in (a) Beijing and (b) Shenzhen.

R1C4.2. Also, I really do not understand what is the point of showing box plots. I suggest that the authors plot density surfaces instead since they provide more information.

Response: We thank the reviewer very much for this good comment. We have calculated density surfaces of average growth speed V_A and size S_P, and found V_A and S_P are positively correlated. As shown in Fig. **R11**, the congestion components with a larger average growth speed V_A, are more likely to reach a larger congestion size S_P, consistent with our conclusion in the original manuscript. We have discussed the above results in Subsection 2.3 of the revised Main Text, and added the figures in Supplementary Fig. 23 of the revised SI.

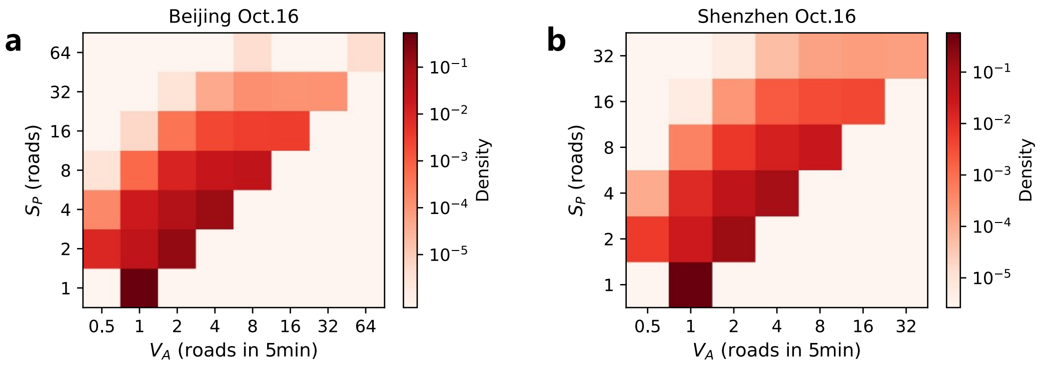

Fig. R11. Density surfaces of the average growth speed V_A and the maximal size S_P on Oct. 16, 2015, Friday, for (a) Bejing and (b) Shenzhen. The x-axis is the average growth speed V_A, the y-axis is the maximal size S_P, and the color bar represents the density of the observations.

Comment R1C5: Captions of Figures in SI do not provide full information to interpret them.

Response: We thank the reviewer very much for this important comment. We have carefully reorganized all the captions of the figures in the revised SI and provided full information to clarify them.

Comments R1C6: Data in Fig. S4c suggests that some cases of multiple DTBs could be due to data issues... This would affect the distributions of durations of growth and dissipation.

Response: We thank the reviewer very much for this comment. The cases of multiple DTBs in Fig. S4c mentioned by the reviewer, are regarded as separated jams. This is the result of our definition that a bottleneck comes to an end if the congested component associated with it completely dissipate. Therefore, if the congested state occurs in the same road again given the following high traffic flows, it is regarded as a new bottleneck. Note that the number of DTBs is actually related to the resolution of velocity data. We now compare the distributions of growth or recovery durations by smoothing the velocity and updating traffic in different resolution time intervals, i.e., every 5 and every 10 minutes. Our results show that bottlenecks on Oct. 16, in Beijing decreased to 195,000 by smoothing in 10 minutes, accounting for 30% of the bottlenecks (670,000) when smoothing every 5 minutes. This is because some of the multiple bottlenecks become now a single bottleneck. However, note that in Fig. **R12**, it is shown that both the exponent β_G (Fig. **R12**a) and exponent β_R (Fig. **R12**b) obtained when smoothing every 10 minutes, are very close to the values obtained when smoothing every 5 minutes. We have discussed the above results in Subsection 2.2 of the revised Main Text, and added these figures in Supplementary Fig. 16 of the revised SI.

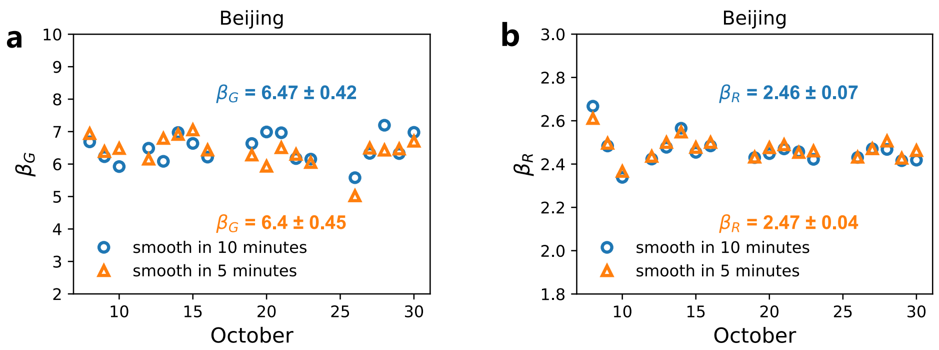

Fig. R12. Exponents of (a) β_G for the power-law distributions of growth duration, and (b) β_R for the power-law distributions of recovery duration, respectively by smoothing velocity records in 10 minutes and 5 minutes, in 18 workdays of Beijing. The distributions for growth duration T_G and recovery duration T_R are respectively fitted in the ranges with the minimal values $x_{\min} = 15$ minutes and $x_{\min} = 10$ minutes.

MAJOR CONCERNS:

Comments R1C7: I am concerned that neither the data nor the code used in this study are being posted on public sites. Research has shown that statements about 'sharing data or code upon request' are rarely followed through. Code should be posted to a public GitHub repository and data should be posted to some public repository that assigns a DOI.

Response: We thank the reviewer very much for this important suggestion. We have posted the code and necessary data in GitHub to enable the reproducibility of our findings (https://github.com/JinxiaoDuan/DynamicsOfBottlencks). As law regulations enacted in 2021 (http://www.cac.gov.cn/2021-08/20/c_1631049984897667.htm) by the Cyberspace Administration of China and the Ministry of Public Security of the People's Republic of China, the traffic flow or velocity data are not legally allowed to open. However, we provided the necessary datasets of bottleneck dynamics from the entire network to support the reproducibility of the paper. We have also included the GitHub links of the code and datasets in the sections "Code availability" and "Data Availability" in the revised manuscript.

Comments R1C8:

R1C8.1. The results of Figs. 2 and S5-S8 are quite unconvincing. I am surprised by a couple of things, first, that the authors do no plot the survival function instead of the probability density.

Response: We thank the reviewer for this good suggestion. We have plotted the survival functions and found they support our conclusions in the manuscript. In Fig. **R13**, $ccdf_{T_G}(t)$, $ccdf_{T_R}(t)$, and $ccdf_r(r_L)$ are respectively the survival functions (or complementary cumulative distribution functions, CCDFs) of the growth duration T_G, the recovery duration T_R, and ratio r between T_R and T_G. It is shown that they are approximately power law distributions with strong finite size effect, and the CCDFs of the recovery duration are significantly broader than those for the growth duration, which supports our conclusions in the manuscript. In Table R1, as expected, it is shown that $\beta_{ccdf_{T_G}}$, $\beta_{ccdf_{T_R}}$, and β_{ccdf_r} of CCDFs are respectively smaller by, close to 1, than the exponents β_G, β_R and β_r of the corresponding probability functions, due to the integration. We have added these survival functions in Supplementary Note 7, Supplementary Fig. 18 and Supplementary Table 1 of the revised SI, and discussed them in Subsection 2.2 of the revised Main text.

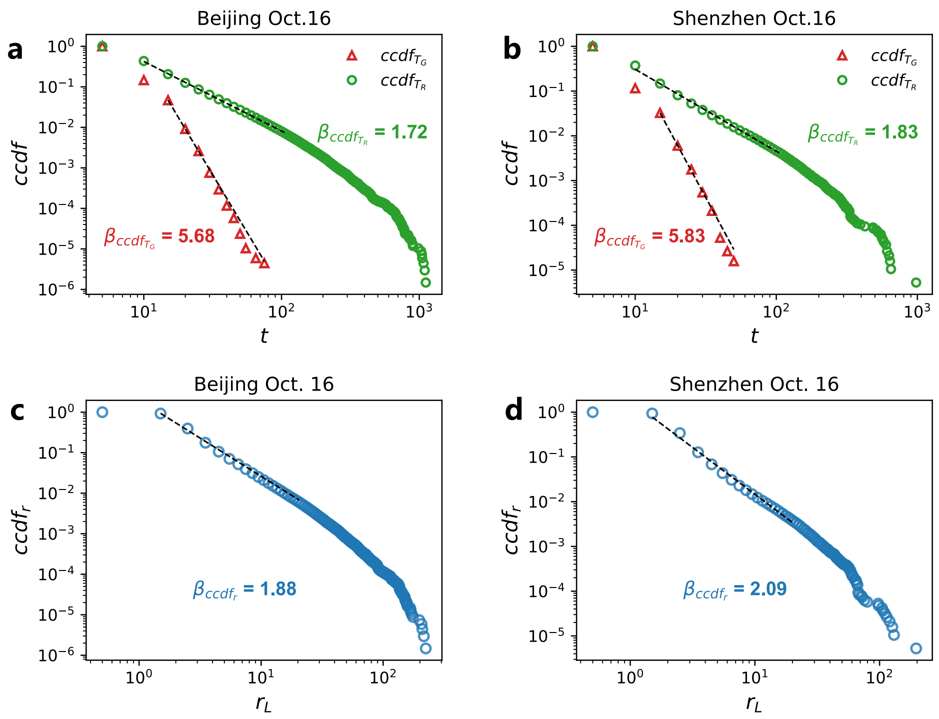

Fig. R13. Survival functions $ccdf_{T_G}$ of the growth duration T_G, $ccdf_{T_R}$ of the recovery duration T_R, and $ccdf_r$ of the ratio *r* between recovery duration and growth duration. a and **b.** Survival functions $ccdf_{T_G}$ and $ccdf_{T_R}$ on Oct. 16, 2015, Friday, in (a) Beijing and (b) Shenzhen. **c** and **d.** Survival functions of $ccdf_r$ on Oct. 16, 2015, Friday, in (c) Beijing and (d) Shenzhen. The distributions for $ccdf_{T_G}$, $ccdf_{T_R}$, and $ccdf_r$ are respectively fitted in the ranges with the minimal values $x_{\min}=15$ minutes, $x_{\min}=10$ minutes, and $x_{\min}=2$.

Table R1. Exponents of power law distributions for fitting survival functions ($\beta_{ccdf_{T_G}}$, $\beta_{ccdf_{T_R}}$ and β_{ccdf_r}) and probability functions (β_G, β_R and β_r) of the growth duration T_G, recovery duration T_R, and ratio *r* between T_R and T_G. The distributions for T_G, T_R and ratio *r* are respectively fitted in the ranges with the minimal values above $x_{\min}=15$ minutes, $x_{\min}=10$ minutes, and $x_{\min}=2$.

	**Growth duration T_G ($x_{\min}=15$)**		**Recovery duration T_R ($x_{\min}=10$)**		**Ratio *r* ($x_{\min}=2$)**	
	$\beta_{ccdf_{T_G}}$	β_G	$\beta_{ccdf_{T_R}}$	β_R	β_{ccdf_r}	β_r
Beijing	5.68	6.43	1.72	2.5	1.88	2.74
Shenzhen	5.83	6.69	1.83	2.69	2.09	2.96

R1C8.2. Second, that they do not appear to have performed any model selection analysis (Clauset, Shalizi and Newman have actually published a Python package that makes such an analysis quite easy to implement).

Response: We thank the reviewer for this excellent suggestion. We have added the model selection analysis to compare the power-law and the exponential models, and found that T_R, T_G and the ratio *r* are closer to power law distributions. As shown in Table R2, values of the log-likelihood ratio $\Re$ (Clauset et al., 2009) between the power law distribution $p_1(x)\sim x^{-\beta}$ and the exponential distribution $p_2(x)=\lambda e^{-\lambda x}$ are all positive. These values could suggest that

growth duration T_G, recovery duration T_R and the ratio r between T_R and T_G are all closer to power law distributions compared with exponential distributions. Although less probable, an exponential distribution cannot be fully excluded. We discuss more details about the two distributions for growth durations in next sub-response. We have added the above model selection analysis in Supplementary Note 8 and Supplementary Table 2 of the revised SI, and discussed them in Subsection 2.2 of the revised Main Text.

Table R2. The log-likelihood ratio $\Re$ between the power law and the exponential distributions for growth duration, recovery duration and the ratio r between recovery and growth durations, on Oct. 16, 2015 in Beijing and Shenzhen. The values of the log-likelihood ratio are normalized here. The value of $\Re$ is positive if the distribution of the empirical data is closer to a power law model, and negative if it is closer to an exponential model (Clauset et al., 2009). The distributions for T_G, T_R and ratio r are respectively fitted in the ranges with the minimal values above $x_{\min}=15$ minutes, $x_{\min}=10$ minutes, and $x_{\min}=2$.

	**Growth duration T_G** ($x_{\min}=15$)	**Recovery duration T_R** ($x_{\min}=10$)	**Ratio r** ($x_{\min}=2$)
Beijing	69.65	242	68.45
Shenzhen	36.40	122.33	30.94

R1C8.3. Third, the power law fit to T_G is utterly unconvincing. The data is clearly convex and the exponent estimate is very larger, suggesting that an exponential functional form is likely a much better description of the data.

Response: We thank the reviewer very much for bringing this good comment to our attention. We have tested the goodness of exponential and power-law models for fitting growth duration T_G in different ranges, and found they are respectively plausible for $T_G \geq 10$ minutes and $T_G \geq 15$ minutes.

When fitting growth duration T_G above $x_{\min}=10$ minutes, the *p*-values of the Kolmogorov-Smirnov KS measure (Clauset et al., 2009) in Table R3 are lower than 0.1 for the power-law models, and slightly higher than 0.1 for the exponential models. This could suggest the growth durations T_G are better fitted by an *exponential distribution* (Fig. **R14**) rather than power-law model for $T_G \geq 10$ minutes. Whereas, when fitting T_G above $x_{\min}=15$ minutes, the *p*-values are 0.976 (Beijing) and 0.417 (Shenzhen) for power-law models, while they are smaller than 0.1 for the exponential models. This could suggest the growth durations are more suited to a *power-law distribution* (Fig. **R15**) for $T_G \geq 15$ minutes.

It is suggested above that the sensitivity of the distributions is mainly affected by small values of T_G. Therefore, when focusing on the long-lifetime congestions at the tail of distribution, features of power-law do exist. However, we must state here that the distribution type of growth duration does not affect our main results, including the prediction performances in our manuscript. We have added these results in Supplementary Note 8, Supplementary Figs. 19 and 20, and Supplementary Table 3 of the revised SI, and discussed them in Subsection 2.2 of the revised Main Text.

Table R3. The *p*-values of the power-law $p_1(x) \sim x^{-\beta}$ and exponential $p_2(x) = \lambda e^{-\lambda x}$ distributions for the growth duration T_G. We conducted 1000 simulations and calculated the *p*-value as the

proportion of that with the synthetic distances larger than the empirical distance to the estimated curve (Clauset et al., 2009). If p is large (commonly larger than 0.1), the distance between the empirical data and the estimated curve can be considered as statistical fluctuations; if it is smaller than 0.1, the model may not be suitable to fit the empirical data.

	$x_{min} = 10$		$x_{min} = 15$	
	Power law	Exponential	Power law	Exponential
Beijing	0.003 ($\beta_G = 5.94$)	0.126 ($\lambda_G = 0.25$)	0.976 ($\beta_G = 6.43$)	0.006 ($\lambda_G = 0.23$)
Shenzhen	0.018 ($\beta_G = 5.96$)	0.191 ($\lambda_G = 0.28$)	0.417 ($\beta_G = 6.69$)	0.077 ($\lambda_G = 0.26$)

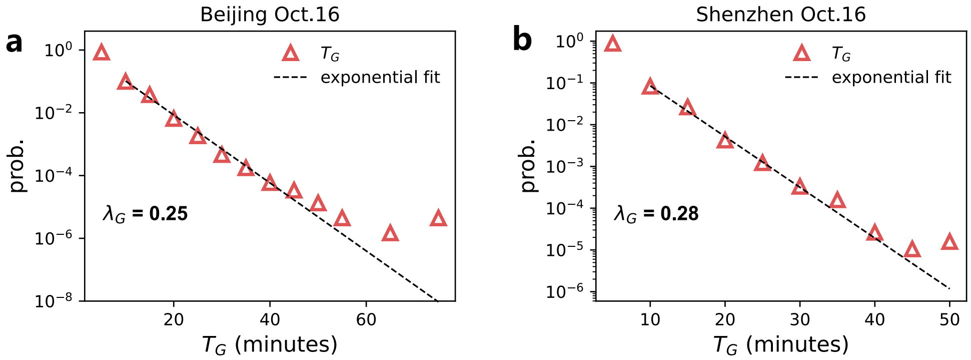

Fig. R14. Distributions of the growth duration T_G fitted by exponential models in the range above $x_{min} = 10$ minutes on Oct. 16, 2015 in (a) Beijing and (b) Shenzhen.

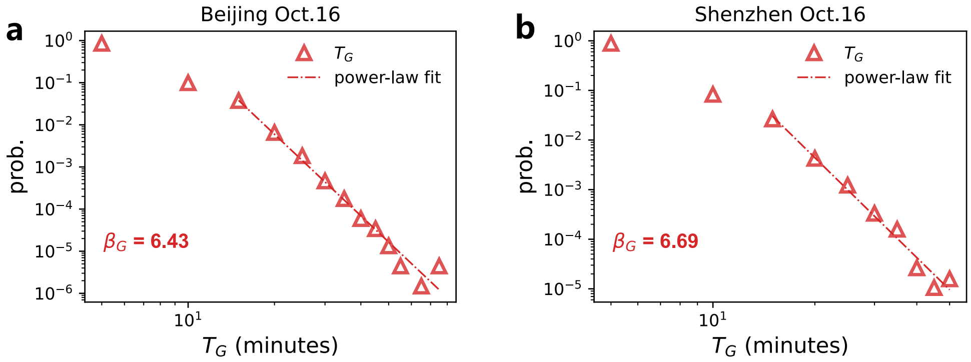

Fig. R15. Distributions of the growth duration T_G fitted by power-law models in the range above $x_{min} = 15$ minutes on Oct. 16, 2015 in (a) Beijing and (b) Shenzhen.

References:

Clauset, A., Shalizi, C.R., Newman, M.E. 2009. Power-law distributions in empirical data. SIAM review 51 (4), 661-703.

Comments R1C9: I find that the authors do not provide compelling evidence for the lack of recurrence of the heavy congestion events. One would not expect large recurrence when comparing rush versus non-rush hours, but would expect large recurrence when comparing rush hour in different workdays. Moreover, the low Jaccard index for bottlenecks associated with large congestions is not surprising, since one could expect the actual starting road segment to possibly vary within some close set of road segments.

I think it would be important to consider other definitions of overlap between congestion events so that one can be convinced of the robustness of the conclusions. I also thing that the manuscript could benefit from some diagrams in the SI showing how overlap is calculated.

Response: We thank the reviewer very much for this helpful suggestion. We have considered a new definition of overlap and found the new results support the robustness of our conclusions. As the reviewer kindly suggested, we relaxed the definition of overlap, and regarded bottlenecks on different days as overlap *either* if they emerge on the same road, *or* if one is on the nearest upstream or downstream road segment of another (see the diagrams in Fig. **R16**b).

The Jaccard indicator J_B is now defined as the ratio between the size of the "relaxed overlap" and the size of the union of the bottlenecks in two different days. It is shown in Fig. **R17** that, indeed, the Jaccard values of the relaxed overlap are significantly larger than the original strict overlap. Especially, the relaxed J_B is very close to 1 for the jam components with maximal size S_P larger than threshold $S_L = 1$, meaning that almost all streets *can be or close* to a bottleneck of a jam with size $\geqslant 1$.

Nevertheless, the relaxed Jaccard index J_B is interestingly only 5.82% in rush hours (Fig. **R17**a) and 4.52% in non-rush hours (Fig. **R17**b) for large congestion components with size $\geqslant 20$ roads. This indicates that the large congestions are less recurrent even in the relaxed analysis, consistent with our conclusions in the original manuscript. The low similarity of large congestion events could be due to that the traffic flows are the result of the dynamic self-organization of the game behaviors of numerous travelers (Roughgarden, 2005; Çolak et al., 2016; Schröder et al., 2020). In the day-by-day game process, due to risk aversion, travelers have been found to make adaptive choices and deviate from the congestions in previous days (Cantarella and Cascetta, 1995; Dussutour et al., 2004; Yeung et al., 2013; Vazifeh et al., 2018). Thus, large congestion events could be less recurrent than expected.

We have added the diagrams of the two definitions of overlap in Supplementary Fig. 29, and added the new analysis in Supplementary Note 10 and Supplementary Fig. 33 of the revised SI. We have also discussed them in the Subsection 2.5 of the revised Main text.

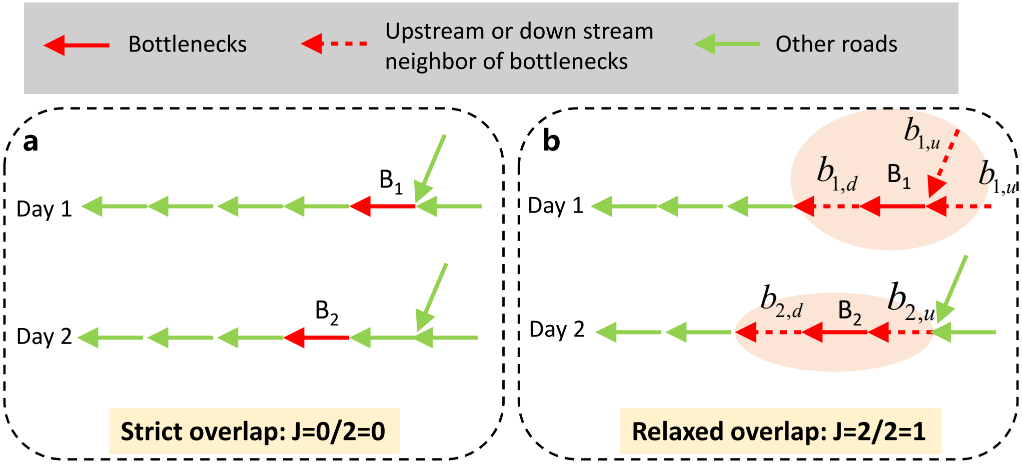

Fig. R16. Demonstration of overlap calculations by the original strict definition and the new relaxed definition. a. By the strict definition, bottleneck B_1 on Day1 does not overlap with

bottleneck B_2 on Day2, since the strict definition of overlap (in the original manuscript) regards two bottlenecks being overlapped only if they emerge in the same road segment. **b**. By the relaxed definition, bottleneck B_1 on Day1 is regarded as overlapping with bottleneck B_2 on Day2, since the relaxed definition considers bottlenecks that emerged on 1-step nearby roads also overlapped. $b_{i,u}$ and $b_{i,d}$ are the 1-step upstream and downstream roads of the bottleneck B_i ($i = 1,2$).

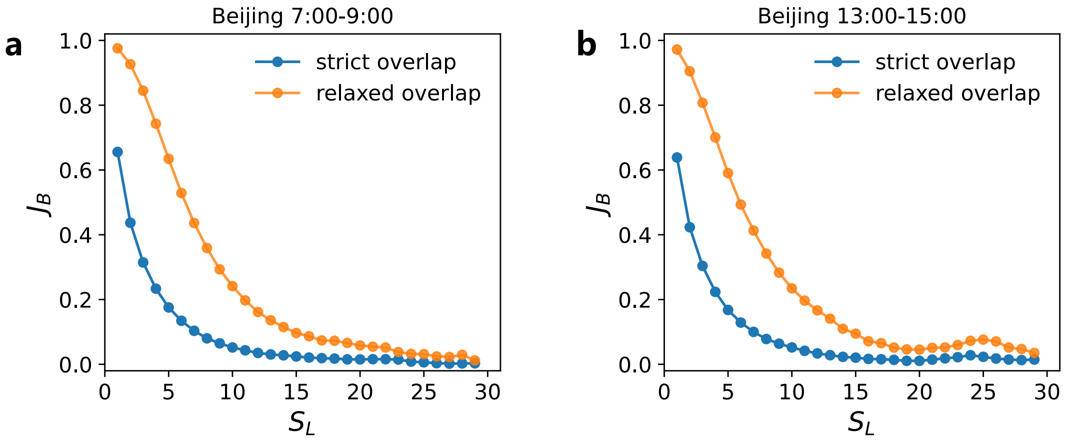

Fig. R17. Jaccard indexes J_B of the relaxed and strict overlaps for bottlenecks under different threshold size S_L (i.e., bottlenecks with size $S_P \geq S_L$) during (a) rush hours and (b) non-rush hours among different days. We calculated the Jaccard indexes J_B for the bottlenecks between every two of 5 workdays (i.e., Oct. 12, 13, 14, 15 and 16, 2015, in Beijing) and average them.

References:

Cantarella, G.E., Cascetta, E. 1995. Dynamic processes and equilibrium in transportation networks: towards a unifying theory. Transportation Science 29 (4), 305-329.

Çolak, S., Lima, A., González, M.C. 2016. Understanding congested travel in urban areas. Nature Communications 7, 10793.

Dussutour, A., Fourcassié, V., Helbing, D., Deneubourg, J.-L. 2004. Optimal traffic organization in ants under crowded conditions. Nature 428 (6978), 70-73.

Roughgarden, T. 2005. Selfish routing and the price of anarchy. MIT press.

Schröder, M., Storch, D.-M., Marszal, P., Timme, M. 2020. Anomalous supply shortages from dynamic pricing in on-demand mobility. Nature communications 11 (1), 4831.

Vazifeh, M.M., Santi, P., Resta, G., Strogatz, S.H., Ratti, C. 2018. Addressing the minimum fleet problem in on-demand urban mobility. Nature 557 (7706), 534-538.

Yeung, C.H., Saad, D., Wong, K.M. 2013. From the physics of interacting polymers to optimizing routes on the London Underground. Proceedings of the National Academy of Sciences 110 (34), 13717-13722.

Reviewer #2 (Remarks to the Author):

Overall, the research is interesting, novel and a contribution to the body of scientific body of knowledge on traffic science. The developed approach examines traffic congestion on a highly aggregate scale but in quite creative ways.

Response: We thank the reviewer very much for the positive and important comments, and for considering our research "interesting, novel" and "in quite creative ways". We have carefully considered all the comments and revised the manuscript along with the good suggestions of the reviewer. Below we provide the point-by-point responses.

While the work is of value to traffic science, the motivation related to signalization could benefit from a bit further coverage given the significant range of research which has been done related to real-time monitoring for traffic control.

Response: We thank the reviewer very much for this valuable suggestion. We have highlighted our research motivation with respect to real-time urban traffic control. As a major approach of urban traffic control, advanced real-time monitoring technologies have been found effective in helping avoid traffic jams (Webster, 1958; Priemer and Friedrich, 2009; Inoue et al., 2021). The early real-time traffic control began with the implementation of the traffic signal control system SCOOT to calculate the size of the queue and time of clearance given the cyclic flow profiles (Robertson and Bretherton, 1991). With artificial intelligence technologies, the model predictive control (MPC) framework has been proposed to sense and control traffic conditions while agents interact with ones in the vicinity (Srinivasan et al., 2006; De Oliveira and Camponogara, 2010; Thunig et al., 2019). Meanwhile, connected automated vehicles (CAVs) techniques have also been considered to improve the predictive control performances of urban signalized intersections (Guo et al., 2019; Feng et al., 2023).

These control technologies are excellent in considering time-oriented prediction, while limited focuses have been directed at controlling spatial congestion propagation. Meantime, the spatial congestion propagation could significantly influence the coordinative control performances among different signalized intersections (Hegyi, 2004; Ramezani et al., 2017; Barmpounakis and Geroliminis, 2020). Based on our identified spatiotemporal propagation mechanisms, the real-time control systems could consider interrelations among different traffic regions and perform better coordinated control for the most congested areas before they propagate to a global gridlock jam. We have highlighted our motivation related to signalization and cited the important references in the Discussion of the revised Main Text.

References:

Barmpounakis, E., Geroliminis, N. 2020. On the new era of urban traffic monitoring with massive drone data: The pNEUMA large-scale field experiment. Transportation research part C: emerging technologies 111, 50-71.

De Oliveira, L.B., Camponogara, E. 2010. Multi-agent model predictive control of signaling split in urban traffic networks. Transportation Research Part C: Emerging Technologies 18 (1), 120-139.

Feng, S., Sun, H., Yan, X., Zhu, H., Zou, Z., Shen, S., Liu, H.X. 2023. Dense reinforcement learning for safety validation of autonomous vehicles. Nature 615 (7953), 620-627.

Guo, Q., Li, L., Ban, X.J. 2019. Urban traffic signal control with connected and automated vehicles: A survey. Transportation research part C: emerging technologies 101, 313-334.
Hegyi, A. 2004. Model predictive control for integrating traffic control measures. Netherlands TRAIL Research School.
Inoue, D., Okada, A., Matsumori, T., Aihara, K., Yoshida, H. 2021. Traffic signal optimization on a square lattice with quantum annealing. Scientific reports 11 (1), 1-12.
Priemer, C., Friedrich, B. 2009. A decentralized adaptive traffic signal control using V2I communication data. Proceedings of 2009 12th international ieee conference on intelligent transportation systems, pp. 1-6.
Ramezani, M., de Lamberterie, N., Skabardonis, A., Geroliminis, N. 2017. A link partitioning approach for real-time control of queue spillbacks on congested arterials. Transportmetrica B: transport dynamics 5 (2), 177-190.
Robertson, D.I., Bretherton, R.D. 1991. Optimizing networks of traffic signals in real time-the SCOOT method. IEEE Transactions on vehicular technology 40 (1), 11-15.
Schröder, M., Storch, D.-M., Marszal, P., Timme, M. 2020. Anomalous supply shortages from dynamic pricing in on-demand mobility. Nature communications 11 (1), 4831.
Srinivasan, D., Choy, M.C., Cheu, R.L. 2006. Neural networks for real-time traffic signal control. IEEE Transactions on intelligent transportation systems 7 (3), 261-272.
Thunig, T., Kühnel, N., Nagel, K. 2019. Adaptive traffic signal control for real-world scenarios in agent-based transport simulations. Transportation Research Procedia 37, 481-488.
Vazifeh, M.M., Santi, P., Resta, G., Strogatz, S.H., Ratti, C. 2018. Addressing the minimum fleet problem in on-demand urban mobility. Nature 557 (7706), 534-538.
Webster, F.V. 1958 Traffic signal settings. Road Research Laboratory, London, U.K., Road Res. Tech. 39.
Yeung, C.H., Saad, D., Wong, K.M. 2013. From the physics of interacting polymers to optimizing routes on the London Underground. Proceedings of the National Academy of Sciences 110 (34), 13717-13722.

Specific Comments:

Comments R2C1: As per your definition S(t) is the number of congested links – but, by integrating the difference in the number of congested and dissipated links over time would this not give you the duration of overall congestion and not the number of congested links? (Eq -3)

a. Same with eq-4 in the supplementary text.

Response: We thank the reviewer very much for this important question. We have added a demo to improve the explanation of the equation. In Fig. **R18**a, it is shown that the "*number of congested links*" becomes from $S(t=2)=3$ at time *t*=2, to $S(t=3)=4$ at time *t*=3 by integrating the difference between the number of newly developed links $G(t=3)=2$ and newly recovered links $R(t=3)=1$, exactly as in

$$S(t)=\int_{t_B}^{t}\frac{dS(u)}{du}du=\int_{t_B}^{t}\left(G(u)-R(u)\right)du\,. \tag{R2}$$

In Fig. **R18**b, the overall dynamic process of the size $S(t)$ is displayed. In this process, the emergence time $t_B=0$, and the disappearing time $t_B^{'}=8$ are identified by $S(t)=0$. The growth stage ends and the recovery stage starts at time $t_P=3$, distinguished by the maximal size S_P=4. The duration of overall congestion is 8 time intervals. We have added the demo in Supplementary Note 3 and Supplementary Fig. 6 of the revised SI, and improved the explanations of Eq. (3) in Subsection 2.1 of the revised Main Text and Eq. (4) in the revised SI.

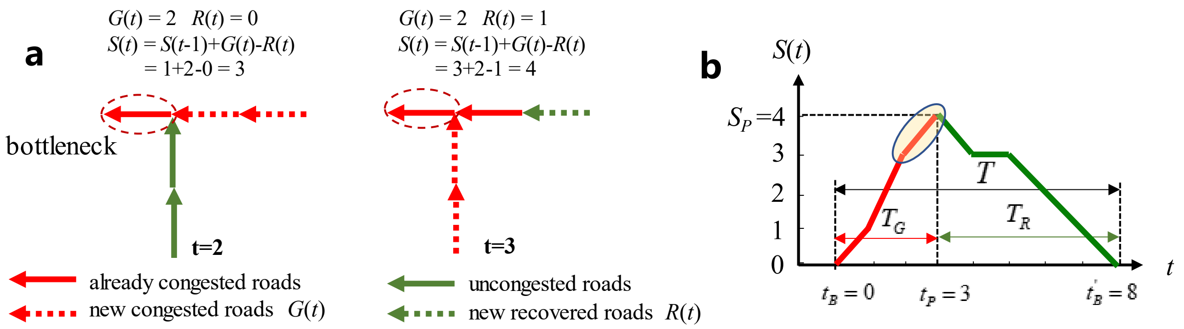

Fig. R18. Demonstration of dynamics of traffic bottleneck. a. Demonstration of congestion size S associated with a bottleneck. **b**. Demonstration of the congestion size dynamics.

Comments R2C2: It seems that generalizing the statement that dissipation takes twice more than the build-up could be further discussed. Since the article is studying the network level, not at a corridor. if the flow in one corridor is high for a prolonged period, it causes the dissipation to last longer, and it need not be because of the initial congestion bottleneck but can also be because of traffic control devices?

Response: We appreciate the reviewer's important comment. We assume that the longer dissipation may be related to traffic control devices, enhanced by the network's propagation mechanisms.

On one hand, studies have found that even though the application of traffic control devices can improve traffic operation, some roads may experience worse traffic and sustain longer congestion duration (Al-Madani, 2003; He et al., 2014; Le et al., 2015). For example, traffic control devices, such as the "red light" traffic signal, may block vehicles and cause higher traffic flow on some roads. On the other hand, congestions may spread to other roads, due to the network's propagation mechanisms from the bottleneck. For example, previous studies have described the congestion propagation process as the percolation procedure (Li et al., 2015; Hamedmoghadam et al., 2021), and found such process can accumulate fractional congestions into a global jam (Zeng et al., 2019; Zeng et al., 2020), so that it may take a long time to dissipate due to the propagation mechanism. These two mechanisms may interact with each other to produce the long dissipation we observed. We have added the above discussions in Supplementary Note 13 of the revised SI, and discussed them in the Discussion of the revised Main Text.

References:

Al-Madani, H.M. 2003. Dynamic vehicular delay comparison between a police-controlled roundabout and a traffic signal. Transportation Research Part A: Policy and Practice 37 (8), 681-688.

Hamedmoghadam, H., Jalili, M., Vu, H.L., Stone, L. 2021. Percolation of heterogeneous flows uncovers the bottlenecks of infrastructure networks. Nature communications 12 (1), 1254.

He, Q., Head, K.L., Ding, J. 2014. Multi-modal traffic signal control with priority, signal actuation and coordination. Transportation research part C: emerging technologies 46, 65-82.

Le, T., Kovács, P., Walton, N., Vu, H.L., Andrew, L.L., Hoogendoorn, S.S. 2015. Decentralized signal control for urban road networks. Transportation Research Part C: Emerging Technologies 58, 431-450.

Li, D., Fu, B., Wang, Y., Lu, G., Berezin, Y., Stanley, H.E., Havlin, S. 2015. Percolation transition in dynamical traffic network with evolving critical bottlenecks. Proceedings of the National Academy of Sciences 112 (3), 669-672.

Zeng, G., Gao, J., Shekhtman, L., Guo, S., Lv, W., Wu, J., Liu, H., Levy, O., Li, D., Gao, Z. 2020. Multiple metastable network states in urban traffic. Proceedings of the National Academy of Sciences of the United States of America 117 (30), 17528-17534.

Zeng, G., Li, D., Guo, S., Gao, L., Gao, Z., Stanley, H.E., Havlin, S. 2019. Switch between critical percolation modes in city traffic dynamics. Proceedings of the National Academy of Sciences of the United States of America 116 (1), 23-28.

Comments R2C3: Any reason why the beta's on the weekend is similar to a weekday and not a holiday or some other value?

Response: We thank the reviewer very much for this important question. The values of β are actually different in three types of days with different degrees of deviations. i.e., β_G and β_R on weekends are clearly lower than the values on holidays, whereas they are slightly higher than the values on weekdays, as shown in Fig. 2 in the manuscript.

The differences in exponent β could be due to traffic flow patterns. In megacities Beijing and Shenzhen, the weekdays experienced the heaviest traffic demands due to the great number of commuting trips for local residents. The higher demand possibly induces more high-flow roads and more long-duration congested roads, which could bring to large tails of the duration distributions and therefore smallest exponents. Regular weekends experienced lower traffic demands and different demand modes by partially leisure trips for local residents. However, the representative National holiday, as one of the largest holidays, experienced significantly reduced travel demand and significantly different demand patterns by tourists from all over the country. We have added the explanations for the different values of β in Supplementary Note 5 of the revised SI, and discussed them in Subsection 2.2 of the revised Main text.

Comments R2C4: Line 304- In traffic studies, it tends to be more common to check how the patterns are based on the day of the week not across days within a week.

Response: We thank the reviewer very much for this important suggestion. We have calculated the overlap of the congestions based on the days across different weeks, and the new results support our conclusions. In Fig. **R19**, we calculated the Jaccard indexes J_B for overlap of traffic bottlenecks and J_C for overlap of their associated congestions between every two of the four Fridays. It is shown that with the increasing threshold size S_L, both the similarities of the bottlenecks (Fig. **R19**a) and their associated congestions (Fig. **R19**b) decrease rapidly. Values of J_B and J_C at $S_L = 20$ indicate that only 5% of all the heavy bottlenecks and 20% of their associated congestions (i.e., $S_P \geq 20$) that appear in both Fridays are the same, consistent with our conclusion that the large congestion components (e.g., $S_L = 20$) are less recurrent. We have added the new results in Supplementary Fig. 31c and d of the revised SI, and discussed them in Subsection 2.5 of the Main Text.

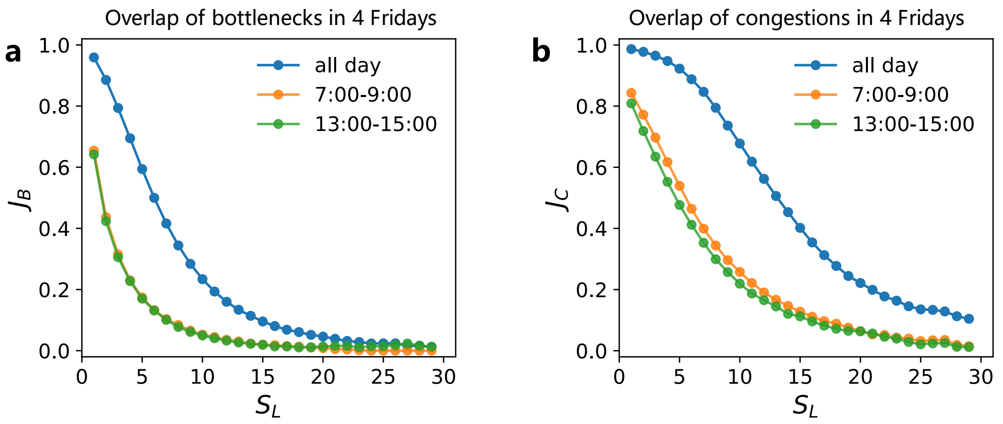

Fig. R19. Jaccard indexes J_B for bottlenecks and J_C for their associated congestion components for bottlenecks under different threshold size S_L (i.e., bottlenecks with size $S_P \geq S_L$). It is noted that each point is the mean value of the Jaccard index obtained between every two Fridays (Oct. 9, 16, 23, and 30, 2015) in 4 weeks, in Beijing. J_B is the ratio between the size of overlapped bottlenecks and the size of the union of bottlenecks on two different days. J_C is the ratio between the size of the overlap of the associated congestions and the size of the union of the associated congestions on two different days. **a** is the Jaccard index J_B of bottlenecks under different threshold size S_L for the bottlenecks in all day (0:00-24:00), rush hours (7:00 am-9:00 am) and non-rush hours (13:00 pm-15:00 pm), respectively. **b** is the Jaccard index J_C of the associated congestion components under different threshold size S_L. The curves show that repetition of both bottlenecks and their associated congestions decreases rapidly when S_L becomes larger, indicating that the major bottlenecks and their associated congested roads are rarely recurrent.

Comments R2C5: Line 105 – Should it be “emergence”, not “emergency”?

Response: We thank the reviewer very much for this kind reminder. We have systematically checked such errors and corrected them in the revised manuscript.

Comments R2C6: Some sentences might benefit from a bit further refinement – e.g., …we follow the lifecycle of each dynamics of traffic bottlenecks (DTB) in the entire road networks…

Response: We thank the reviewer very much for this kind note. We have systematically checked such sentences and refined them in the revised manuscript.

Comments R2C7: In supplementary figures 12 and 13, the trend is counter-intuitive, because larger congestions should take more time to dissipate than smaller congestion however all plots seem to show opposite trend. Could this be explained?

Response: We thank the reviewer very much for this important comment. It is shown in Supplementary Figs. 12 and 13 (Supplementary Figs. 24 and 25 in the current version) that large bottlenecks are less probable to sustain for a longer duration. This could be because

drivers are strongly averse to very large congestions in the self-organization process of their game behavior, and would like to make adaptive choices to deviate from the long queues (De Palma et al., 2012; Liu and Liu, 2018). Moreover, well-developed traffic control technologies could have also focused on alleviating these heavy congestions (Thunig et al., 2019). Thus, the heavy congestion would not sustain for a long time due to the quick alleviation of the traffic flow. We have added the above discussions in Subsection 2.3 of the revised Main text.

References:

De Palma, A., Lindsey, R., Picard, N. 2012. Risk aversion, the value of information, and traffic equilibrium. Transportation Science 46 (1), 1-26.

Liu, P., Liu, Y. 2018. Optimal information provision at bottleneck equilibrium with risk-averse travelers. Transportation Research Record 2672 (48), 69-78.

Thunig, T., Kühnel, N., Nagel, K. 2019. Adaptive traffic signal control for real-world scenarios in agent-based transport simulations. Transportation Research Procedia 37, 481-488.

Comments R2C8: The predicator analysis could benefit from a bit further attention:

R2C8.1. – To better understand the efficacy of the predictor, it is important to mention the overall congestion stats, i.e., a histogram of congestion size Sp to indicate the share of SL > 20 that are actually being predicted. Then, an appropriate mention of the actual time duration distributions of all such larger congestions.

Response: We deeply appreciate the reviewer's critical and excellent comments. We have plotted the complementary cumulative distribution function (CCDF) of jam size to calculate the share of large congestions, and plotted CCDF of actual time duration for the large congestion with size $S_P \geq 20$. In Fig. **R20**, the CCDF of size S_P on Oct. 16 shows that the number of bottlenecks with $S_P \geq 10$ and $S_P \geq 20$ are respectively 11,890 and 611, which accounts for 1.18% and 0.09% of all the bottlenecks. The fraction of the large congestions is small yet the most dangerous, and should be the most important yet missing control targets given their potential damages. In Fig. **R21**, the CCDF of jam duration T shows that the large congestions ($S_P \geq 20$) with jam duration $T \geq 15$ minutes, $T \geq 30$ minutes and $T \geq 60$ minutes, respectively account for 90%, 46% and 17% of all the large congestions. We have added these results in Supplementary Note 8, and Supplementary Figs. 35 and 36 of the revised SI, and discussed them in Subsection 2.5 of the revised Main Text.

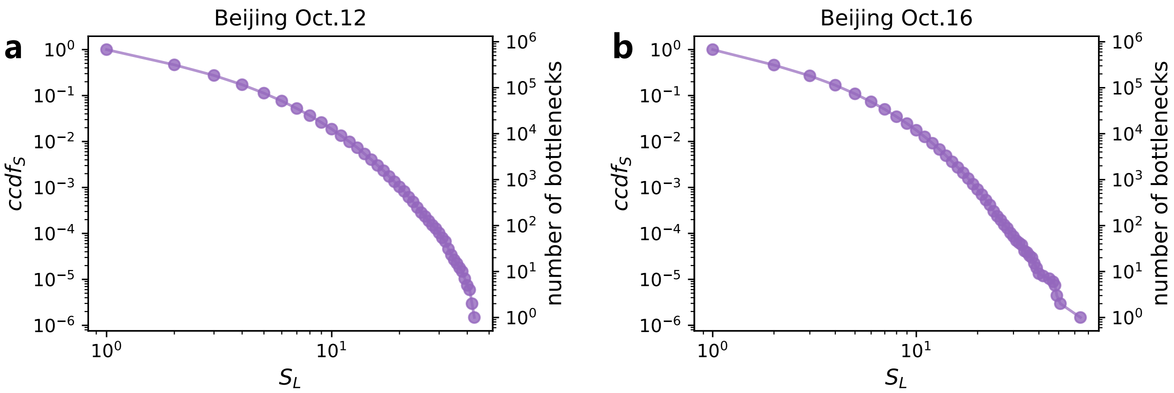

Fig. R20. Complementary cumulative distribution function of size S_P on (a) Oct. 12, Monday and (b) Oct.16, Friday, 2015, in Beijing. The x-axis is the threshold size S_L. The left y-axis, i.e., the CCDF of the congestion size S_P, represents the probability of the bottleneck with the maximal size S_P larger than or equal to the threshold size S_L. The right y-axis is the number of the bottlenecks with maximal size S_P larger than or equal to the given threshold size S_L.

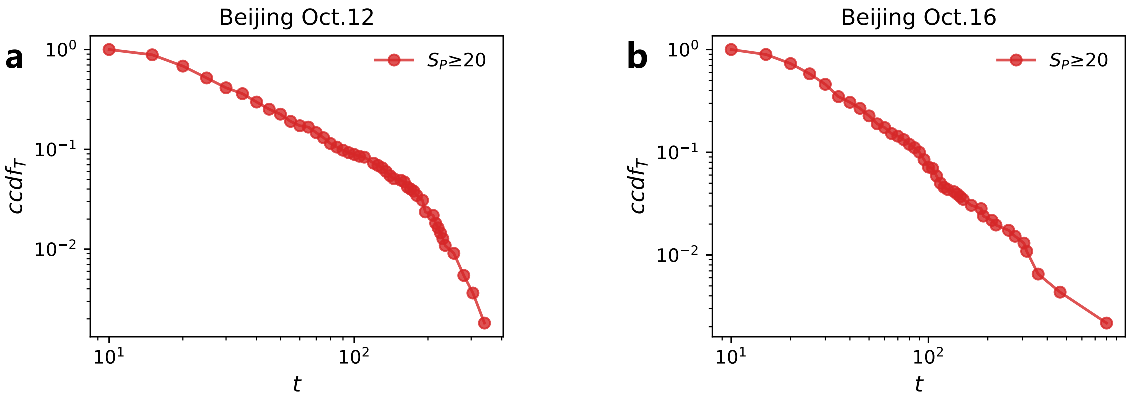

Fig. R21. Complementary cumulative distribution function of the jam duration T for large congestion components with maximal size $S_P \geq 20$ roads on (a) Oct. 12, Monday and (b) Oct. 16, Friday, 2015, in Beijing. The y-axis, i.e., CCDF of T, is the probability that jam duration T longer than or equal to time t.

R2C8.2. -Supp. fig 12 and 13 seem to show that larger congestions do not have very large time durations. Then what is the efficacy left of using a V15 predictor that detects congestion after 15 mins at which time most of the detected large congestions are close to finishing the growth stage. The analysis could include the actual growth trends against prediction times.

Response: We thank the reviewer very much for this important comment. We have analyzed the actual growth trends of the heavy bottlenecks against the prediction time, and discussed the efficacy of V_{15} in early identifying the potential large congestion.

Firstly, the V_{15} predictor is practically applicable for most of large congestions in the studied cities. As $ccdf_T$ shown in Fig. **R22**, it is suggested that 92% of heavy bottlenecks ($S_P \geqslant 25$) experienced total jam duration $T \geq 15$ minutes. For such heavy bottlenecks, initial growth speed within 15 minutes could be applied to design response strategies that prevent them from developing into the maximal size or reaching the full jam duration.

Secondly, V_{15} can be applied in identifying the most dangerous heavy jams, before they completely form gridlock area. $ccdf_{T_G}$ in Fig. **R22** indicates that 51% of heavy bottlenecks ($S_P \geqslant 25$), experienced growth duration $T_G \geq 15$ minutes. For these large congestions, V_{15} performs excellently (AUC around 0.96) in identifying them as heavy bottlenecks. Thus, it can help to design coordinated signal control strategy.

Thirdly, we can balance the prediction time and accuracy in practice. For bottlenecks with relatively short growth duration, their growth speed V_5 at 5 minutes (AUC around 0.85) or V_{10} at 10 minutes (AUC around 0.91) are also alternative good predictors. When pursuing early warning instead of accuracy, for example, one could use V_5. Furthermore, the predictors

V_5, V_{10}, and V_{15} could be comprehensively applied to early identify the heavy bottlenecks before maximal size or full jam duration.

In future, we would also combine more advanced machine learning technologies with our current framework to improve the comprehensive prediction efficiency. We have added the above new analysis in Supplementary Note 12 and Supplementary Fig. 39 of the revised SI, and discussed them in Subsection 2.5 of the revised Main text.

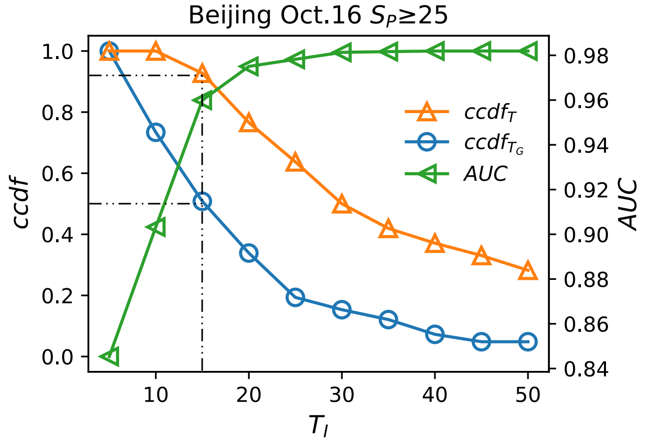

Fig. R22. CCDFs of growth duration T_G and total jam duration *T*, and AUC against the prediction time T_I for the major traffic congestions with size S_P larger than or equal to 25. The *x*-axis includes the prediction time of the initial 5 to 50 minutes of bottleneck growth. The left *y*-axis includes the CCDFs of growth duration T_G and total jam duration T. The right *y*-axis is the AUC of the prediction performance at the given prediction time T_I. The figures are plotted based on the bottleneck dynamics on Oct. 16, 2015, in Beijing.

Comments R2C9: Why are J_c values higher than J_b, are they calculated for all congestions or for those where the J_b is high? Can the authors clarify the fact that even when bottleneck or J_b is less similar still the associated congestions show more similarity?

Response: We thank the reviewer very much for this valuable comment. The values of J_C higher than J_B, could be because the congestion sources are stochastic, while the congestion propagation mechanism is fundamental. We clarify that we calculated overlap J_C for congestions associated with all the bottlenecks rather than those bottlenecks with higher overlap J_B in the manuscript. The values of J_C have been found higher than J_B, even when the bottlenecks are less similar (Fig. **R23**). For example, for congestion components whose bottlenecks are less similar (e.g., $S_L = 10$, J_B < 0.1), similarity J_C of the associated congestions is still slightly larger than J_B of the bottlenecks. This may be because the spatial and temporal congestion propagation have a fundamental pattern (Zhao et al., 2016). Thus, the overall congestion regions could include similar propagation pathways due to the congestion propagation mechanism. We have added the new analysis in Supplementary Note 9 and Supplementary Fig. 32 of the revised SI and discussed the explanation in Subsection 2.5 of the revised Main Text.

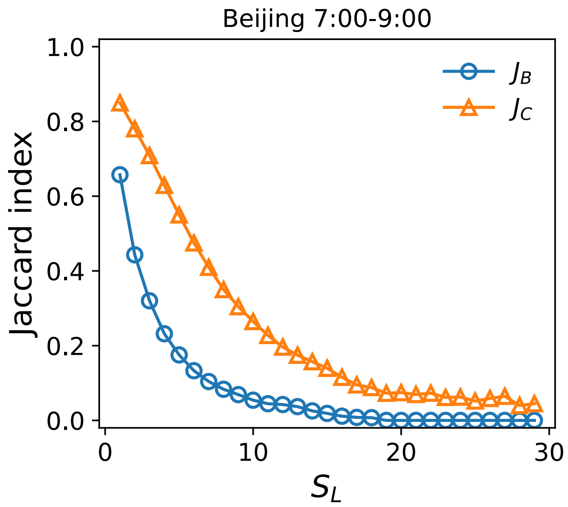

Fig. R23. Jaccard index J_B and Jaccard index J_C between two Fridays in different weeks, i.e., Oct. 9, and Oct. 16, 2015, in Beijing, for bottlenecks with different size threshold S_L (i.e., $S_P \geq S_L$). J_B is the ratio between the size of overlapped bottlenecks and the size of the union of bottlenecks in two different days. J_C is the ratio between the size of the overlap of the associated congestions and the size of the union of the associated congestions in two different days.

References:

Zhao, J., Li, D., Sanhedrai, H., Cohen, R., Havlin, S. 2016. Spatio-temporal propagation of cascading overload failures in spatially embedded networks. Nature Communications 7 (1), 10094.

Comments R2C10: Using the term 'free-flow traffic state' for non-congested links is not generally common usage in traffic science. Since the congestion weight < 0.5 (eq. 9) won't represent free flow conditions. I suggest using 'uncongested-state' vs 'congested-state'. An uncongested state might still have travel times somewhat higher than free flow.

Response: We deeply appreciate the reviewer's kind suggestion. We have thoroughly checked the manuscript and revised the expressions of such "free flow conditions" to "uncongested-state" in the revised manuscript.

Comments R2C11: Fig. 5, it would also be interesting to show false positives or the false alarms that the predictor generates.

Response: We thank the reviewer very much for this good suggestion. We have plotted the false alarm bottlenecks in Fig. **R24**, and found the false alarm bottlenecks are also located in significantly different road segments in rush and non-rush hours. We have added these figures of the false alarms (the false positive bottlenecks) in Supplementary Fig. 34 of the revised SI and discussed them in Subsection 2.5 of the revised Main Text.

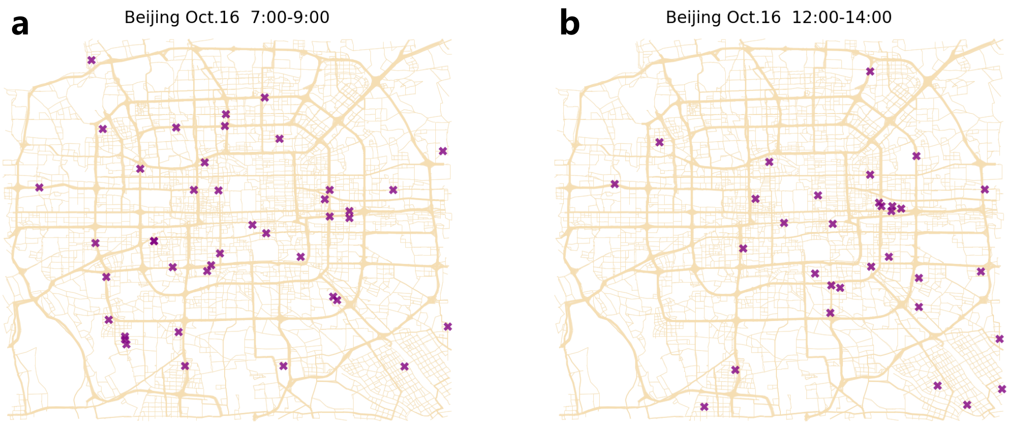

Fig. R24. The false positive (purple stars) bottlenecks during (a) rush hours and (b) non-rush hours on Oct. 16, 2015, in Beijing. Here, the predictor is the initial growth speed at the first 15 minutes of the growth duration**.** The false positive bottlenecks are the minor bottlenecks with maximal size S_P smaller than 20 roads yet wrongly predicted as major bottlenecks in the 15 minutes of their growth stage, given the false positive rate of 5%.

Reviewer #3 (Remarks to the Author):

The manuscript is built upon a method of identifying traffic bottleneck links and observing the propagation and dissipation of traffic jams corresponding to these bottlenecks. The results show that recovery and growth patterns of traffic jams are distinctively different, using the evidence from real traffic data collected from two city networks. The main finding is that the growth speed of congestion measured in 5-15 mins of the emergence is significantly correlated with the maximal size of the growing congestion pocket, and thus can be used to make accurate predictions of major traffic jams. The idea of the paper is original and the results are immensely useful and both missing in the existing literature, and therefore, I recommend publication of the manuscript after necessary revisions rendered by the comments below.

Response: We thank the reviewer very much for the insightful comments, and for considering "the idea of the paper is original and the results are immensely useful". We have carefully considered all the comments of the reviewer and solved all the issues that the reviewer raised. Below we provide the point-by-point responses.

Comments R3C1: A general comment on the main text is that in many parts it deviates from the main message. As an example, the material written around equations 2 and 3 did not belong to the main text in my opinion. The manuscript can become much sharper and better deliver its main finding by highlighting the main theme, which is as suggested by the title, is the existence of early signal of heavy congestion in real traffic network data.

Response: We thank the reviewer very much for this important suggestion. We have reorganized the manuscript, and removed the contents that deviated from the main text, or moved them to SI. First, we have simplified the material around Eqs. (2) and (3). Second, as the reviewer suggested, we have removed the content lines 184-196 that explained the observed difference between growth and recovery durations. Third, we have removed the two places that resembled the spread of traffic congestion to that of the virus.

Comments R3C2: I found the explanations supporting the observed difference between growth and recovery speeds troubling. The scaling laws derived from the data seem valid and very useful, however, the quite trivial explanation should be the fact that vehicular flows arrive at the congested component with a much higher speed than they leave the jam. The analysis given in lines 184-196 lacks rigor. Basically, the mathematics presented, only suggest that the slowest congestion can grow is at the rate of 0 but there is no upper bound. I strongly suggest that authors remove that paragraph and explain the phenomenon through domain knowledge of traffic network flows.

Response: We thank the reviewer very much for this important suggestion. We have removed lines 184-196 and improved our explanation, based on the theory of phase transition in traffic network flows. The propagation of urban traffic congestion has been regarded as the percolation in the system (Zeng et al., 2019; Zeng et al., 2020), and the traffic systems have

exhibited phase transitions as the traffic congestion components are close to the critical point in the self-organized process (Nagel and Schreckenberg, 1992; Zhang et al., 2019). It has been found that the recovery of the systems against the perturbations can be slowing down, when the system is close to such critical point, also called “critical slowing down” (Dakos et al., 2008; Scheffer et al., 2009; Scheffer, 2010; Veraart et al., 2012). As the results in our manuscript, the power-law distribution of the congestion dissipation indicates that the traffic systems might be near the critical points of the self-organized process. Thus, the slower recovery of the system could be the result of the “critical slowing down” mechanisms. We have added this important explanation in Subsection 2.2 of the revised Main Text.

References:

Dakos, V., Scheffer, M., Van Nes, E.H., Brovkin, V., Petoukhov, V., Held, H. 2008. Slowing down as an early warning signal for abrupt climate change. Proceedings of the National Academy of Sciences 105 (38), 14308-14312.

Nagel, K., Schreckenberg, M. 1992. A cellular automaton model for freeway traffic. Journal de physique I 2 (12), 2221-2229.

Scheffer, M. 2010. Foreseeing tipping points. Nature 467 (7314), 411-412.

Scheffer, M., Bascompte, J., Brock, W.A., Brovkin, V., Carpenter, S.R., Dakos, V., Held, H., Van Nes, E.H., Rietkerk, M., Sugihara, G. 2009. Early-warning signals for critical transitions. Nature 461 (7260), 53-59.

Veraart, A.J., Faassen, E.J., Dakos, V., van Nes, E.H., Lürling, M., Scheffer, M. 2012. Recovery rates reflect distance to a tipping point in a living system. Nature 481 (7381), 357-359.

Zeng, G., Gao, J., Shekhtman, L., Guo, S., Lv, W., Wu, J., Liu, H., Levy, O., Li, D., Gao, Z. 2020. Multiple metastable network states in urban traffic. Proceedings of the National Academy of Sciences of the United States of America 117 (30), 17528-17534.

Zeng, G., Li, D., Guo, S., Gao, L., Gao, Z., Stanley, H.E., Havlin, S. 2019. Switch between critical percolation modes in city traffic dynamics. Proceedings of the National Academy of Sciences of the United States of America 116 (1), 23-28.

Zhang, L., Zeng, G., Li, D., Huang, H.-J., Stanley, H.E., Havlin, S. 2019. Scale-free resilience of real traffic jams. Proceedings of the National Academy of Sciences of the United States of America 116 (18), 8673-8678.

Comments R3C3: In Fig. 2 for both cities <r> is inversely changing relative to \beta_R, and perhaps surprisingly, the relationship is not as clear with \beta_G. Is the average recovery to growth rate is heavily influenced by the heavy tail in recovery duration distribution? It would be interesting to investigate this, and possibly find an explanation for the phenomenon.

Response: We thank the reviewer very much for this important and inspiring suggestion. We have performed additional analysis in the revised manuscript and found that the larger average ratio $<r>$ between recovery duration T_R and growth duration T_G is indeed influenced by the heavy tail in recovery duration distribution.

It is shown in Figs. **R25**a and b that, the upper bounds of the ratio r for bottlenecks with $T_R \geq 30$ are much larger than that for $T_R < 30$. In Figs. **R25**c and d, it is shown that the average ratio $<r>$ for bottlenecks with $T_R \geq 30$ are stable in different workdays and larger than the values for bottlenecks with $T_R < 30$. This indicates that the larger values of $<r>$ are influenced by the heavy tails of the recovery duration T_R. The “critical slowing down” effect (Dakos et al., 2008) becomes more significant for those bottlenecks with large T_R. This could be due to the fact that nonlinear mechanisms exist between the congestion propagation and

dissipation processes given the complex urban traffic network topologies and the travelers' real-time self-adaptive route choice behaviors (Dakos et al., 2008; Lee et al., 2017; Akbarzadeh and Estrada, 2018). We have added these new results in Supplementary Note 6 and Supplementary Fig. 17 of the revised SI, and discussed them in Subsection 2.2 of the revised Main Text.

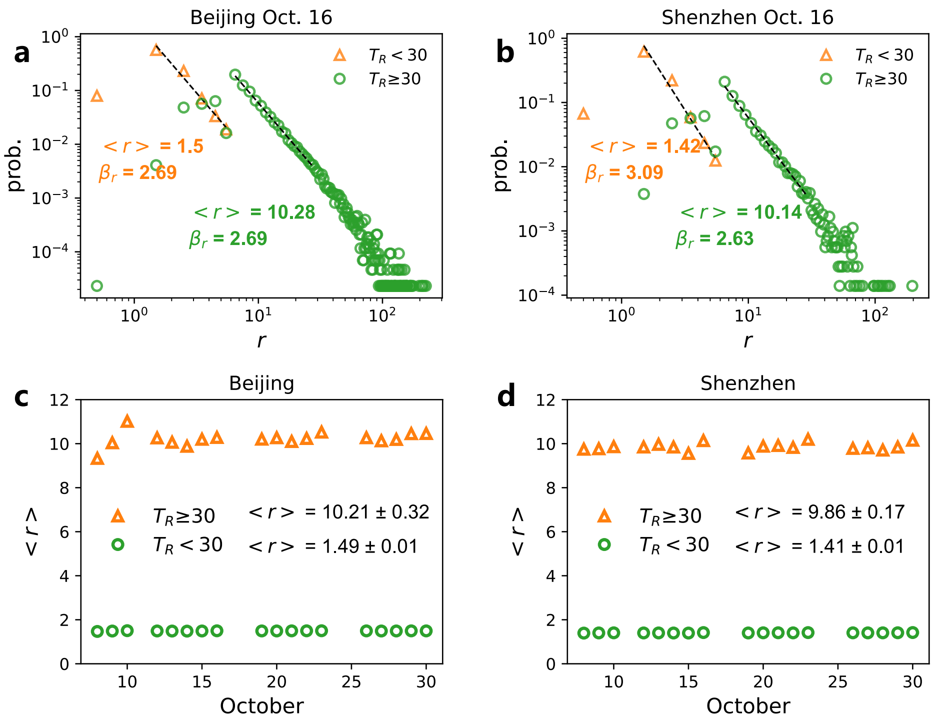

Fig. R25. Values of mean ratio <*r*> between recovery duration T_R and growth duration T_G for bottlenecks with different T_R. a and **b.** Distributions of ratio *r* between recovery duration and growth duration respectively for bottlenecks with recovery duration $T_R \geq 30$ minutes and $T_R < 30$ minutes. **c** and **d.** Mean value <*r*> for two groups of bottlenecks on 18 workdays in Oct. 2015, of (c) Beijing and (d) Shenzhen.

References:

Akbarzadeh, M., Estrada, E. 2018. Communicability geometry captures traffic flows in cities. Nature Human Behaviour 2 (9), 645-652.

Dakos, V., Scheffer, M., Van Nes, E.H., Brovkin, V., Petoukhov, V., Held, H. 2008. Slowing down as an early warning signal for abrupt climate change. Proceedings of the National Academy of Sciences 105 (38), 14308-14312.

Lee, M., Barbosa, H., Youn, H., Holme, P., Ghoshal, G. 2017. Morphology of travel routes and the organization of cities. Nature Communications 8 (1), 2229.

In the following I present a separate list of comments which I consider to be of minor importance compared to the above.

Comments R3C4: In two places, it is suggested that the growth/recovery discrepancy in traffic resembles natural phenomena such as spread of a virus. Unfortunately, this is due to very different but quite apparent reasons. As suggested in another comment, the high speed of vehicles when arriving to the jam and their departure starting at very low speed, explains this

phenomenon in traffic networks. I feel the statements can be misleading to some portions of the audience for this paper and recommend that it would be removed or majorly revised.

Response: We deeply appreciate the reviewer's important suggestion. We agree with the reviewer, and have removed the sentences that resemble traffic propagation and the spread of a virus in the revised Main Text.

Comments R3C5: DTB is introduced as the acronym for "dynamics of traffic bottlenecks", but then used all over the text, seemingly, to refer to "bottlenecks". After authors introduce the method used to find the trunks, simply using "bottleneck (link)" is superior to the use of "DTB" to refer to those links.

Response: We thank the reviewer very much for the careful examination and this important suggestion. We have thoroughly checked the manuscript and revised the "DTB" to "bottleneck" in the revised manuscript after introducing the method to find the trunks.

Comments R3C6:

R3C6.1. When observing the recovery of a congested component, does the algorithm: i) count the (decreasing number of) connected road segments that remain congested over time? Or ii) follows the set of congested roads recorded at the peak of the component size?

Response: We thank the reviewer very much for this important question. We have added a demo to clarify that our algorithm counts the road segments that remained congested over time, when observing the recovery of a congestion component. As the recovery stage shown in Fig. **R26**b, our size $S(t)$, i.e., the number of existing congested roads, includes newly developed congested roads and removes those recovered. Thus, the existing size S is decreasing over time in the recovery process due to more new recoveries than new congestions. We have added these clarifications in Supplementary Note 3 and Supplementary Fig. 7 of the revised SI, and discussed them in Methods of the revised Main Text.

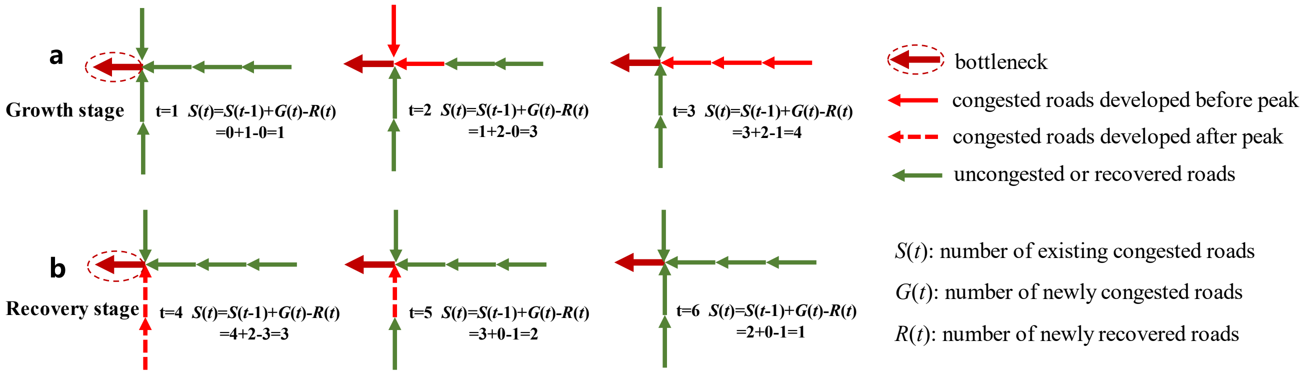

Fig. R26. Demonstration of congestion size associated with a bottleneck in (a) the growth stage and **(b)** the recovery stage.

R3C6.2. Also, the trunk may recover sooner than the whole congested component, and after its disappearance other neighboring roads may become congested. Will they be considered as

branches of the same tree even though they appeared after the bottleneck's recovery? Please clarify this in the Methods section or the main text.

Response: We thank the reviewer very much for this comment. We have clarified that in our algorithm, if the recovery of the bottleneck is sooner than other neighboring branches, the branches that belonged to the recovered trunk are not considered anymore to be associated with the original (recovered) bottleneck. These branches are considered as a new tree that could be the child of the previous tree, and the earliest congested road segment in the new child tree is regarded as the trunk of the new tree. We have added these clarifications in the Methods of the revised Main Text.

Comments R3C7: In Fig. 2 for each congested component, propagation and dissipation durations are plotted in a & b panels. Why are there so many more data points associated with T_R, although they should be equal. Is there a missing explanation or plotting error there?

Response: We deeply appreciate the reviewer's careful examination of the figures and this question. It is not a plotting error but due to that the probability of growth duration T_G is $p(T_G) = 0$ in Figs. 2a and b in the range above 100 minutes, resulting in the empty data point in the larger range. Meanwhile, the data points of T_G and T_R have the same data intervals below around 100 minutes. The slope of the distribution for T_G is larger than that for T_R, so it seems to have less data points.

Comments R3C8: The Jaccard index of 0.05 indicates that 5% of all bottlenecks appearing in two days are repeated in both days. This has a subtle difference to what is stated in the manuscript.

Response: We thank the reviewer very much for bringing this important source of confusion to our attention. We revised the expressions in Subsection 2.5 of the revised Main Text as the reviewer commented that the overlap of bottlenecks is actually the ratio between the repeated bottlenecks and the union of all the bottlenecks in *both* days.

Comments R3C9: A concise explanation of the bottleneck identification method (presented in earlier works) in the main text can aid the reader's understanding and will improve the manuscript.

Response: We deeply appreciate the reviewer's suggestion. We have added an explanation of the bottleneck identification method in the revised main text. The bottleneck is the downstream road that has been congested for the longest duration, and the new congestions associated with it are the neighbouring upstream roads that became congested no longer than a pre-defined time θ after the bottleneck or an existing downstream neighboring congested road. We have added this concise explanation of the bottleneck identification method in Subsection 2.1 of the revised Main Text.

REVIEWER COMMENTS

Reviewer #1 (Remarks to the Author):

I want to start by thanking the authors for all their additional work and for the revisions to the manuscript. I believe that the study is now greatly stronger and more replicable than before.

I nonetheless still have a number of concerns that I strongly believe need to be addressed. The reason is that some incorrect claims reported in first publications on a topic tend to be viewed by the community as a certainty, which then requires a lot of effort to correct.

Specifically, I have complete opposition to notion that this study -- based on the data presented so far -- would claim that the distribution of the growth durations T_G decays as a power law.

I will come to it later(point 3), though.

1. I believe that Fig. 1 would benefit from have diagrams such as those shown in Figs. R1, R5, and R16 prior to the current panels so that the reader can better understand what is being done and what choices were made.

2. I believe that any impartial observer would agree that the presentation of distribution plots using the survival functon(or complementary cumulative distribution) is much cleaner and reliable than the probability density -- compare Fig. R13 to Fig. 2a, b. I recommend that all plots show the survival function, including in the SI.

Also, when using the survival function there is NO NEED to bin the data!

3. The disagreement I have with the authors relates to the claim of the behavior of p(T_G). Three facts concern me: first, the data is clearly curved throughout; second, the claimed power law regime extends over considerable less than one order of magnitude on the variable T_g; third, the calculated power-law "exponent" is larger than 5. Each of those facts alone would suggest caution in claiming power law behavior; all together, they make the claim unsustainable.

I think it is critical than the author show log-linear plots of the data for comparison.

For example, SFigs. 19 and 20 (R14, R15) should show in both cases the curves for the fits to exponential and power law, should use the survival function, and should not bin the data.

4. Tables throughout the rebuttal are really hard to read. My greatest concern is about claims relating to T_G. However, the results are presented in 3 tables, each one showing some partial result for each of the variables.

Another problem is the choice of x_min = 15 for T_G. When x_min = 10, one cannot reject the hypothesis that the data could have been drawn from an exponential distribution but rejects (quite categorically) the power law hypothesis).

Moreover, when going from x_min = 10 to x_min = 15, one loses the ability to explain about half the data.

So, I must ask, why are the authors choosing to set x_min = 15?

5. I really do not find the answer to my comment R2C3 convincing. There is really no difference between estimates for beta_R during the weekends and weekdays.

Reviewer #2 (Remarks to the Author):

The authors have fully addressed each of my original comments sufficiently. I have no further comments on the manuscript.

Reviewer #3 (Remarks to the Author):

Authors have provided appropriate response and actions to my few major comments. I believe that these changes though small have solved important issues and substantially improved the manuscript. In

particular, authors have done a good job with the removal of loose interpretations, tightening the logic behind their conclusions, and diving deeper in the analysis of the physics of congestion.

The propagation and dissipation of traffic has been long studied but merely in road/highway level. Studies such as this submission are much needed to deepen our understanding of network level congestion dynamcis. Overall, I recomemnd publication of this submission but encourage the authors to consider my remaining comments below.

- I had a comment suggesting elmination of DTB (since it is not adding anything to the concept of bottleneck) and using "bottlenecks" instead which is easy to understand for any reader. Authors seemed to agree with me in their response but there are many usages of DTB still in the revised manuscript.

- The analysis of Supp Fig. 17 is very interesting to me, and might be for other readers as well, so it may deserve a bit more coverage. I suggest authors add one or two sentences to their expalanation in the main text.

- In the new version of the manuscript, a big paragraph is dedicated to review of the literature on congestion mechanisms from Transport Engineering and Classical Physics. I, however, did not find it an improvement. The reviewed literature are deeply focused on the mechanisms occuring inside a road space (a road segment or highway) and they do not explain the phenomena in a network or roads--thus irrelevant to the contributions of this paper.

Responds to the reviewers' comments of

"Spatiotemporal dynamics of traffic bottlenecks: an early signal of heavy congestions"

by Jinxiao Duan, Guanwen Zeng, Nimrod Serok, Daqing Li, Efrat Blumenfeld Lieberthal*, Hai-Jun Huang* and Shlomo Havlin*

(Manuscript No.: NCOMMS-23-10587A)

We would like to first thank the anonymous reviewers for their valuable comments and suggestions. Listed below is the itemized response to all review comments.

Reviewer #1 (Remarks to the Author):

I want to start by thanking the authors for all their additional work and for the revisions to the manuscript. I believe that the study is now greatly stronger and more replicable than before.

Response: We thank the reviewer very much for the helpful comments, and for considering that our additional work and revisions make the study "greatly stronger and more replicable". We have carefully considered all the new comments, and revised the manuscript as suggested. Below we provide the point-by-point responses.

I nonetheless still have a number of concerns that I strongly believe need to be addressed. The reason is that some incorrect claims reported in first publications on a topic tend to be viewed by the community as a certainty, which then requires a lot of effort to correct.

Specifically, I have complete opposition to notion that this study -- based on the data presented so far -- would claim that the distribution of the growth durations T_G decays as a power law.

I will come to it later (point 3), though.

Response: We thank the reviewer very much for these kind comments. We have carefully considered these suggestions, especially the distribution for the growth duration. We agree with the reviewer that there might be better model to explain growth duration T_G. We have conducted significantly more analysis and found that the exponential distributions could better describe the growth duration. We will describe this point in detail in our response for point 3.

R1C1: I believe that Fig. 1 would benefit from have diagrams such as those shown in Figs. R1, R5, and R16 prior to the current panels so that the reader can better understand what is being done and what choices were made.

Response: We thank the reviewer very much for this kind comment. We have added diagrams in the front of the Fig. 1 for demonstration in order to help to understand our method. As shown in Fig. R1a, a traffic bottleneck, i.e., the earliest congested road in the downstream of the congestion component, emerged at time $t = 1$. The bottleneck develops and includes two more congested roads in the congested component at time $t = 2$. At time $t = 3$, another two new

congested roads developed in the bottom branch of the upstream roads, and 1 road recovered from the congestion component in the right branch of the upstream roads. These snapshots show the propagation dynamics of the bottleneck. We have added these diagrams in Fig. 1a of the revised Main Text, and discussed them in the revised manuscript.

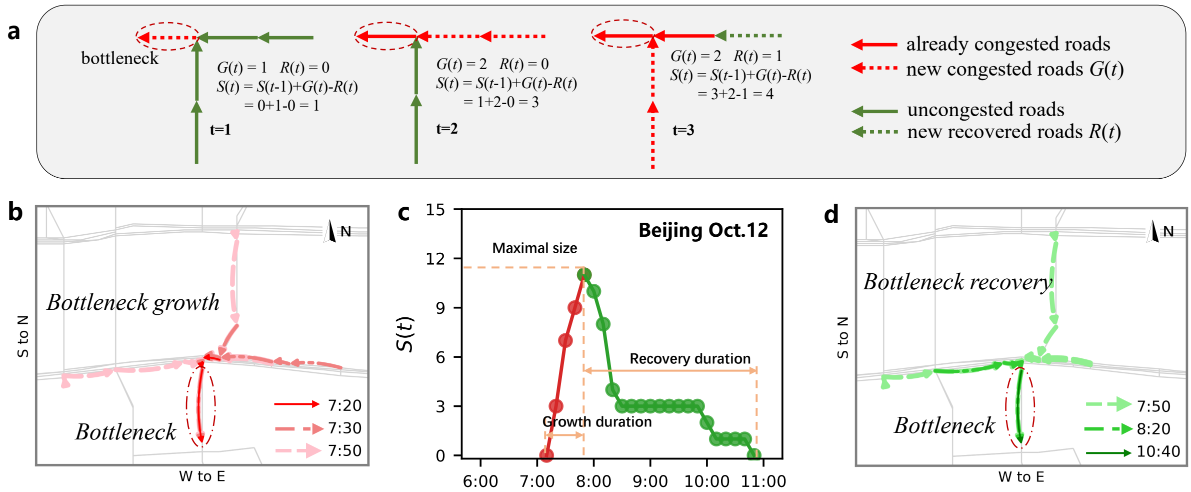

Fig. R1. Propagation and dissipation of a typical bottleneck in traffic network. a. Demonstration of congestion size *S* associated with a bottleneck in an illustrated network. **b.** Several snapshots of the congested road segments during growth stage at 7:20, 7:30, and 7:50 for a specific but typical bottleneck on Monday, October 12, 2015, in Beijing. The congestions originated from the bottleneck (circled red solid links), and gradually developed to more congestions (light red dashed links) in the upstream neighbouring streets. The arrows of the links are the directions of traffic flow. **c.** The size *S* changes over time. This congestion component lasted for more than 3 hours after its bottleneck emerged. Its maximal size S_P reached 11 road segments at 7:50, and then the size *S* began to decrease until it completely vanished at 10:50. Note that the recovery duration is much longer than the growth, which is a common feature of the bottleneck dynamics (see Fig. 2). **d.** Several snapshots of the congested road segments during the recovery stage at 7:50, 8:20, and 10:40. The congestion component gradually dissolves after its maximal size (light green dashed links) as the road segments become no longer congested.

R1C2: I believe that any impartial observer would agree that the presentation of distribution plots using the survival functon(or complementary cumulative distribution) is much cleaner and reliable than the probability density -- compare Fig. R13 to Fig. 2a, b. I recommend that all plots show the survival function, including in the SI.

Also, when using the survival function there is NO NEED to bin the data!

Response: We thank the reviewer very much for this comment. We have shown survival functions (or CCDF) of growth duration T_G, recovery duration T_R, and the ratio *r* between T_R and T_G for all the plots of the main text and SI. We have added the CCDFs of T_G and T_R in Figs. 2 a to d in the revised Main Text and Supplementary Figs. 8 and 9 in the revised SI. We have added the CCDF of ratio *r* in Supplementary Figs. 14 and 15 of the revised SI. We have added the CCDFs of T_G and T_R for different definitions of size $S(t)$ in Supplementary

[Figs. 20 and 21](#) of the revised SI. We also listed the figures in Figs. R**2** to R**9** in the following.

Following the reviewer, we did not bin the data. The time resolution of growth duration and recovery duration both have a time interval of 5 minutes.

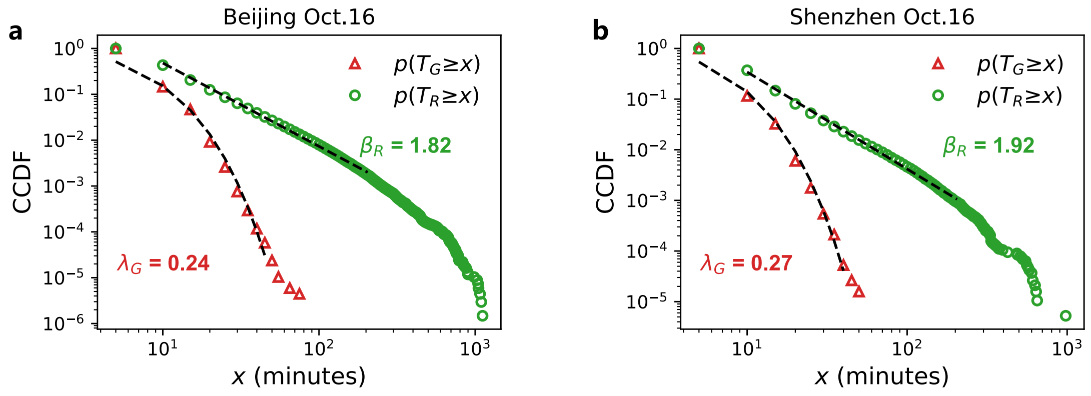

Fig. R2. The CCDFs of the growth duration T_G and recovery duration T_R in (a) Beijing and (b) Shenzhen, on Oct. 16, 2015. The CCDF of growth duration T_G is well approximated by an exponential distribution. The CCDF of recovery duration T_R is well approximated by a power law distribution.

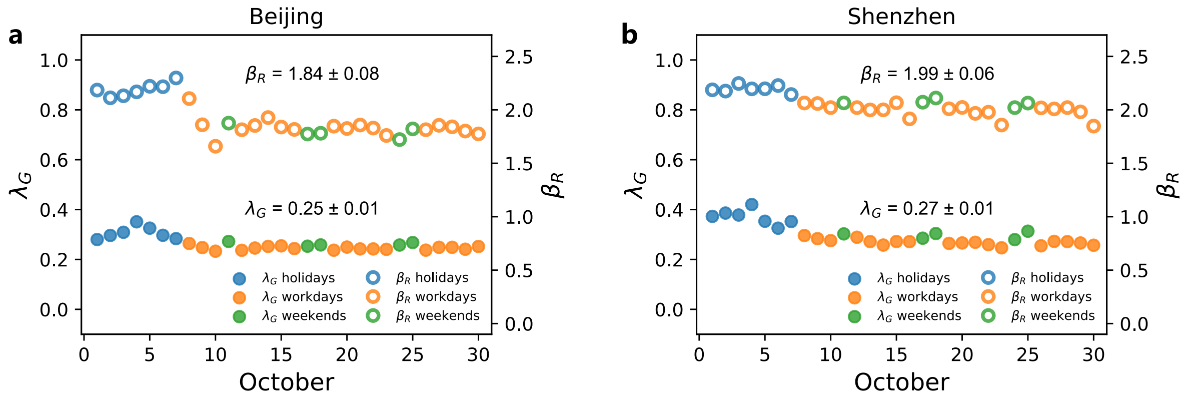

Fig. R3. Exponents of the CCDFs of the growth duration T_G and recovery duration T_R in the entire month of October, 2015. a and **b** show the exponents λ_G and β_R for each day in the entire month for (a) Beijing and (b) Shenzhen. The days between October 1 and October 7 are the seven holidays of China's National Day. October 11, 17, 18, 24, and 25 are the regular weekends, and the other 18 days are workdays. The mean values and standard errors are calculated for 18 workdays.

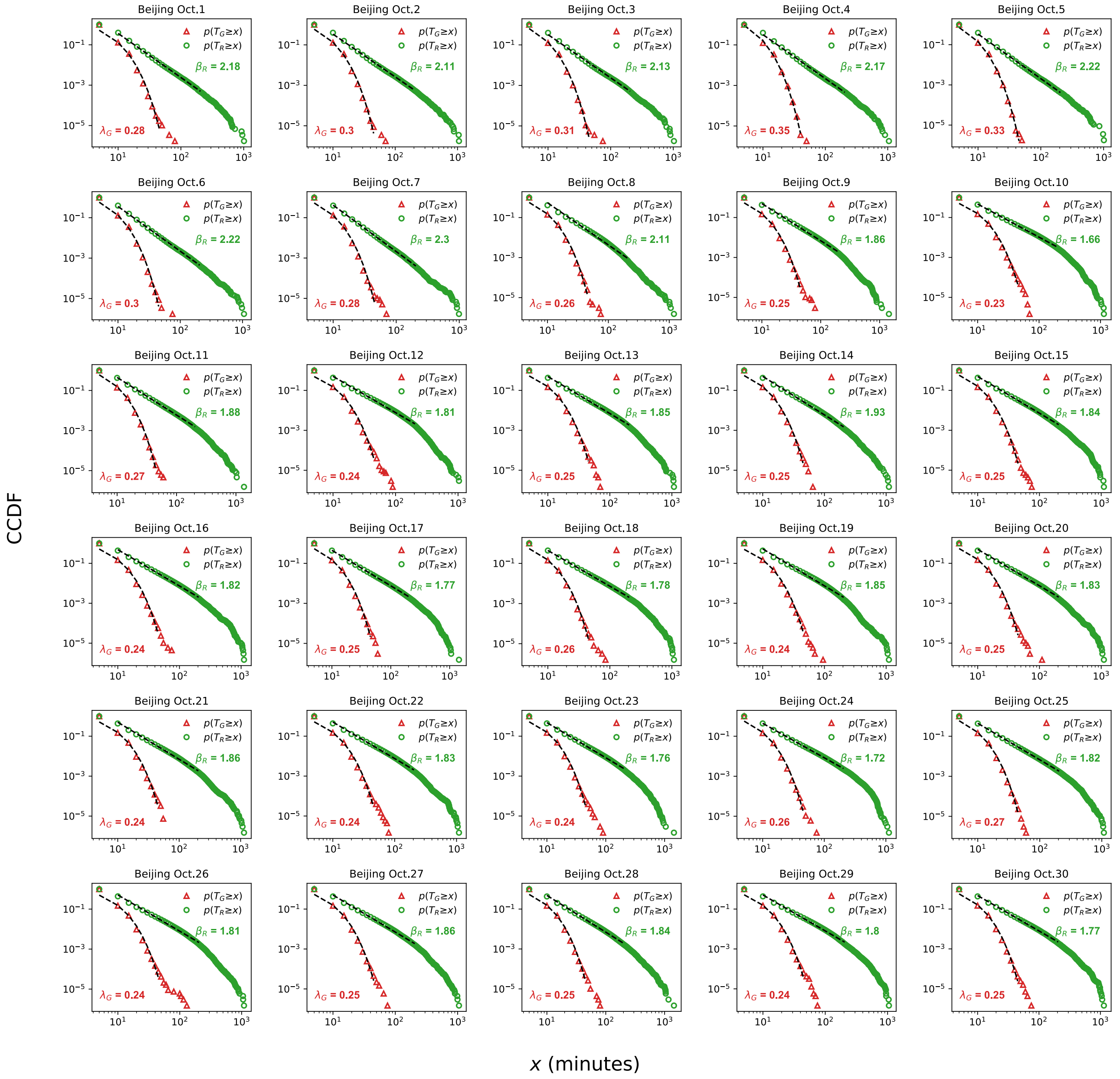

Fig. R4. The CCDFs of the growth duration T_G and the recovery duration T_R on 30 days of October 2015 in Beijing. The CCDF of growth duration T_G is well approximated by an exponential distribution. The CCDF of recovery duration T_R is well approximated by a power law distribution. The distributions and exponents are similar for the same type of days. October 1 to October 7 are seven holidays of China's National Day, Oct. 11, 17, 18, 24, and 25 are the regular weekends, and the other 18 days are the workdays.

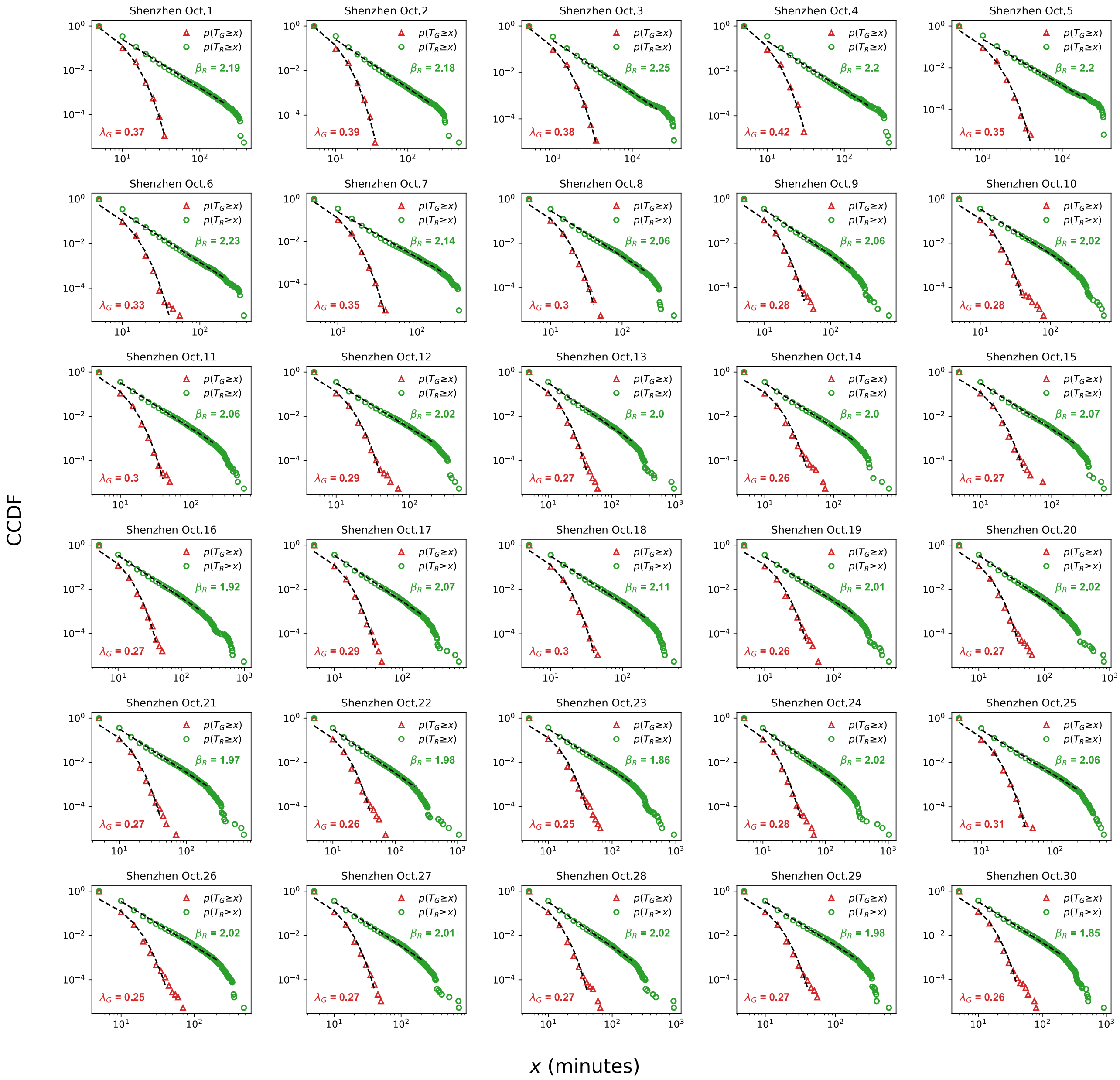

Fig. R5. The CCDFs of the growth duration T_G and the recovery duration T_R on 30 days of October 2015 in Shenzhen. The CCDF of growth duration T_G is well approximated by an exponential distribution. The CCDF of recovery duration T_R is well approximated by a power law distribution. The distributions and exponents are similar for the same type of days. October 1 to October 7 are seven holidays of China's National Day, Oct. 11, 17, 18, 24, and 25 are the regular weekends, and the other 18 days are the workdays.

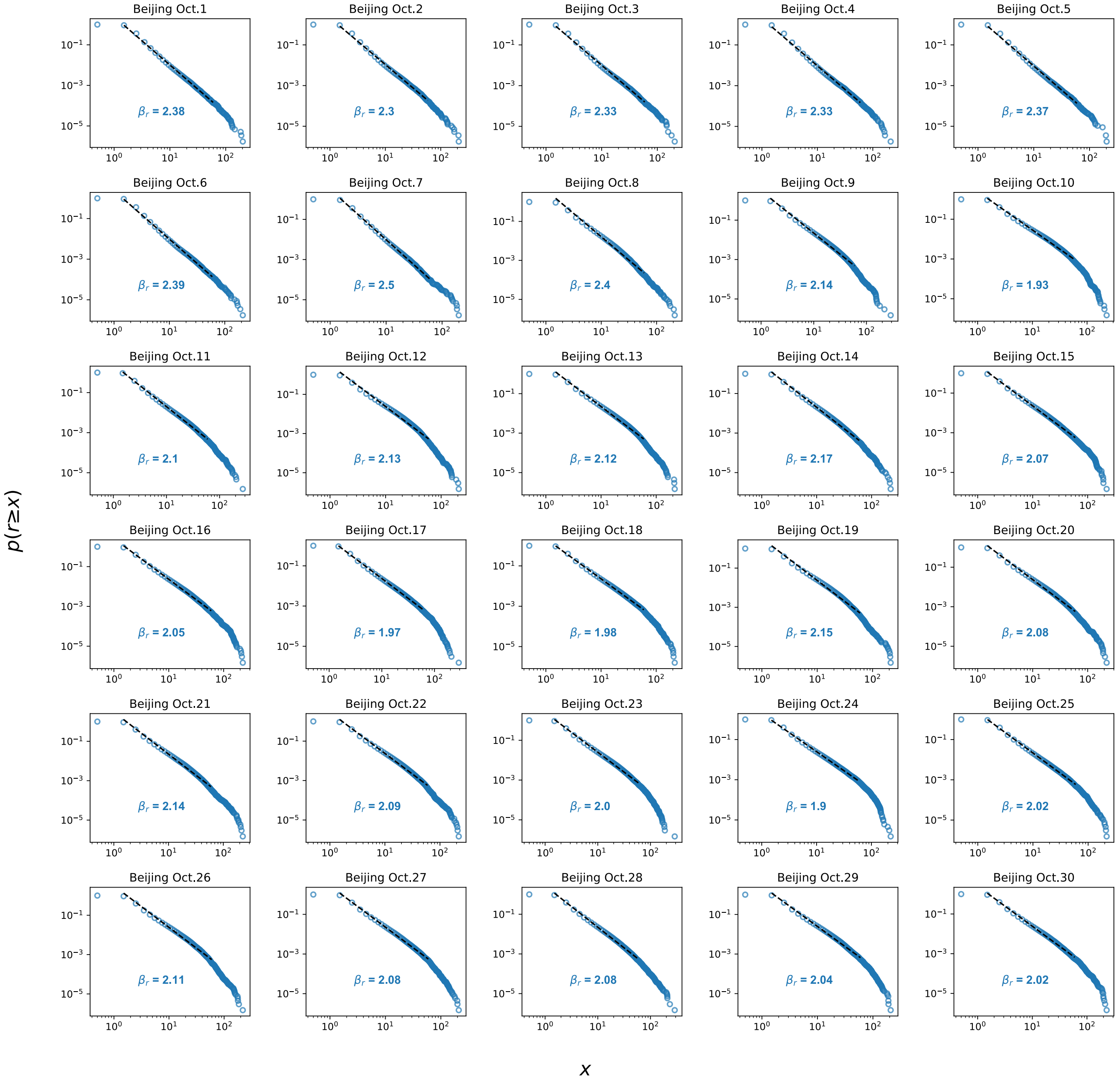

Fig. R6. The CCDF of ratio *r* between recovery duration T_R and growth duration T_G on 30 days of October 2015 in Beijing. Ratio r between recovery duration and growth duration approximately follows similar power-law patterns $p(r \geq x) \sim x^{-\beta_r}$ in different days.

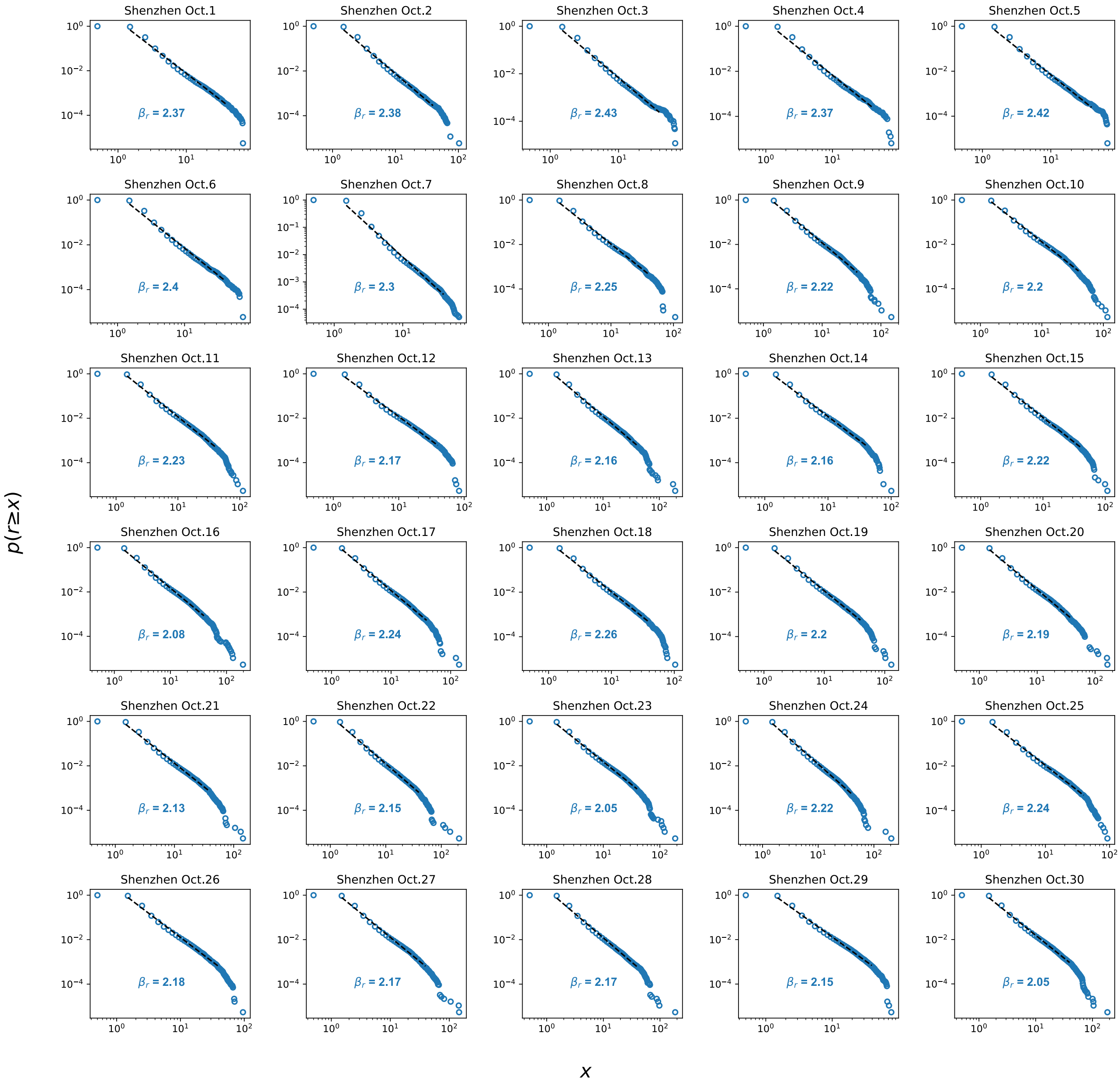

Fig. R7. The CCDF of ratio *r* between recovery duration T_R and growth duration T_G on 30 days of October 2015 in Shenzhen. Ratio *r* between recovery duration and growth duration approximately follows similar power-law patterns $p(r \geq x) \sim x^{-\beta_r}$ in different days.

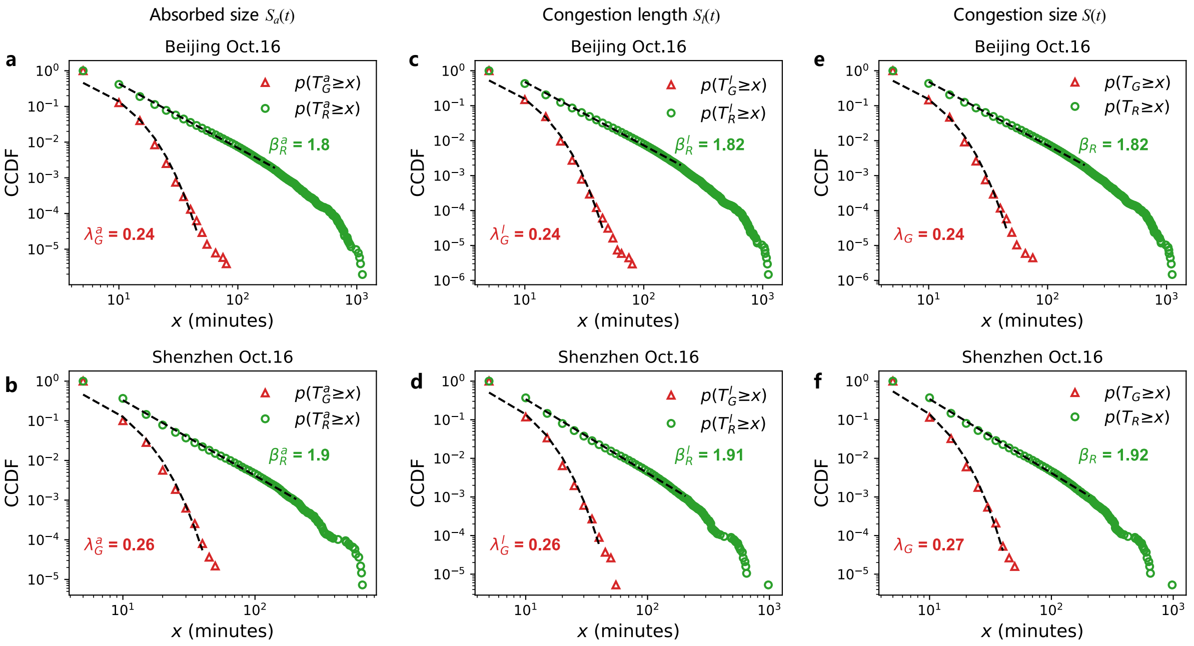

Fig. R8. The CCDFs of growth duration (red triangles) and recovery duration (green circles) obtained by three definitions of congestion size. a and **b**. The CCDFs of the growth duration T_G^a and the recovery duration T_R^a identified based on the absorbed size $S_a(t)$ on October 16, Friday, 2015, for (a) Beijing and (b) Shenzhen. **c** and **d**. The CCDFs of the growth duration T_G^l and the recovery duration T_R^l identified based on the congestion length $S_l(t)$ on October 16, Friday, 2015, for (c) Beijing and (d) Shenzhen. **e** and **f**. The CCDFs of the growth duration T_G and the recovery duration T_R identified based on the size $S(t)$ defined in the main text, on October 16, Friday, 2015, for (e) Beijing and (f) Shenzhen.

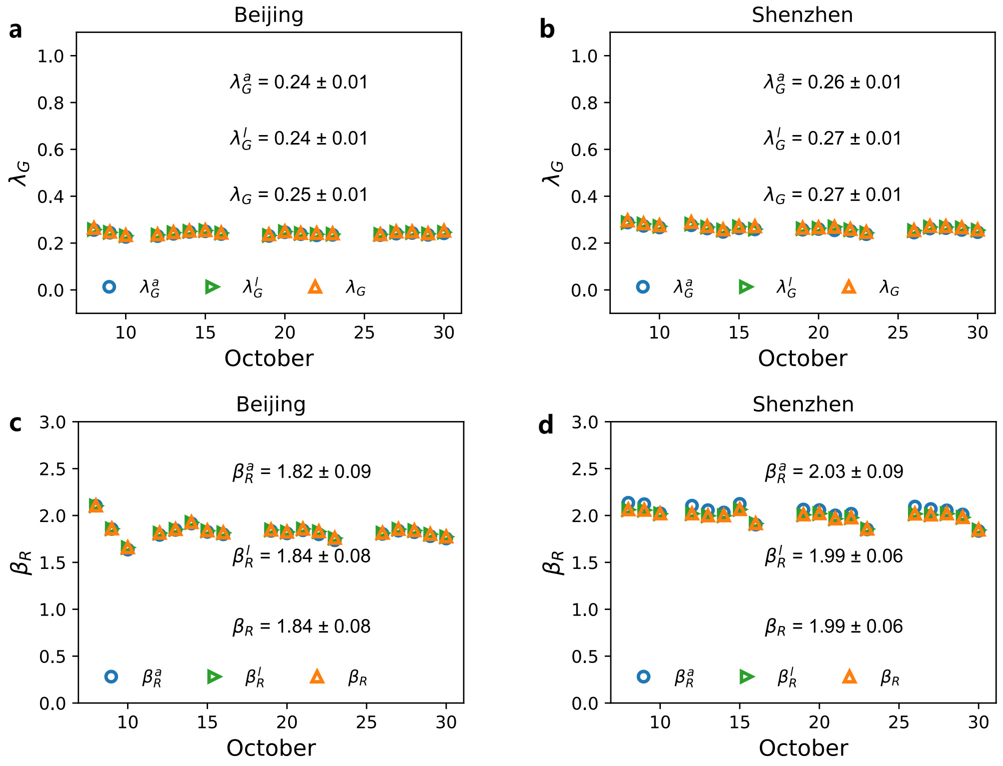

Fig. R9. Exponents for CCDFs of the growth duration and the recovery duration obtained by three definitions of congestion size, on 18 workdays in October, 2015. a and **b.** Exponents λ_G^a, λ_G^l, and λ_G

for the exponential distributions of CCDF of the growth duration respectively obtained by the dynamics of absorbed size $S_a(t)$, congestion length $S_l(t)$ and size $S(t)$, for 18 workdays in (a) Beijing and (b) Shenzhen. **c** and **d.** Exponents β_R^a, β_R^l, and β_R for the power-law distributions of CCDF of the recovery duration respectively obtained by the dynamics of absorbed size $S_a(t)$, congestion length $S_l(t)$ and size $S(t)$, for 18 workdays in (c) Beijing and (d) Shenzhen.

R1C3: The disagreement I have with the authors relates to the claim of the behavior of p(T_G). Three facts concern me: first, the data is clearly curved throughout; second, the claimed power law regime extends over considerable less than one order of magnitude on the variable T_g; third, the calculated power-law "exponent" is larger than 5. Each of those facts alone would suggest caution in claiming power law behavior; all together, they make the claim unsustainable.

I think it is critical than the author show log-linear plots of the data for comparison.

For example, SFigs. 19 and 20 (R14, R15) should show in both cases the curves for the fits to exponential and power law, should use the survival function, and should not bin the data.

Response: We thank the reviewer very much for this comment. We have carefully considered the reviewer's suggestion and fully agree with it after performing extensive new tests. We have shown the survival functions of growth duration T_G, and they could be indeed better approximated by exponential distributions. As seen in Figs. R**10**, the main parts (over 99% of the data) of the survival function of growth duration are well fitted by exponential distribution $p(T_G \geq x) \sim e^{-\lambda_G x}$. The tail of the CCDF of T_G seems to be a little fatter than the exponential distribution, yet they account for very small fraction of the data. Furthermore, the results on many days in the two cities (Figs. R**11** and R**12**) show that the growth duration on different days could be fitted by similar exponential distributions. We have added these results in Supplementary Figs. 10 and 11 of the revised SI.

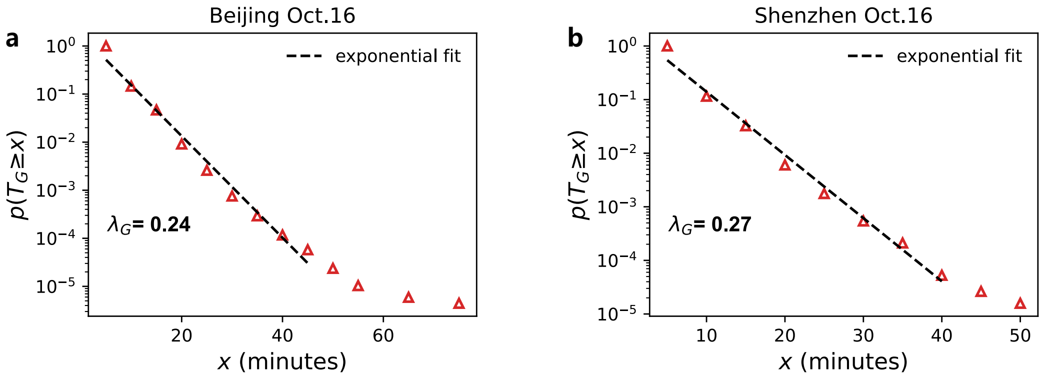

Fig. R10. The CCDF of growth duration T_G in (a) Beijing and (b) Shenzhen, in log-linear plots, on Oct. 16, 2015. The main part of the data is well approximated by exponential distributions. The data is not binned.

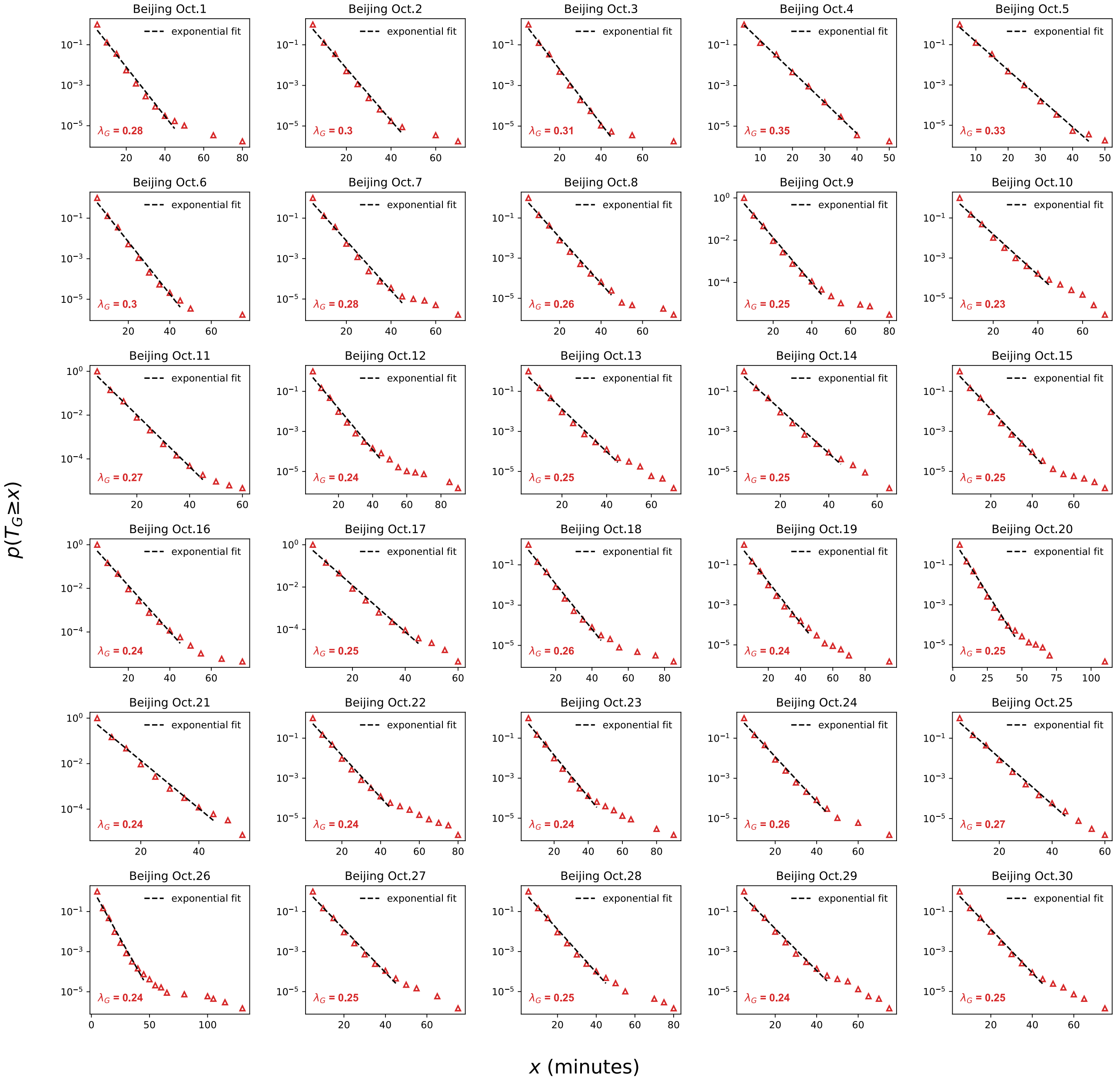

Fig. R11. The CCDF of the growth duration T_G in log-linear plots for 30 days of October 2015 in Beijing. The main parts of the CCDF of growth duration are well fitted by exponential distribution. The tail of the CCDF seems to be a little fatter than the exponential distribution. The exponential distributions and their exponents λ are similar for the same type of days. October 1 to October 7 are seven holidays of China's National Day, Oct. 11, 17, 18, 24, and 25 are the regular weekends, and the other 18 days are the workdays.

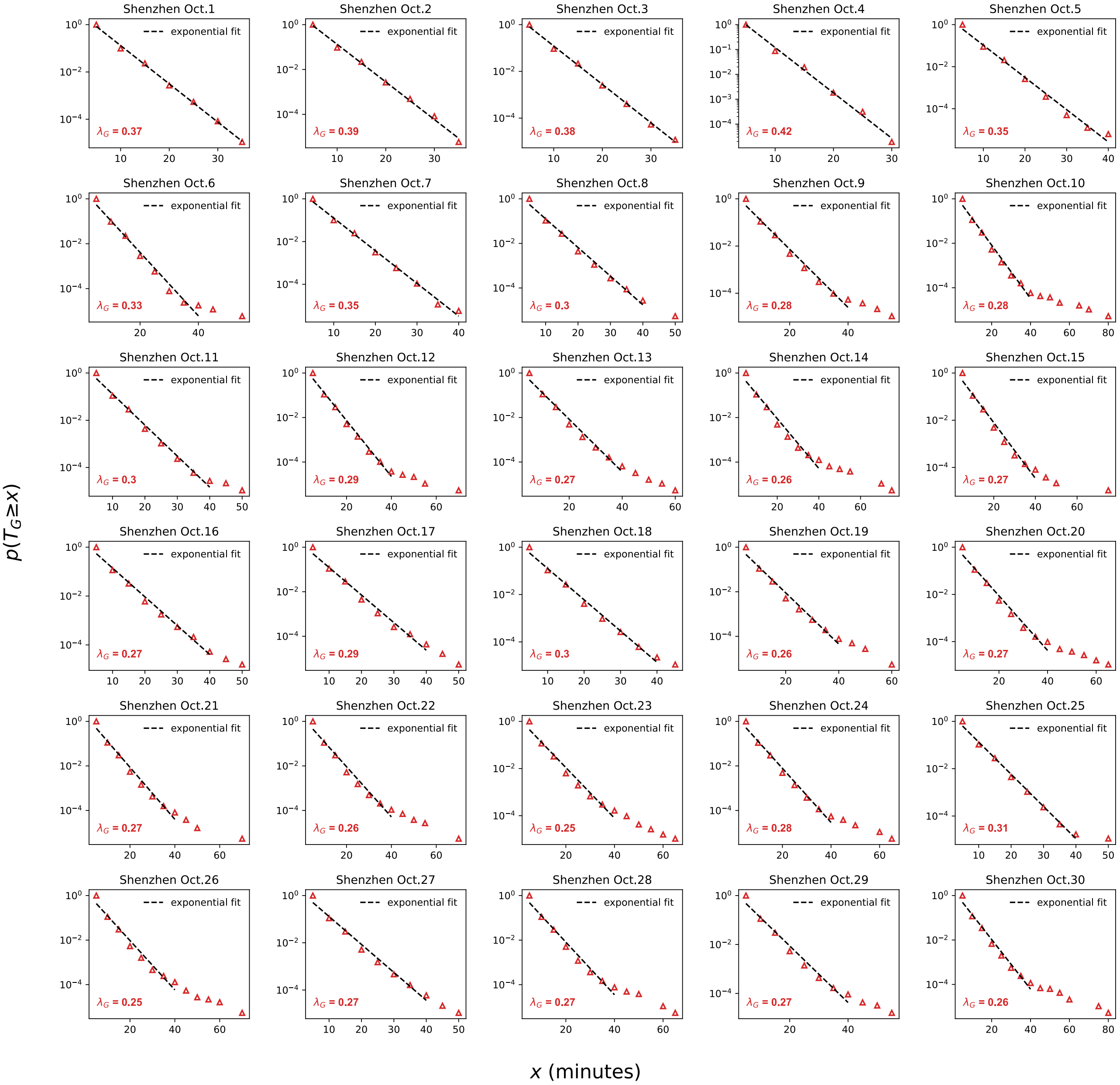

Fig. R12. The CCDF of the growth duration T_G in log-linear plots for 30 days of October 2015 in Shenzhen. The main parts of the CCDF of growth duration are well fitted by exponential distribution. The tail of the CCDF seems to be a little fatter than the exponential distribution. The exponential distributions and their exponents λ are similar for the same type of days. October 1 to October 7 are seven holidays of China's National Day, Oct. 11, 17, 18, 24, and 25 are the regular weekends, and the other 18 days are the workdays.

R1C4: Tables throughout the rebuttal are really hard to read. My greatest concern is about claims relating to T_G. However, the results are presented in 3 tables, each one showing some partial result for each of the variables.

Another problem is the choice of x_min = 15 for T_G. When x_min = 10, one cannot reject the hypothesis that the data could have been drawn from an exponential distribution but rejects (quite categorically) the power law hypothesis).

Moreover, when going from x_min = 10 to x_min = 15, one loses the ability to explain about half the data.

So, I must ask, why are the authors choosing to set x_min = 15?

Response: We thank the reviewer very much for this comment. First, we have, as suggested by the referee, simplified the results in the revised manuscript in a single table (Table R1), to summarize the test of the results on the distribution of growth duration T_G. The results support that the main part of the growth duration could be well approximated by exponential distributions. Interestingly, we find that the values of the exponential exponent λ_G are very similar for the same type of days (e.g., holidays or workdays) (Fig. R**3**). Second, we agree with the reviewer, and noted in the revised manuscript, that focusing on data above 15 minutes could neglect the main part of the data. As shown in Figs. R**10** to R**12** in response to R1C3, we have now included most of the data in fitting survival functions of growth duration and we fully agree with the reviewer that the main part of the growth duration could be well approximated by exponential distributions. We thank the reviewer for this excellent suggestion and we have updated these results in Supplementary Table 1 and Supplementary Figs. 10 and 11 of the revised SI.

Table R1. The *p*-values for the fitted exponential and power-law distributions of growth duration, on Oct. 16, 2015 in Beijing and Shenzhen. The *p*-value is calculated based on the fittings of T_G above 10 minutes. If *p* is large (commonly larger than 0.1) (Clauset et al., 2009), the distance between the empirical data and the estimated curve can be considered as statistical fluctuations; if it is smaller than 0.1, the model may not be suitable to fit the empirical data. *Note that the p-values for the exponential model of growth duration are 0.126 for Beijing and 0.191 for Shenzhen, supporting that the exponential model could well describe the main part of the distribution of* T_G. *In contrast, for the power law assumption, the p-values are much smaller than 0.1. As shown in Fig. R3 (Fig. 2 in the revised manuscript), the values of* λ_G *are very similar for the same type of days in the same city but different for different cities.*

	Exponential	Power law
Beijing	**0.126**	0.003
Shenzhen	**0.191**	0.018

R1C5: I really do not find the answer to my comment R2C3 convincing. There is really no difference between estimates for beta_R during the weekends and weekdays.

Response: We appreciate very much the reviewer's careful examination. We have corrected in the revised manuscript this inaccurate statement and claim now that β_R in weekends and weekdays are not significantly different.

Reviewer #2 (Remarks to the Author):

The authors have fully addressed each of my original comments sufficiently. I have no further comments on the manuscript.

Response: We are pleased that our additional works have addressed all the concerns of the reviewer.

Reviewer #3 (Remarks to the Author):

Authors have provided appropriate response and actions to my few major comments. I believe that these changes though small have solved important issues and substantially improved the manuscript. In particular, authors have done a good job with the removal of loose interpretations, tightening the logic behind their conclusions, and diving deeper in the analysis of the physics of congestion.

The propagation and dissipation of traffic has been long studied but merely in road/highway level. Studies such as this submission are much needed to deepen our understanding of network level congestion dynamcis. Overall, I recomemnd publication of this submission but encourage the authors to consider my remaining comments below.

Response: We thank the reviewer very much for the helpful comments, and for considering our additional work and revisions "solved important issues" and "substantially improved the manuscript". We have carefully considered all the new comments, and revised the manuscript accordingly. Below we provide the point-by-point responses.

R3C1: - I had a comment suggesting elmination of DTB (since it is not adding anything to the concept of bottleneck) and using "bottlenecks" instead which is easy to understand for any reader. Authors seemed to agree with me in their response but there are many usages of DTB still in the revised manuscript.

Response: We thank the reviewer very much for the careful examination. We have thoroughly checked the manuscript and eliminated all expressions of DTB.

R3C2: - The analysis of Supp Fig. 17 is very interesting to me, and might be for other readers as well, so it may deserve a bit more coverage. I suggest authors add one or two sentences to their expalanation in the main text.

Response: We thank the reviewer very much for this kind comment. We have added a discussion in Section 2.2 of the Main Text to explain the corresponding findings in SI. We copy the revisions in the following:

"*The values of <r> could be influenced by the heavy tails of the recovery duration (**Supplementary Note** 7 and **Supplementary Fig. 23**). The "critical slowing down" effect* [58] *becomes more significant for those bottlenecks with large recovery duration. This could be related to the fact that nonlinear mechanisms exist between the congestion propagation and dissipation processes given the complex urban traffic network topologies and the travelers' real-time self-adaptive route choice behaviors*[58, 63, 64]."

R3C3: - In the new version of the manuscript, a big paragraph is dedicated to review of the literature on congestion mechanisms from Transport Engineering and Classical Physics. I, however, did not find it an improvement. The reviewed literature are deeply focused on the

mechanisms occuring inside a road space (a road segment or highway) and they do not explain the phenomena in a network or roads--thus irrelevant to the contributions of this paper.

Response: We thank the reviewer very much for this comment. We now simplify and reduce these contents in the Introduction of Main Text. We move the detailed contents of these references to Supplementary Note 1 of the revised SI. We also copy here the simplified contents in the main text:

"*Some of them investigated the spontaneous occurrences of congestion, including the kinematic wave theory*[4, 5]*, the cellular automaton (CA) models*[6, 7] *and the three-phase traffic theory*[8-10]*. Attention is also paid to understanding the jam formation for the known causes, including the queue model*[11]*, the lane-changing model*[12-14] *and the cell transmission model (CTM)*[15, 16] *(see **Supplementary Note 1**).*"

REVIEWERS' COMMENTS

Reviewer #1 (Remarks to the Author):

I thank the authors for taking my suggestions seriously. I think that the results are now on a strong statistical basis.

I do not want to make any other impositions on the authors. However, I cannot resist making a suggestion.

Figures R11 suggest that for most days lambda ~ 0.25 and that the exponential regime ends at t ~ 40.

One could then pool the data from different days together and get a better grasp on what happens for t > 40. Is it a change in the value of lambda or some other behavior.

Responds to the reviewers' comments on manuscript

"Spatiotemporal dynamics of traffic bottlenecks: an early signal of heavy congestions"

by Jinxiao Duan, Guanwen Zeng, Nimrod Serok, Daqing Li, Efrat Blumenfeld Lieberthal*,

Hai-Jun Huang* and Shlomo Havlin*

(Manuscript No.: NCOMMS-23-10587B)

We wish to thank the editor and the anonymous reviewers for their valuable comments and suggestions that helped us to improve our manuscript. Listed below please find our response to all review comments.

Reviewer #1 (Remarks to the Author):

I thank the authors for taking my suggestions seriously. I think that the results are now on a strong statistical basis.

I do not want to make any other impositions on the authors. However, I cannot resist making a suggestion.

Response: We thank the reviewer very much for the helpful comments. We have carefully considered the new comments. Below we provide our point-by-point responses.

R1C1: Figures R11 suggest that for most days lambda ~ 0.25 and that the exponential regime ends at t ~ 40.

One could then pool the data from different days together and get a better grasp on what happens for t > 40. Is it a change in the value of lambda or some other behavior.

Response: We thank the reviewer very much for this comment. We have accumulated the data for all days in 1 month together and used the exponential models to fit the CCDF of growth duration T_G (**Fig. R1**) above 40 minutes. Note that above 40 minutes, we have less than 10^{-4} events out of the total number of jam components. The figures show that the exponential exponents in the tail become 0.08 for Beijing and 0.12 for Shenzhen, which are significantly smaller than the exponents (close to 0.25) for T_G below 40 minutes. Smaller exponential exponents mean that growth duration decays slower above the crossover point. We have added these figures in the revised Supplementary Fig. 24 and discussed them in the Main Text.

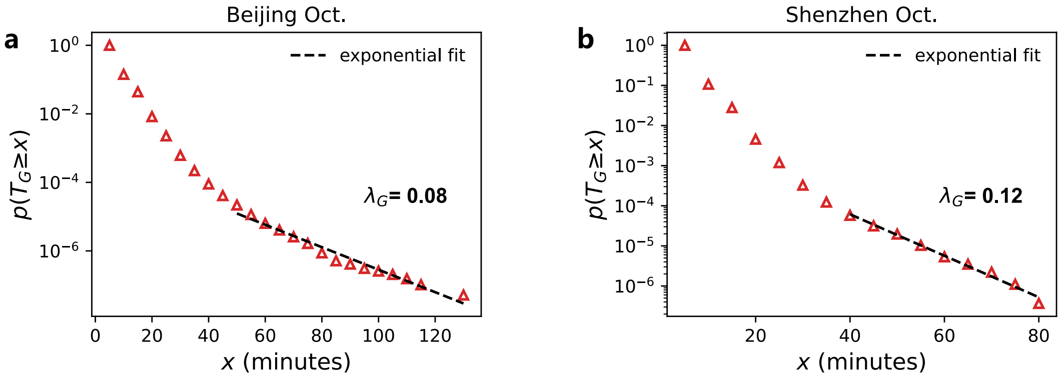

Fig. R1. CCDF of growth duration in the tail by accumulating the data of 30 days in October 2015 together. a shows the exponential fitting of CCDF of growth duration above 50 minutes in Beijing and **b** shows the exponential fitting of CCDF of growth duration above 40 minutes in Shenzhen. Note that we find a suitable crossover time is 50 minutes in Beijing and 40 minutes in Shenzhen. By the suitable crossover time here, both regimes are well-fitted.
